# Naphthalimide-fused BODIPYs *via* one-pot quadruple C–H activation: ultra-low LUMO n-type semiconductors with efficient near-infrared emission

**DOI:** 10.1039/d6sc04883h

**Published:** 2026-08-24

**Authors:** Heng Li, Qi Zhang, Rui Gan, Fan Ni, Qinghua Wu, Huiquan Zuo, Hua Wang, Erhong Hao, Lijuan Jiao

**Affiliations:** a Key Laboratory of Functional Molecular Solids of Ministry of Education, College of Chemistry and Materials Science, School of Physics and Electronic Information, Anhui Normal University Wuhu 241002 China jiao421@ahnu.edu.cn haoehong@ahnu.edu.cn; b School of Instrument Science and Optoelectronic Engineering, Hefei University of Technology Hefei Anhui 230009 P. R. China; c School of Pharmacy, Anhui University of Chinese Medicine Hefei 230012 China

## Abstract

Near-infrared (NIR) n-type organic semiconductors with low-lying LUMO levels are essential building blocks for advanced optoelectronics, yet their development is hindered by laborious multi-step synthesis, insufficient electron affinity and limited stability. Herein, we report the precise synthesis and optoelectronic properties of a series of naphthalimide (NMI)-fused BODIPY derivatives as near-infrared absorbing n-type organic semiconductors, accessed through a palladium-catalyzed one-pot quadruple C–H activation strategy that constructs the fused conjugated framework in a single step. This annulation extends the large π-conjugated system and enables a favorable face-to-face molecular stacking in the crystalline state. The resulting dyes exhibit efficient NIR emission (*Φ* = 0.35–0.43), high molar absorptivity (*ε* up to 1.85 × 10^5^ M^−1^ cm^−1^), and excellent photostability. Critically, NMI fusion dramatically lowers the LUMO energy to as low as −4.32 eV, enabling reversible multi-electron reduction. Chemical reduction with cobaltocene generates stable radical anions characterized by a diagnostic NIR absorption and a sharp EPR signal (*g* = 2.003). Solution-processed organic field-effect transistors confirm n-type charge transport. This work establishes a concise, modular synthesis of NMI-fused BODIPYs with an unprecedented combination of ultralow LUMO levels, stable radical anions, and efficient NIR emission, providing a versatile platform for organic electronics and beyond.

## Abstract 2

Pd-catalyzed quadruple C–H activation enables the construction of NMI-fused BODIPYs with extended π-conjugation, low-lying LUMO energy levels, high reducibility to form stable anion radicals, and favorable n-type charge transport properties.

## Graphical abstract

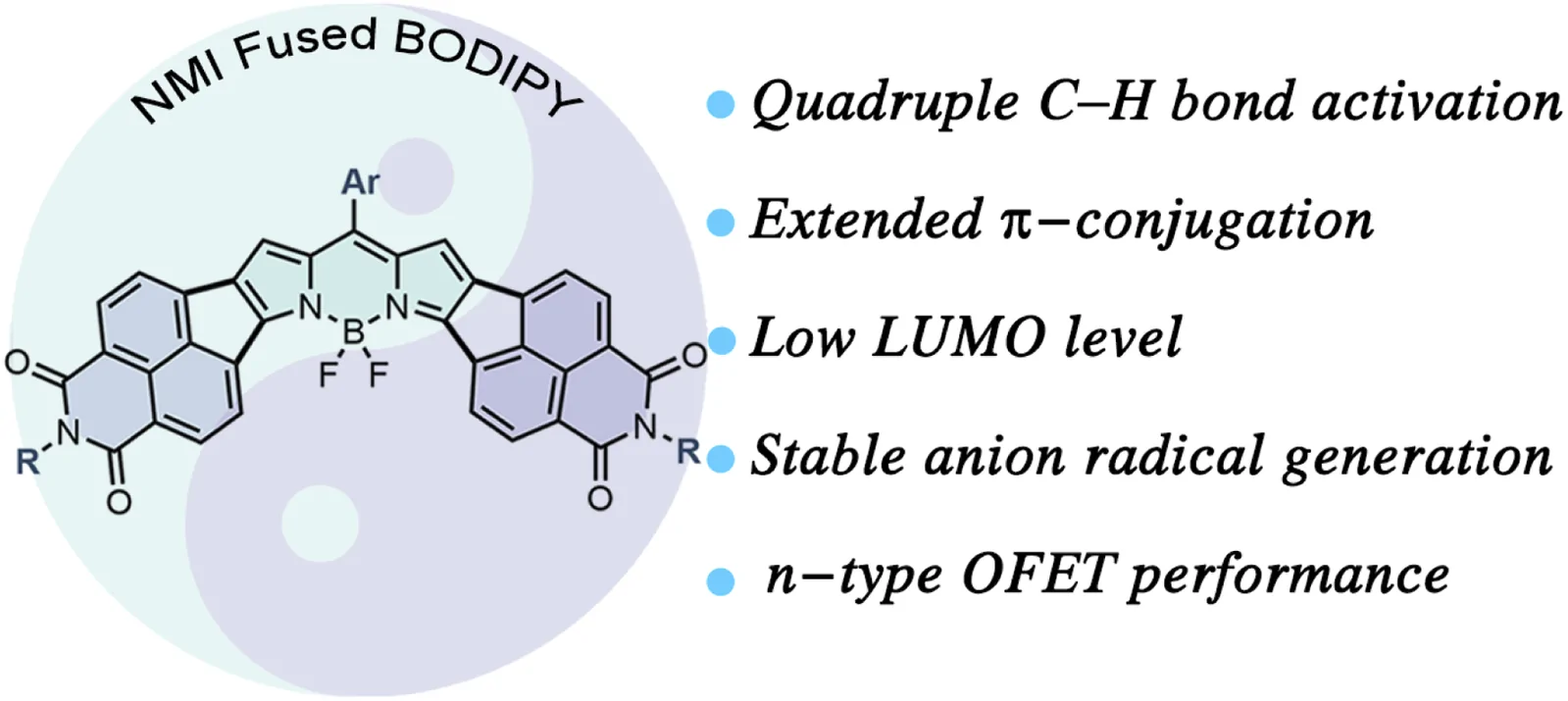

## Introduction

**Fig. 1:**
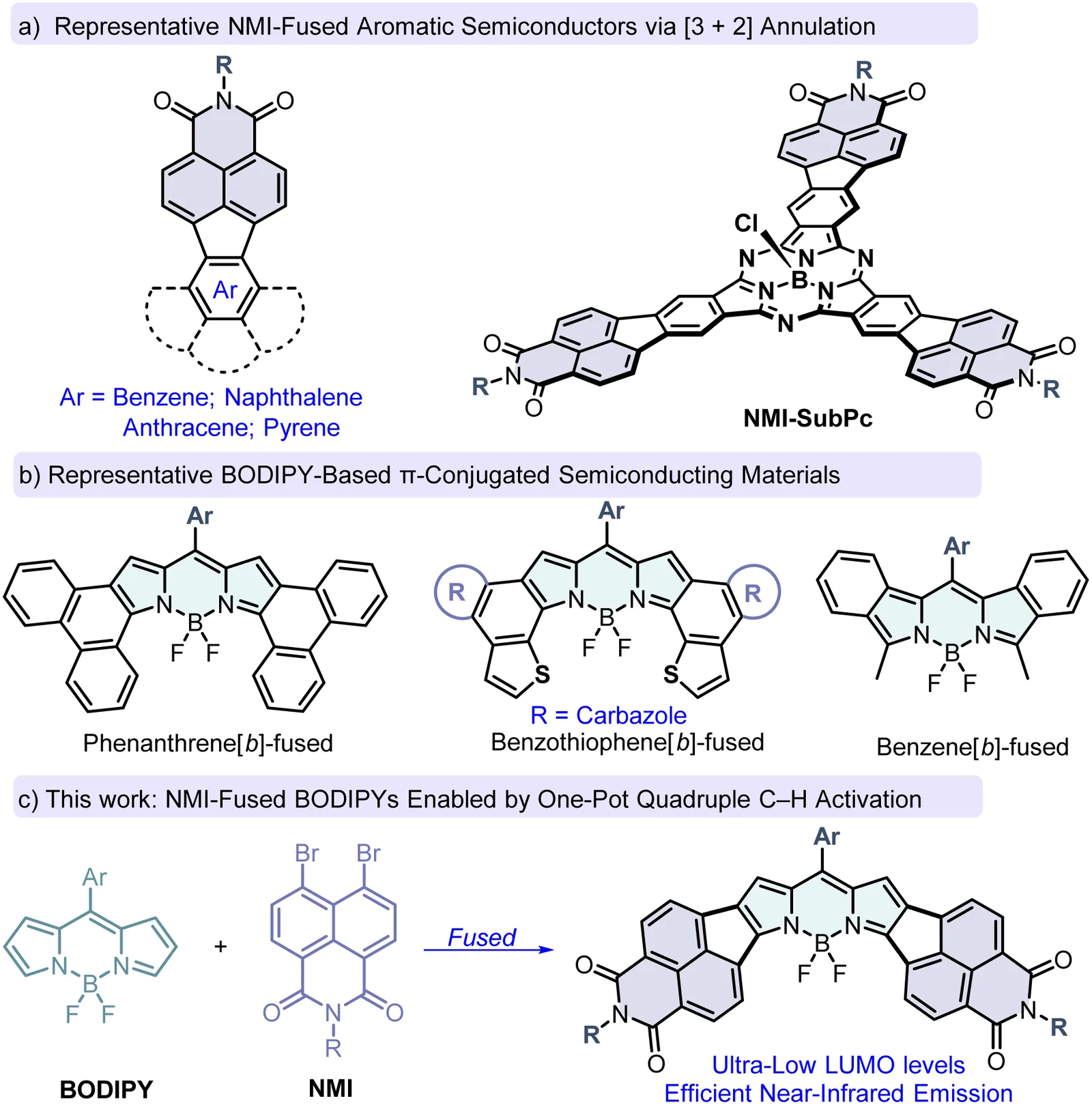
(a) Reported cascade annulation reaction for the fusion of NMI to aromatic cores. (b) Reported aromatic-fused BODIPY derivatives. (c) Molecular structure of BODIPYs and NMI-fused BODIPYs reported in this work.

Small molecular organic semiconductors have emerged as key components for next-generation optoelectronic devices.^1–6^ Among them, near-infrared (NIR) n-type semiconductors with low-lying LUMO levels are especially critical for applications such as bioimaging, organic photovoltaics, and photodetection. However, their development is hindered by the scarcity of highly electron-deficient building blocks, which often leads to multi-step synthesis, insufficiently low LUMO levels, and poor ambient stability.^7–10^ Among the vast library of π-conjugated building blocks, naphthalimide (NMI), a classic electron-deficient building block, has been widely employed to construct n-type organic semiconductors.^11–15^ When fused to aromatic cores (Fig. 1a), NMI units effectively lower the LUMO, enhance electron affinity, promote ordered π–π stacking, and maintain good solution processability.^16–21^ For example, Würthner and co-workers^22–24^ have demonstrated that NMI-fused aromatic systems exhibit stabilized low-lying LUMO levels and favorable supramolecular self-assembly, making them promising candidates for organic photovoltaics and field-effect transistors.

In parallel, boron dipyrromethene (BODIPY) dyes^25–34^ are distinguished by their tunable absorption and emission, high molar extinction coefficients, reversible redox behavior, excellent thermal stability and photostability. To further extend π-conjugation, narrow the optical bandgap and improve charge transport, aromatic-fused BODIPY derivatives (Fig. 1b), have been actively pursued.^35–44^ Despite these advances, conventional fused BODIPYs still suffer from two major limitations: (i) their LUMO levels are often insufficiently low for efficient electron injection and stable anion formation, which limits their performance as n-type semiconductors, and (ii) their synthesis typically relies on laborious multi-step pre-functionalization (*e.g.*, halogenation, borylation, Stille/Suzuki couplings) followed by cyclization, leaving a pressing need for more efficient synthetic strategies.^45,46^

A promising yet unexplored solution to both challenges lies in the direct fusion of NMI onto the BODIPY core. Although NMI-fused aromatics and aromatic-fused BODIPYs have each been independently developed, the integration of these two design principles into a single molecular skeleton as a BODIPY-NMI hybrid has remained elusive. This hybrid design is conceived to synergistically combine the deep-lying LUMO and strong electron-withdrawing character of NMI with the robust optical properties, structural rigidity, and high absorptivity of BODIPY. The resulting NMI-fused BODIPYs are expected to deliver ultra-low LUMO levels, stable radical anions, enhanced molecular planarity, and efficient NIR emission. The combination of features is highly desirable for organic electronics but rarely achieved in a single scaffold. Moreover, through a rational synthetic design based on one-pot Pd(ii)-catalyzed C–H activation,^47,48^ this hybrid can be assembled directly from unfunctionalized BODIPY and brominated NMI precursors^13,17,24^ (Fig. 1c), circumventing tedious pre-functionalization and providing a modular, atom-economical route.

Herein, we report the design, synthesis and systematic characterization of a series of NMI-fused BODIPY derivatives. Employing a quadruple C–H activation cascade, we achieve the dual fusion of NMI units onto the BODIPY periphery in a single step. The resulting π-extended hybrids exhibit significantly lowered LUMO levels, form stable radical anions upon chemical reduction, and display efficient NIR emission (*Φ* up to 0.43) with high molar absorptivity (*ε* up to 1.85 × 10^5^ M^−1^ cm^−1^). Solution-processed organic field-effect transistors based on these materials demonstrate typical n-type charge transport, validating their potential as low-LUMO n-type semiconductors. This work provides a concise, atom-economical route to a new class of fused BODIPYs and highlights NMI fusion as a strategy to achieve a desirable combination of low LUMO levels, stable radical anions, and efficient NIR emission.

## Results and discussion

### Synthesis and characterization

**Scheme 1:**
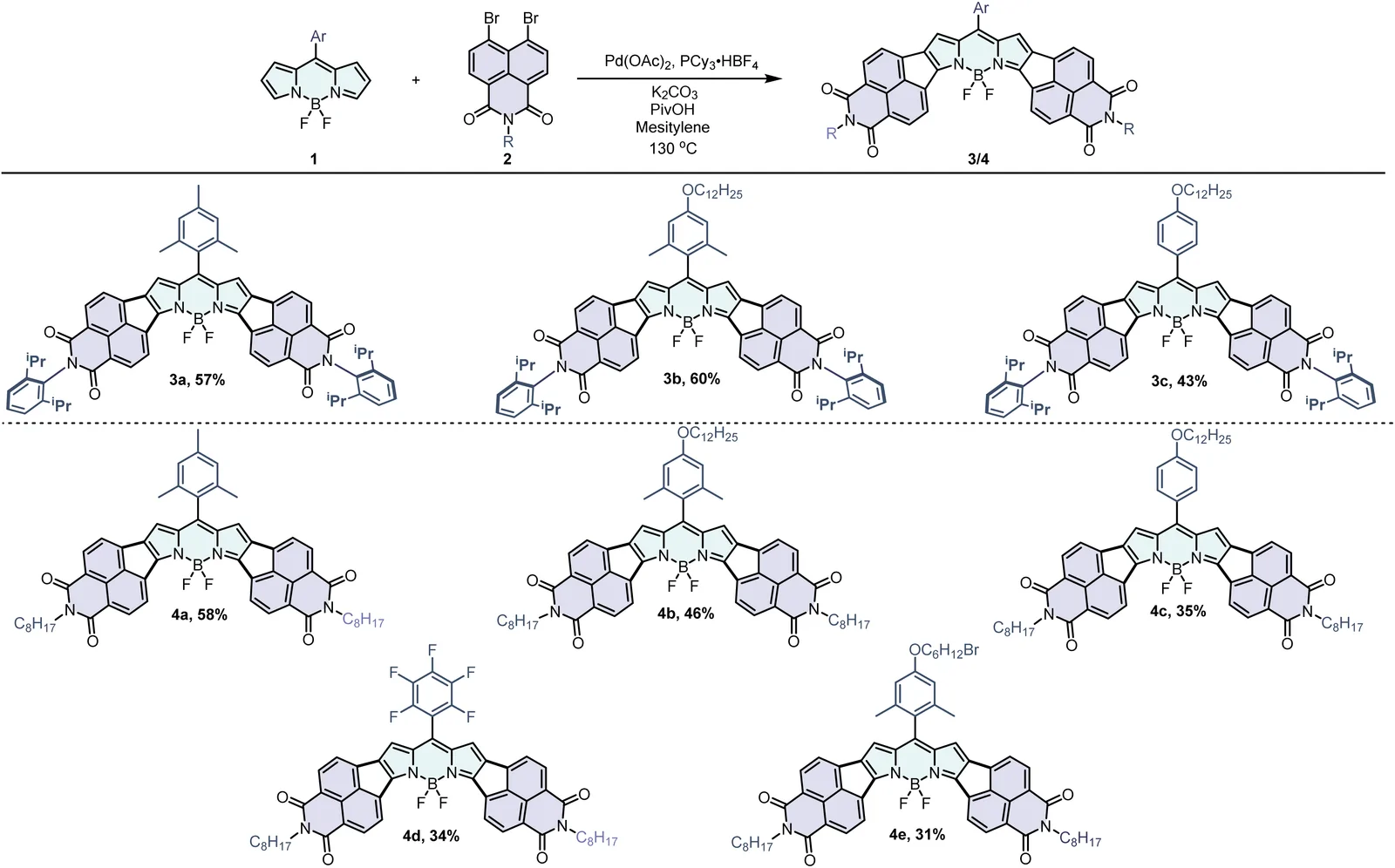
Synthesis of NMI-fused BODIPYs 3a–3c and 4a–4e*via* a one-pot Pd(ii)-catalyzed quadruple C–H activation cascade.

**Fig. 2:**
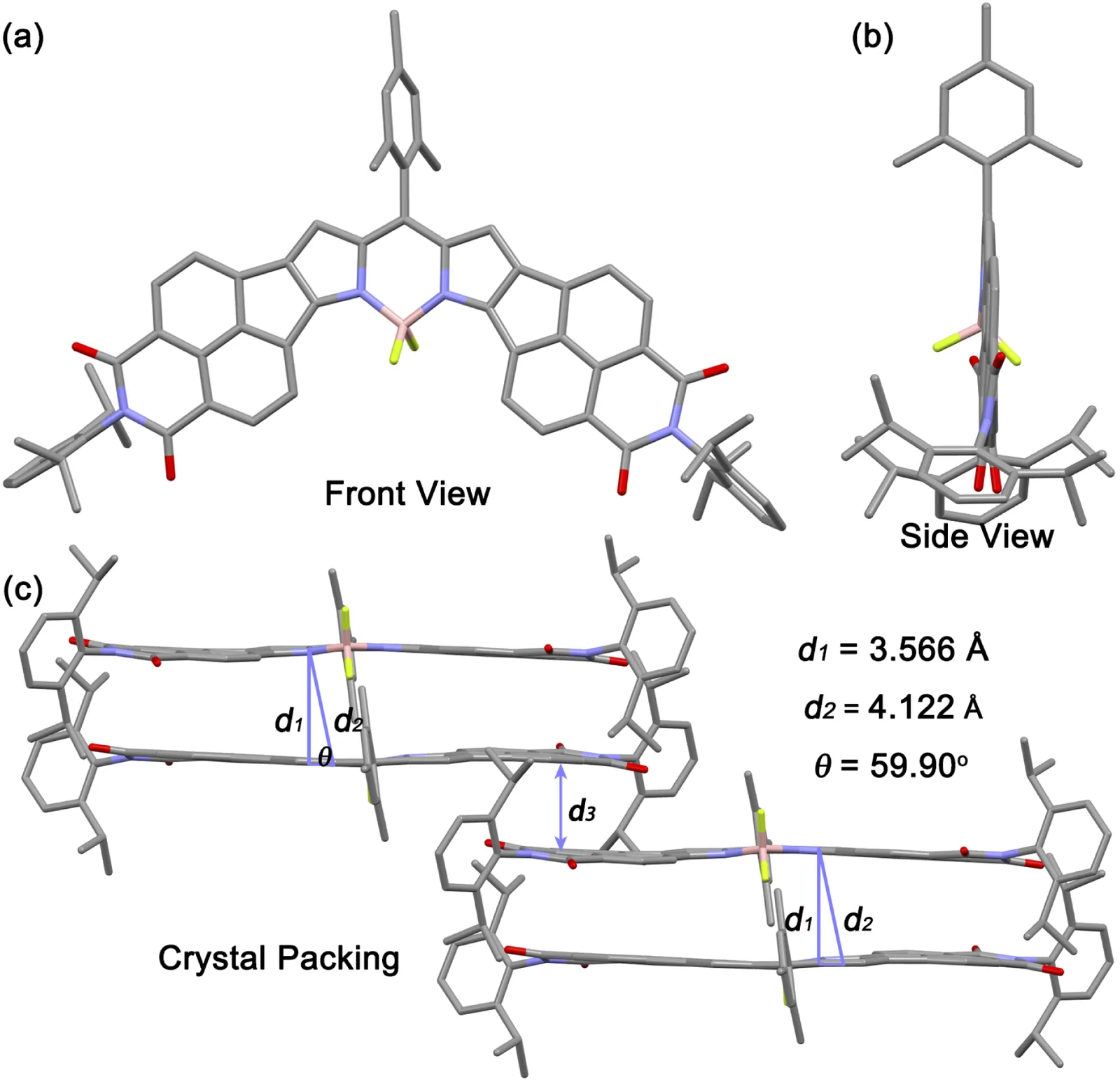
Front (a) and side (b) views of the X-ray crystal structures of 3a with wireframe. (c) Crystal packing of X-ray structure of 3a. C, gray; N, blue; B, pink; F, green; O, red. Hydrogen atoms were removed for clarity.

To test the above strategy (Fig. 1c), we first examined the reaction of *meso*-substituted BODIPY 1a (0.1 mmol) with dibrominated NMI 2a (4 equiv.) in mesitylene (3 mL) under an argon atmosphere at 130 °C, using Pd(OAc)_2_ (0.5 equiv.), PCy_3_·HBF_4_ (1 equiv.), and K_2_CO_3_ (4 equiv.) as the base (Scheme 1). After 12 h, TLC analysis showed complete consumption of 1a and the formation of a new, blue colour product 3a. The desired NMI-fused BODIPY 3a was isolated in 57% yield after column chromatography. The structure was unambiguously confirmed by ^1^H and ^13^C NMR, high-resolution mass spectrometry (HRMS), and single-crystal X-ray diffraction (Fig. 2).

With the optimized conditions in hand, we explored the substrate scope with respect to both the BODIPY core and the NMI precursor. Two additional *meso*-aryl substituted BODIPYs 1b–1c bearing with long –C_12_H_25_ alkyl chains with or without 2,6-dimethyl groups were tested, and both underwent the cascade annulation with NMI 2a smoothly, providing the corresponding NMI-fused products 3b–3c in 60% and 43% yields, respectively. Furthermore, dibrominated NMI 2b with long –C_8_H_17_ alkyl chain was selected to react Various *meso*-aryl substituted BODIPYs 1a–e, all smoothly providing the corresponding NMI-fused products 4a–4e in 31–58% yields. The bulky substituents or long alkyl chains on *meso*-position of BODIPY or N atom of NMI proved essential to ensure good solubility of the final products.^49,50^ All NMI-fused BODIPY derivatives exhibited excellent ambient stability and were fully characterized by NMR spectroscopy and HRMS.

The suitable X-ray crystals of 3a for X-ray analysis were grown by slow diffusion of hexane into its dichloromethane solution. Structural analysis unambiguously verified the successful construction of the NMI-fused BODIPY rigid skeleton *via peri*-fusion cyclization (Fig. 2a, b and S3). Analogous to previously documented planar BODIPY-based π-conjugated materials, the entire fused framework of 3a adopts a highly coplanar conformation.^48^ The dihedral angles between the BODIPY parent core and the fused NMI aromatic segment were determined to be merely 3.32° and 4.19°, respectively (Table S1). Such negligible torsional distortion demonstrates strong intramolecular π–π conjugation and effective orbital delocalization over the entire fused aromatic system. The newly formed C–C covalent bonds bridging the BODIPY motif and NMI unit were measured to be 1.47 Å, 1.47 Å, 1.45 Å and 1.46 Å (Table S1). These moderate bond lengths, falling between conventional C–C single bonds and conjugated C=C double bonds, further confirm the formation of a stable and fully conjugated fused-ring architecture. Benefiting from the highly planar molecular skeleton, compound 3a exhibits prominent face-to-face π–π stacking in the solid state. The vertical stacking distance is 3.57 Å with a slip angle of 59.90° (Fig. 2c), which is indicative of efficient intermolecular orbital overlap. This compact slipped stacking mode suggests favorable intermolecular orbital overlap, which is generally beneficial for charge migration in organic semiconductors.

### Spectroscopic properties

**Fig. 3:**
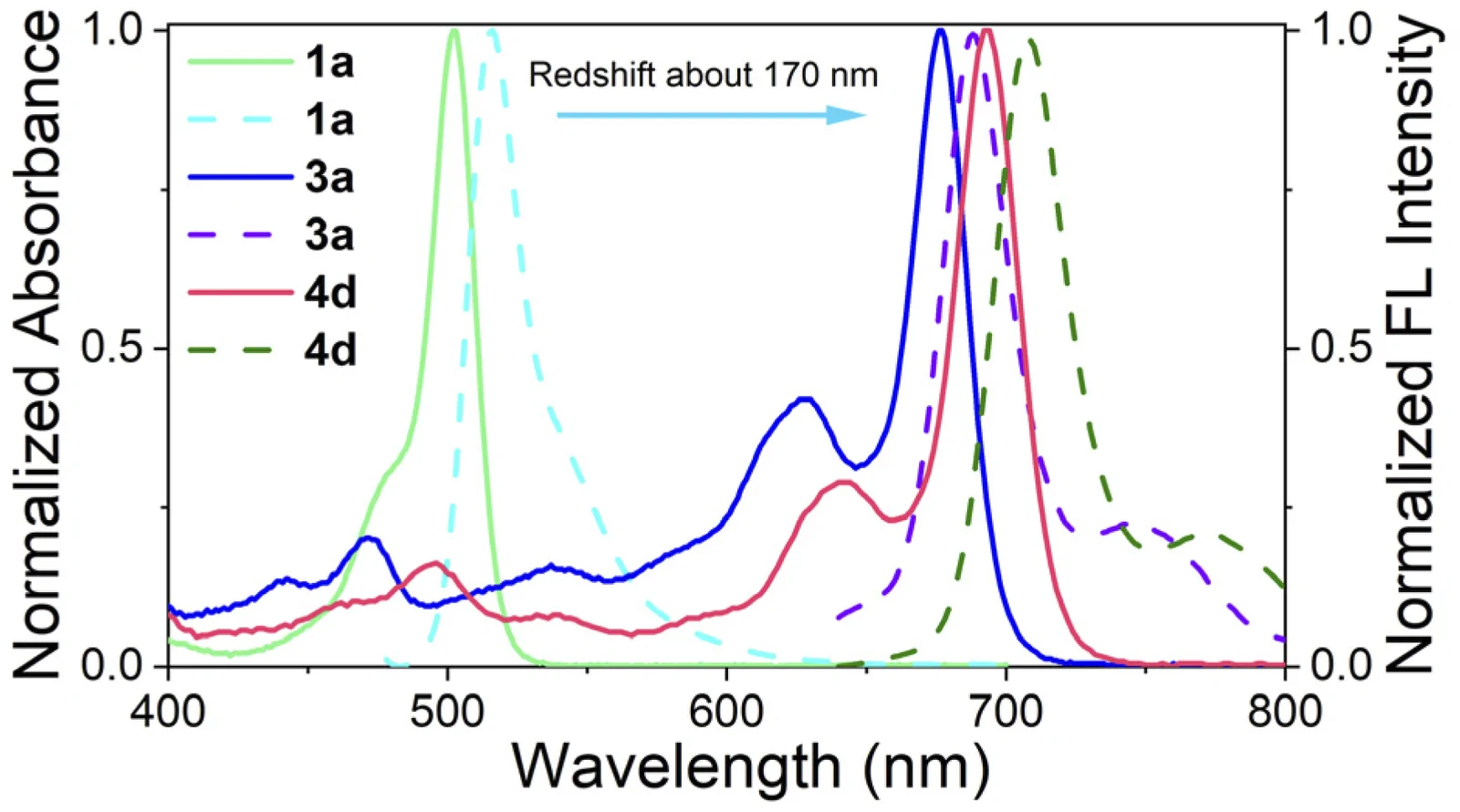
Normalized absorption (solid line) and emission spectra (dash line) of 1a, 3a, and 4d in toluene.

The photophysical properties of the resulting NMI-fused BODIPY derivatives 3a–3c and 4a–4e were systematically investigated, and the relevant data are summarized in Tables 1 and S3. As shown in Fig. 3 and S4–11, all fused compounds exhibit intense absorption and favourable fluorescence emission covering the red to NIR region in solutions. Their maximum absorption wavelengths are located at 672–692 nm, while the emission maxima range from 685 to 708 nm. Compared with the parent BODIPY 1a, the *peri*-fusion of electron-deficient NMI segments onto the BODIPY core produces a remarkable spectral red-shift of approximately 170 nm (Fig. 3). Meanwhile, these fused derivatives possess greatly improved molar extinction coefficients, with the maximum value up to 1.85 × 10^5^ M^−1^ cm^−1^. In toluene, the target compounds exhibit good fluorescence quantum yields in the range of 0.35–0.43, which is notably high for NIR-absorbing organic dyes in this spectral region.

**Table 1:** Photophysical properties for 1a, 3a–3c and 4a–4e in toluene

Dyes	a *λ* ^max^_abs_ (nm)	*ε* b	*λ* ^max^_em_ (nm)	Stokes shift^c^ (cm^−1^)	*Φ* d
1a	503	64 500	518	580	0.94
3a	676	120 200	687	240	0.39
3b	678	138 300	687	190	0.35
3c	672	104 200	682	220	0.41
4a	676	184 900	687	240	0.43
4b	676	169 300	686	220	0.40
4c	672	114 700	685	280	0.42
4d	692	184 900	708	330	0.37
4e	676	126 200	686	580	0.41

a In 1 × 10^−5^ M toluene solution.

b Corresponding to the strongest absorption maximum.

c The Stokes shift values are rounded to the nearest 10 cm^−1^.

d Fluorescence quantum yields were measured by Hammatsu C11347 using integrating sphere. The standard errors are less than 5%.

These prominent photophysical changes originate from the extended π-conjugated framework after ring fusion and the inherent donor–acceptor (D–A) electronic interaction between the electron-rich BODIPY skeleton and electron-withdrawing NMI unit. Notably, the fluorescence quantum yield decreases progressively with increasing solvent polarity. For example, in nonpolar *n*-hexane, the quantum yield of 3a is 0.56, whereas in tetrahydrofuran (a polar solvent) it dramatically drops to 0.12 (Table S3). This trend can be rationalized by the distinct intramolecular charge transfer (ICT) character^51,52^ of these D–A-type NMI-fused BODIPYs. In high-polarity solvents, the highly polarized charge-transfer excited state is effectively stabilized, which promotes non-radiative relaxation pathways and suppresses radiative decay. Consequently, the enhanced ICT effect in polar solvents dissipates the excited-state energy through non-radiative deactivation, leading to a continuous reduction in fluorescence quantum yield.

Photostability is a critical requirement for organic optoelectronic materials, as photodegradation can compromise device lifetime and performance. The photostability of representative compound 3a was evaluated under continuous 50 W white LED illumination, using zinc phthalocyanine (ZnPc) and methylene blue (MB) as reference dyes in the near-infrared region (Fig. S12). After 40 min of continuous irradiation, 3a retained approximately 90% of its initial absorbance. In contrast, ZnPc lost about 50% of its absorbance within 15 min, and MB was almost completely photobleached after only 3 min.

The high photostability of 3a can be attributed to its rigid, fully fused conjugated framework, which suppresses photodegradation pathways associated with molecular torsion and excited-state structural relaxation. In addition, the electron-deficient naphthalimide units enhance the electronic stability of the extended π-system, reducing susceptibility to photoinduced oxidation or bond cleavage. These results, together with the ultra-low LUMO level and efficient NIR emission, support the potential of NMI-fused BODIPYs for applications requiring long-term stability.

### Electrochemical properties

**Fig. 4:**
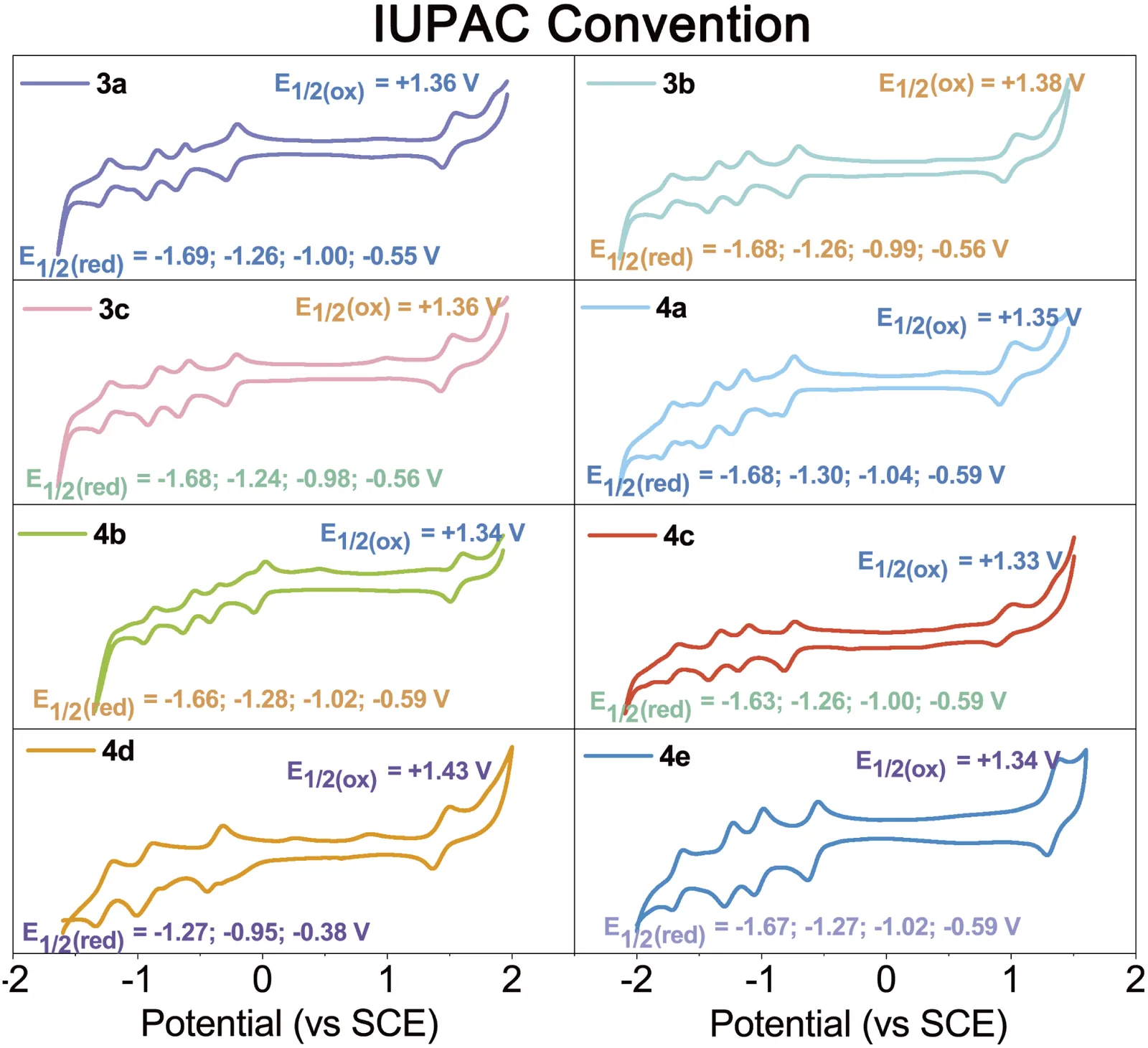
Cyclic voltammograms of 4.0 mM 3a–3c, 4a–4e in dichloromethane.

To investigate the influence of NMI fusion on the electronic structure and electrochemical properties of BODIPY derivatives, cyclic voltammetry (CV) was performed on NMI-fused BODIPYs 3a–3c, 4a–4e together with the parent compound 1a (Fig. 4 and S13). In contrast to pristine 1a, which exhibits only a single reversible reduction wave at −0.96 V and no observable oxidation response, all NMI-fused derivatives display multiple reversible reduction couples in the range of −0.55 to −1.69 V and one reversible oxidation couple at 1.33–1.38 V (Table S4). This marked difference indicates that NMI fusion significantly enriches the redox behavior and enhances the electron-transport capability of the BODIPY core.

Taking 3a as a representative example, the NMI-fused system shows a slightly positively shifted first reduction potential compared to 1a, while the oxidation potential remains largely unchanged. Such distinct electrochemical behavior originates from the strong electron-withdrawing nature of the NMI moiety, which modulates the electronic configuration of the BODIPY skeleton. Notably, 4d, bearing a *meso*-pentafluorophenyl group, exhibits three reversible reduction pairs with a first reduction potential at −0.38 V and an oxidation potential at 1.43 V. Relative to 4a, 4d possesses a less negative reduction potential and a more positive oxidation potential, making it more susceptible to reduction but harder to oxidize due to a consequence of the synergistic electron-withdrawing effect of NMI fusion and pentafluorophenyl substitution.

Consistent with the redox potential variations, NMI fusion substantially lowers the LUMO energy level and strengthens electron affinity. The parent BODIPY 1a has a LUMO of −3.53 eV, whereas NMI-fused 3a exhibits a much deeper LUMO of −3.92 eV; 4d reaches −4.32 eV, the lowest among all derivatives (Table S4). Overall, NMI incorporation not only extends π-conjugation and enriches the reversible redox behavior, but also lowers the frontier orbital levels, leading to enhanced electron affinity and charge-transfer characteristics.

### Theoretical calculations

**Fig. 5:**
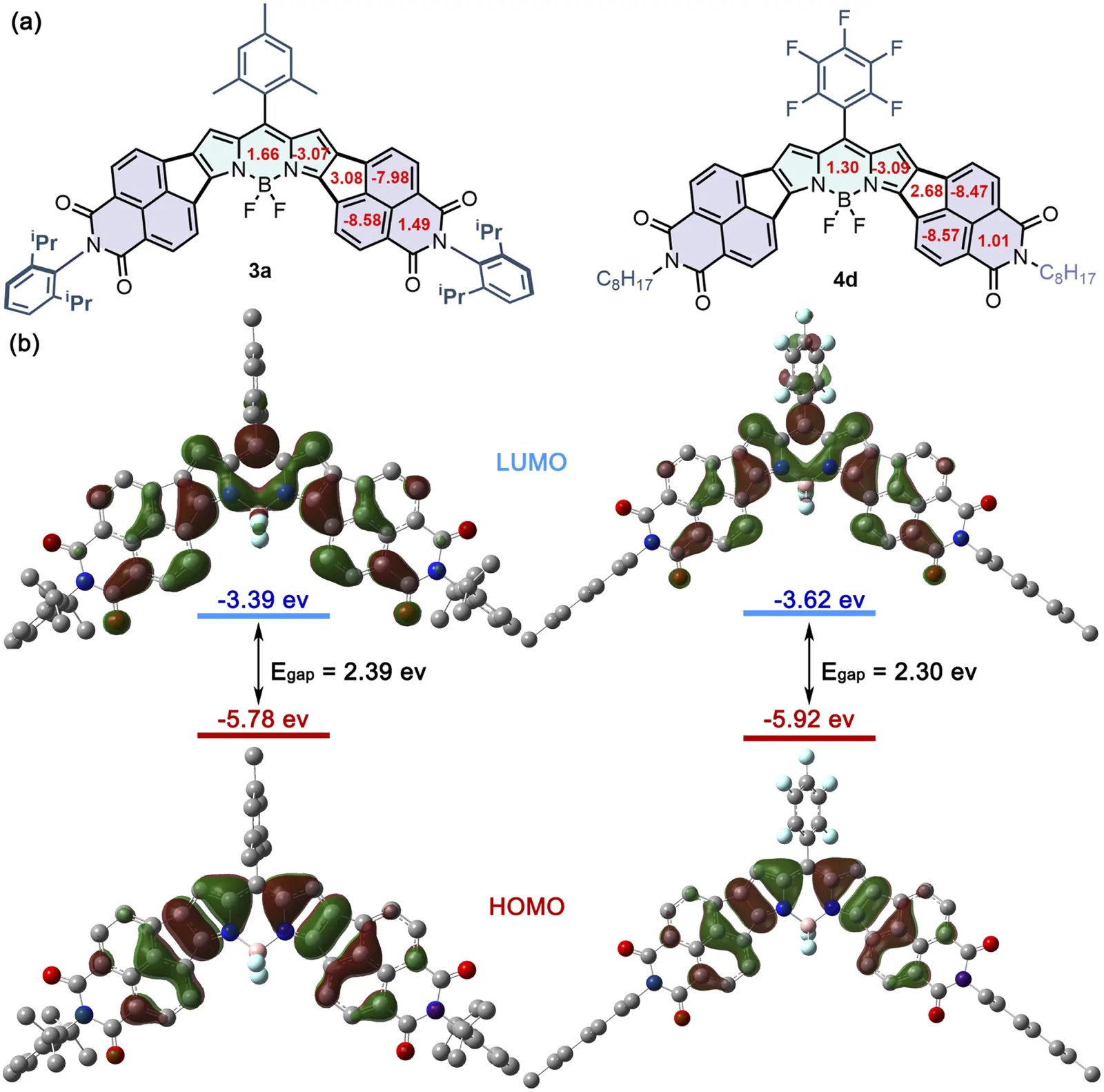
(a) Calculated NICS(1)zz values at various positions of 3a and 4d. (b) LUMO, HOMO, and their energy levels for 3a and 4d (H atoms omitted for clarity).

To gain further insight into the molecular geometries, frontier orbital distributions, and electronic transitions, density functional theory (DFT) and time-dependent DFT (TD-DFT) calculations were performed on representative NMI-fused BODIPYs 3a and 4d. The molecular orbitals of both compounds are mainly delocalized over the BODIPY core and the NMI groups attached at the 3,5-positions. The calculated HOMO/LUMO energy levels of 3a are −5.78/−3.39 eV, while those of 4d are −5.92/−3.62 eV (Fig. 5b). Notably, fusion of the electron-deficient NMI units induces a significant downshift of the frontier orbital levels, thereby narrowing the bandgap. In agreement with electrochemical data, the substantially lowered LUMO levels are comparable to those of well-established n-type semiconductors such as perylene bisimides.^53^

TD-DFT calculations on 3a reveal that the intense lowest-energy absorption band is dominated by the HOMO → LUMO transition, with an S_0_ → S_1_ excitation wavelength of 629 nm (Table S5). Additionally, the experimental shoulder peak at 624 nm can be tentatively assigned to the S_0_ → S_2_ transition (Fig. 3), which is calculated to occur at 573 nm. To evaluate the impact of NMI fusion on local aromaticity, we computed nucleus-independent chemical shift (NICS) values of 3a and 4d (Fig. 5a, and S14). After fusion with the NMI moiety, the pyrrolic five-membered rings in the BODIPY core of 3a/4d show NICS(1)zz values of −3.07/−3.09 ppm, indicating moderately reduced aromaticity. For the two six-membered rings fused to the NMI units, the NICS(1)zz values are −7.98/−8.58 and −8.47/−8.57 ppm for 3a and 4d, respectively, suggesting prominent aromatic character.

### Chemical reduction of radical anion

**Fig. 6:**
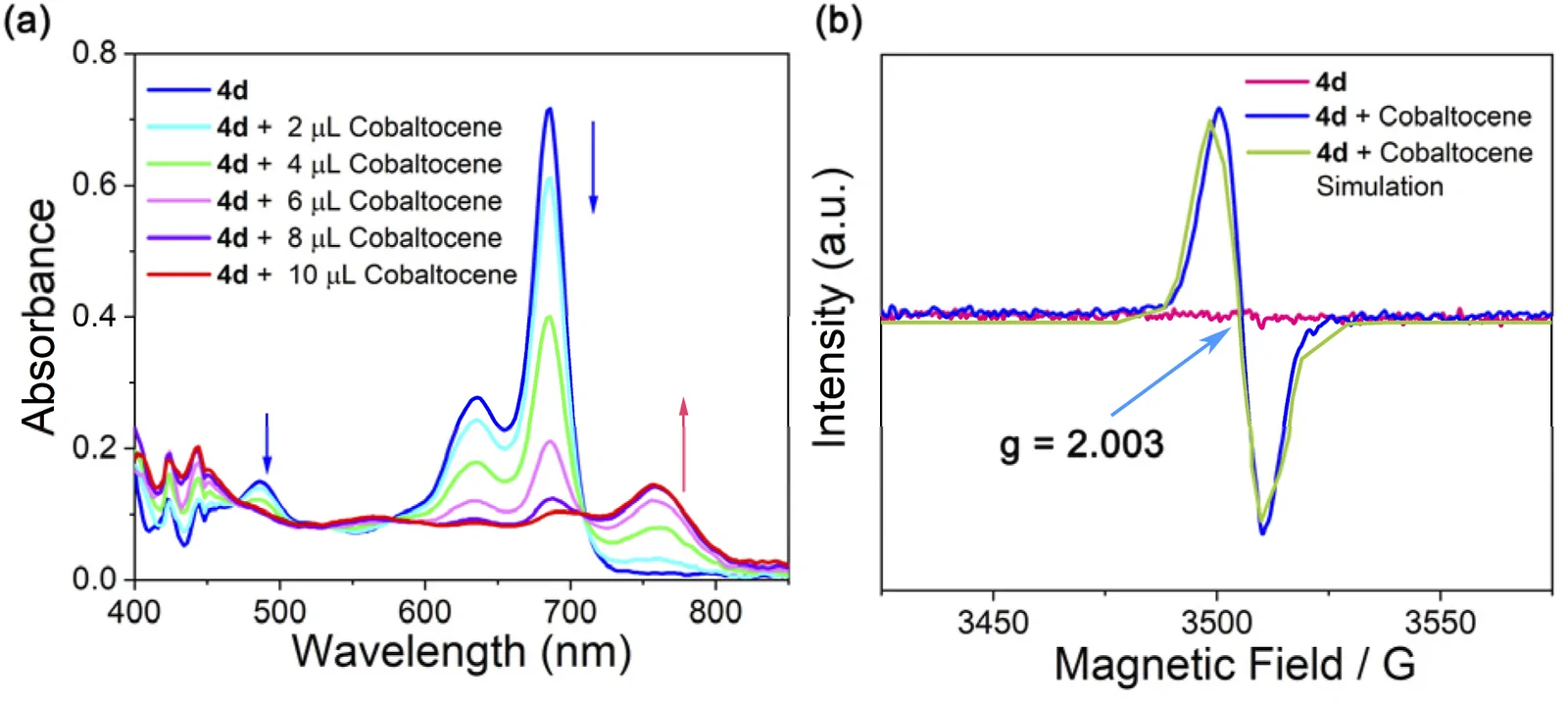
(a) Absorption spectra of 4d in THF with the addition of cobaltocene (0.025 M). (b) EPR spectra of the reduced species obtained with 0 (red line) and 20 (blue line) equiv. of cobaltocene at room temperature.

Encouraged by the low-lying LUMO level of 4d (−4.32 eV from CV) and its favorable electrochemical reduction behavior, we investigated its ability to form a stable radical anion upon chemical reduction.^54,55^ Cobaltocene (CoCp_2_, *E*^o^ = −1.3 V (ref. 56)) was employed as a mild electron donor, and the reduction process was monitored by *in situ* UV-vis-NIR and EPR spectroscopy (Fig. 6). Gradual titration of a CoCp_2_ solution (0.025 M) into a dilute THF solution of 4d (4 µM) under ambient conditions led to the appearance of a new, broad absorption band at 758 nm, while the neutral absorption maximum at 685 nm gradually decreased (Fig. 6a). This significant bathochromic shift is characteristic of radical anion formation and indicates an altered electronic transition upon electron injection.

EPR spectroscopy further confirmed the generation of the radical anion. A sharp, symmetric single EPR signal with a *g*-factor of 2.003 was observed upon addition of CoCp_2_ (Fig. 6b). The absence of hyperfine splitting suggests that the unpaired electron is highly delocalized over the entire conjugated skeleton of 4d. Together, these results demonstrate that the low LUMO level of 4d enables efficient electron capture from CoCp_2_ and the formation of a stable radical anion under ambient conditions.

### Field-effect transport properties

**Fig. 7:**
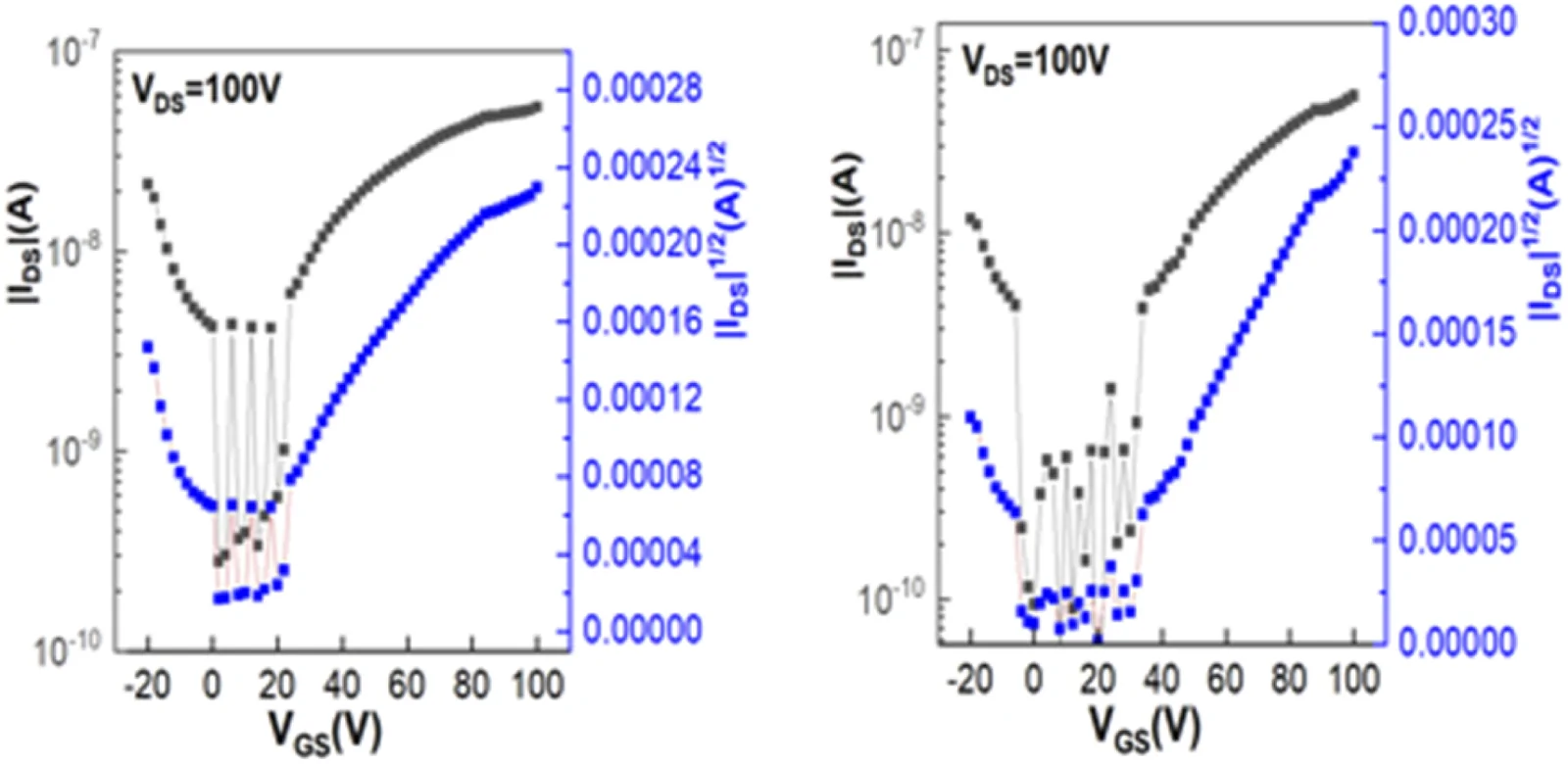
Representative *I*_DS_–*V*_GS_ transfer curves of 3b (left) and 3c (right) OFETs at *V*_DS_ = 100 V.

To evaluate the charge transport properties of the NMI-fused BODIPY derivatives, top-contact organic field-effect transistors (OFETs) were fabricated using 3b and 3c, which bear long alkyl chains to enhance solution solubility. Thin films were prepared by spin-coating, and the effect of thermal annealing (room temperature, 180 °C, 210 °C, and 240 °C) on film morphology and device performance was examined by optical microscopy (OM) and polarized optical microscopy (POM) (Fig. S15). All fabricated devices exhibited typical n-type charge transport behavior, with drain current (*I*_DS_) increasing under positive gate voltages (*V*_GS_) (Fig. 7 and S16).

For 3c-based OFETs, thermal annealing at 180 °C improved film uniformity and molecular ordering, yielding a maximum electron mobility (*µ*_e_, max) of 3.47 × 10^−4^ cm^2^ V^−1^ s^−1^. For 3b-based OFETs, the as-cast film already possessed inherent molecular order, and annealing at 180 °C gave a peak electron mobility of 2.04 × 10^−4^ cm^2^ V^−1^ s^−1^. Both types of devices exhibited on/off current ratios in the range of 10^3^–10^4^, which are typical for solution-processed n-type OFETs.^57–60^ These results demonstrate that NMI-fused BODIPY derivatives can function as n-type semiconductors in OFETs, highlighting their potential for organic electronic applications.

## Conclusions

In summary, a series of naphthalimide (NMI)-fused BODIPY derivatives were synthesized *via* a palladium-catalyzed one-pot quadruple C–H activation cascade. The fused molecules feature extended π-conjugated skeletons and adopt a planar face-to-face π–π stacking in the crystalline state (3.57 Å). They exhibit efficient NIR emission (*Φ* up to 0.43) with high molar absorptivity (*ε* up to 1.85 × 10^5^ M^−1^ cm^−1^). NMI fusion significantly lowers the LUMO level to as deep as −4.32 eV and enables reversible multi-electron reduction. These derivatives form stable radical anions upon chemical reduction with cobaltocene, as evidenced by characteristic NIR absorption and EPR signals. Solution-processed organic field-effect transistors based on these materials show typical n-type charge transport (*µ*_e_ up to 3.5 × 10^−4^ cm^2^ V^−1^ s^−1^). This work provides a concise, atom-economical route to a new class of fused BODIPYs combining ultra-low LUMO levels, stable radical anions, and efficient NIR emission, highlighting their potential as n-type organic semiconductors.

## Author contributions

H. L., Q. Z., R. G., H. Z., and H. W performed the synthetic experiments and data characterization. F. N. performed the OFET analysis. Q. W. performed the theoretical calculations. H. L. wrote the original manuscript. E. H. and L. J. conceived and directed the overall project, revised manuscript. All authors discussed the results and commented on the manuscript.

## Conflicts of interest

There are no conflicts to declare.

## Supplementary Material

SC-OLF-D6SC04883H-s001

SC-OLF-D6SC04883H-s002

## Data Availability

CCDC 2553505 contains the supplementary crystallographic data for this paper.^61^ Supplementary information: experimental details, including detailed synthesis and characterization of all products reported in this study, spectroscopic measurements, electrochemical data, theoretical calculations, OFET characterization, NMR and HRMS spectra of all new products, and X-ray data of 3a. See DOI: https://doi.org/10.1039/d6sc04883h.
