# Supplementary material for: Naphthalimide-fused BODIPYs via one-pot quadruple C–H activation: ultra-low LUMO n-type semiconductors with efficient near-infrared emission
Source: Chem Sci. 2026 Aug 24;17(40):19297–303. doi: 10.1039/d6sc04883h (PMC13509142; doi:10.1039/d6sc04883h)
Supplement: Supplementary file 1 [file SC-017-D6SC04883H-s001.pdf]

Supplementary Information (SI) for Chemical Science.
This journal is © The Royal Society of Chemistry 2026

Supporting Information for

Naphthalimide-Fused BODIPYs via One-Pot Quadruple C–H Activation: Ultra-Low LUMO n-Type Semiconductors with Efficient Near-Infrared Emission

Heng Li,[a] Qi Zhang,[a] Rui Gan,[a] Fan Ni,[b] Qinghua Wu,[c] Huiquan Zuo,[a] Hua Wang,[a] Erhong Hao*[a] and Lijuan Jiao*[a]

[a]*Key Laboratory of Functional Molecular Solids of Ministry of Education, College of Chemistry and Materials Science, School of Physics and Electronic Information, Anhui Normal University, Wuhu, 241002, China. E-mail:*

[b]*School of Instrument Science and Optoelectronic Engineering, Hefei University of Technology, Hefei, Anhui 230009, P. R. China.*

[c]*School of Pharmacy, Anhui University of Chinese Medicine, Hefei, 230012, China.*

**To whom correspondence should be addressed.*

E-mail: jiao421@ahnu.edu.cn, haoehong@ahnu.edu.cn,

Contents

1. General information 1
2. Synthesis and characterization 3
3. Crystal data 11
4. Photophysical data 13
5. Evaluation of the photostability 17
6. Electrochemical data 18
7. DFT calculations 20
8. OFET characterization 29
9. ^{1}H and ^{13}C NMR spectra for all new compounds 32
10. High resolution mass spectra for all new compounds 41
11. Reference 47

1. General information

Reagents and solvents were used as received from commercial suppliers (Energy Chemicals, Shanghai, China) unless noted otherwise. All reactions were performed in oven-dried or flame-dried glassware unless stated otherwise and were monitored by TLC using 0.25 mm silica gel plates with UV indicator (60F-254). ^{1}H and ^{13}C NMR were recorded on a 400 or 500 MHz NMR spectrometer at room temperature. Chemical shifts (δ) are given in ppm relative to $CDCl_3$ (7.26 ppm for ^{1}H and 77.16 ppm for ^{13}C) to internal TMS. All ^{13}C NMR spectra were proton decoupled. High-resolution mass spectra (HRMS) were obtained using APCI or ESI in positive mode.

Absorption and emission measurements. UV-visible absorption and fluorescence emission spectra were recorded on commercial spectrophotometers (Shimadzu UV-2450 and Edinburgh FS5 spectrometers). All measurements were made at 25 °C, using 5 × 10 mm cuvettes. Non-degassed, spectroscopic grade solvents and a 10 mm quartz cuvette were used. Absolute fluorescence quantum efficiencies of BODIPY derivatives were measured by absolute PL quantum yield spectrometer (Hamamatsu, C11347) in integrating sphere, using Equation (S1) given below[1]:

$$\Phi_F = \frac{N_{em}}{N_{abs}} = \frac{\alpha \int \frac{\lambda}{hc} I_{em}(\lambda)\, d\lambda}{\alpha \int \frac{\lambda}{hc} [I_{em}(\lambda) - I'_{ex}(\lambda)]\, d\lambda} \text{.....................(S1)}$$

where N_{em} and N_{abs} are the numbers of emitted and absorbed photons, respectively, α is the calibration factor for the measurement setup, λ is the wavelength, h is the Plank's constant, c is the speed of light, I_{em} (λ) is the emission intensity at λ, I_{ex} (λ) and I'_{ex} (λ) are the intensities of the excitation laser beam with λ in the absence and presence of the sample, respectively. The measured Φ_F value is independent of sharp and thickness of sample and power of excitation laser.

Electrochemical measurements. Cyclic voltammograms were obtained on a CH Instruments electrochemical workstation (CHI 610E, USA). Cyclic voltammograms of 1.0 mM BODIPYs were measured in their dichloromethane solution, containing 0.1 M tetrabutylammonium hexafluorophosphate ($TBAPF_6$) as the supporting electrolyte, glassy carbon electrode as a working electrode, Pt wire as a counter electrode, and saturated calomel electrode (SCE) as reference electrode at 100 mV s^{-1} of scanning rate at room temperature. Before each experiment, the working electrode was polished on a felt pad with 0.3 μm alumina (Buehler, Ltd., Lake Bluff, IL) and sonicated in Milli-Q deionized water and then in ethanol. The counter and reference electrodes were cleaned by rinsing and sonicating in water, and ethanol.

X-ray structure analysis. Crystals of compounds **3a** suitable for X-ray analysis were obtained via the slow diffusion of *n*-hexane into their dichloromethane solutions. The vial containing this solution was

placed and loosely capped to promote the crystallization. A suitable crystal was chosen and mounted on a glass fiber using grease. Data were collected using a Bruker APEX-II[2] CCD diffractometer operating at T = 293(2) K. The determination of unit cell parameters and data collections were performed with Mo *Kα* radiation (λ) at 0.71073 Å. The total number of runs and images was based on the strategy calculation from the program APEX2 (Bruker).[3] The structure was solved by the structure solution program Olex2 (Dolomanov et al., 2009),[4] and the model was refined with version 2014/7 of ShelXL (Sheldrick, 2015)[5] using full matrix least squares on F^2 minimization. CCDC 2553505 (**3a**) contain the supplementary crystallographic data for this paper. These data can be obtained free of charge from The Cambridge Crystallographic Data Centre via www.ccdc.cam.ac.uk/data_request/cif.

Electron paramagnetic resonance (EPR) experiments. CW X-band EPR spectra for radicals were acquired on Bruker EMX instrument. EPR spectra of **4d**/**4d** in the presence of cobaltocene ($CoCp_2$) in THF was recorded at room temperature.

2. Synthesis and characterization

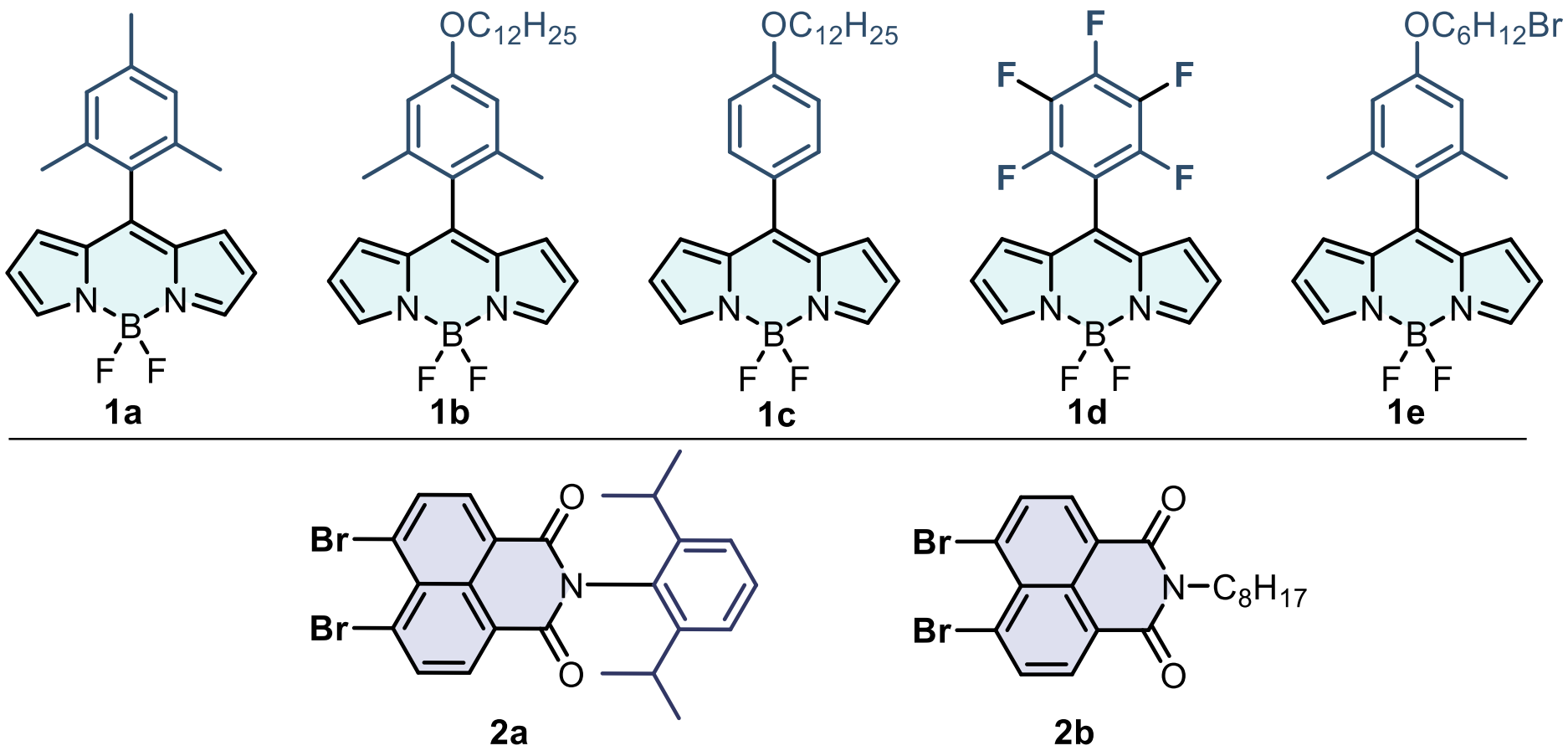

Figure S1. Chemical structure of BODIPYs **1a-1e** and naphthalimide (NMI) **2a-2b**

Those known BODIPYs **1a/1b/1c/1d** and NMI **2a/2b** were synthesized according to literatures.[6-11]

BDP-OH was synthesized according to the literature:[9]

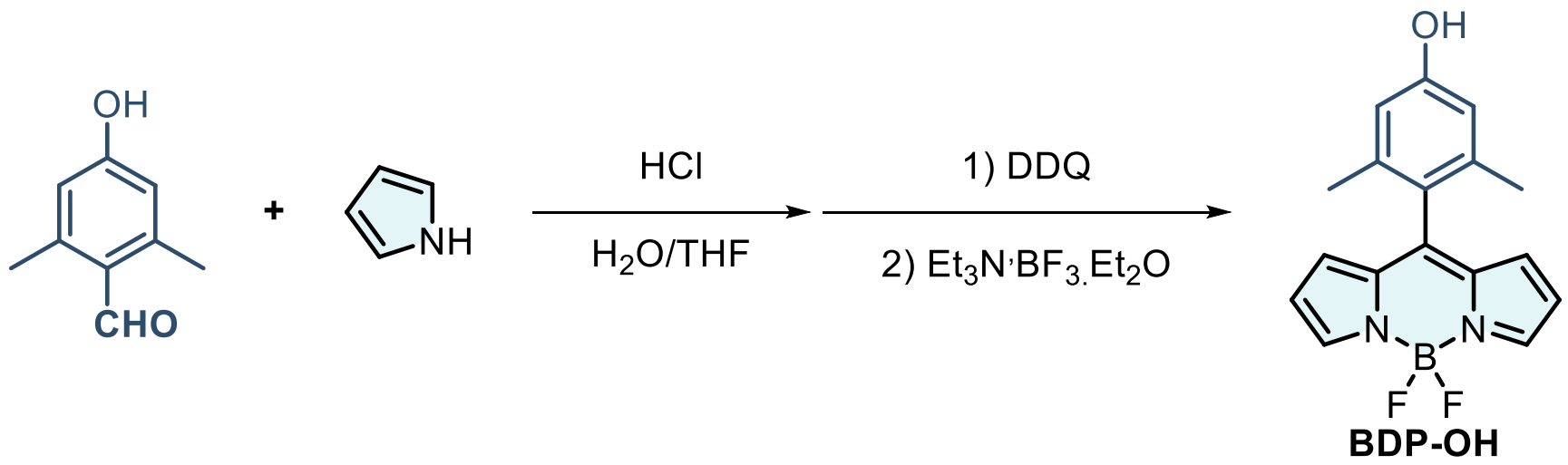

To 180 mL of aqueous HCl (2/180, v/v) was added dropwise a solution of 2,6-dimethyl-4-hydroxybenzaldehyde (3.0 g, 20 mmol) in THF (60 mL) at room temperature. Followed by the addition of the pyrrole (4.0 mL, 60 mmol). The reaction mixture was stirred at room temperature and the reaction progress was monitored by TLC. After 60 h, the THF was evaporated to dryness, resulting in a solid precipitate that often adhered to the flask walls. The aqueous phase was then removed, followed by thrice extractions using ethyl acetate (3 × 150 mL). The organic layer was collected, dried over anhydrous Na_2SO_4, and organic solvent was evaporated under vacuum. The crude product (3.2 g, 12 mmol), obtained from this step, was immediately utilized in the subsequent reaction. THF (80 mL) was employed as the solvent, to which DDQ (2.7 g, 12 mmol) was added. The reaction mixture was stirred at room temperature for 2 h. Subsequently, triethylamine (17 mL, 120 mmol) was added. The reaction mixture was stirred at room temperature for 1 h. Boron trifluoride ether (23 mL, 180 mmol) was slowly added under an ice water bath. The reaction mixture was then stirred at room temperature

for 8 h. Upon completion, the crude product was purified by column chromatography (silica; petroleum ether/DCM = 1:1, v/v) to give the red powder **BDP-OH** in 53% isolated yield (3.3 g).

Synthesis of BODIPY **1e:**

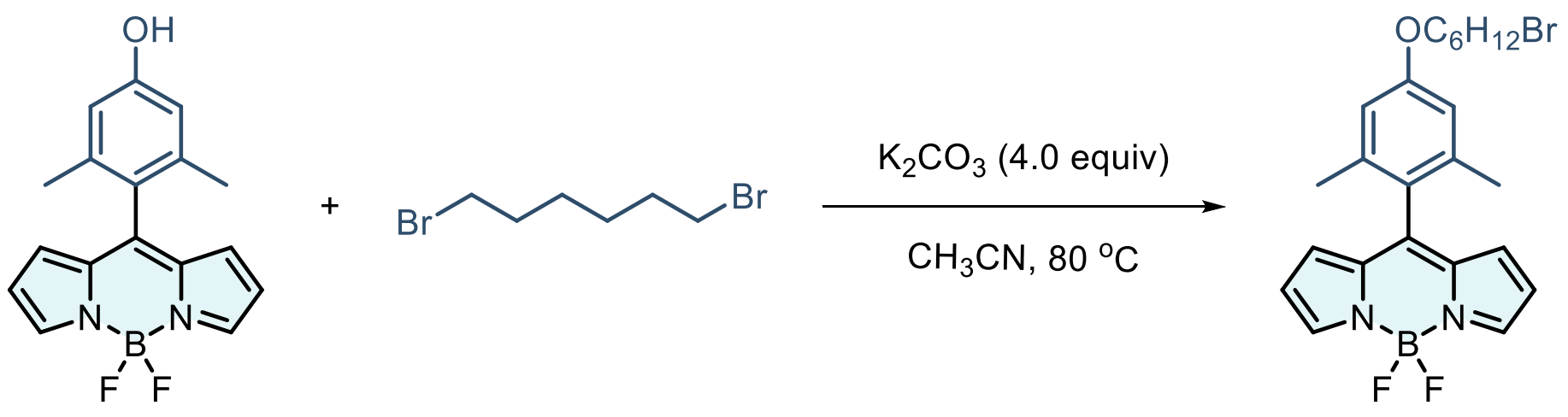

To a 100 mL round-bottom flask, **BDP-OH** (1.56 g, 5 mmol), K_2CO_3 (2.8 g, 20 mmol) and 1-bromododecane (2.3 mL, 15 mmol) were dissolved in acetonitrile (20.0 mL). The reaction mixture was stirred at 80 °C for 4 h. Upon completion, the cooled reaction mixture was transferred to a 1000 mL separatory funnel, then extracted thrice with dichloromethane (3 × 100 mL). The organic layer was collected, dried over anhydrous Na_2SO_4, and organic solvent was evaporated under vacuum. The crude product was purified by column chromatography (silica; petroleum ether/dichloromethane = 2:1, v/v) to give the powder **1** in 78% isolated yield (1.85 g). ^{1}H NMR (500 MHz, $CDCl_3$) δ 7.91 (s, 2H), 6.69 (d, *J* = 3.9 Hz, 2H), 6.67 (s, 2H), 6.47 (d, *J* = 3.4 Hz, 2H), 4.00 (t, *J* = 6.3 Hz, 2H), 3.45 (t, *J* = 6.7 Hz, 2H), 2.11 (s, 6H), 1.95 - 1.88 (m, 2H), 1.87 - 1.77 (m, 2H), 1.55 (s, 4H). ^{13}C NMR (126 MHz, $CDCl_3$) δ 159.3, 147.5, 144.2, 138.0, 135.7, 130.2, 124.9, 118.5, 113.3, 67.6, 33.8, 32.6, 29.1, 27.9, 25.3, 20.4 ppm. HRMS (ESI) m/z calcd for $C_{23}H_{26}BBrF_2N_2ONa^+$ $[M+Na]^+$ 497.1182, found 497.1187.

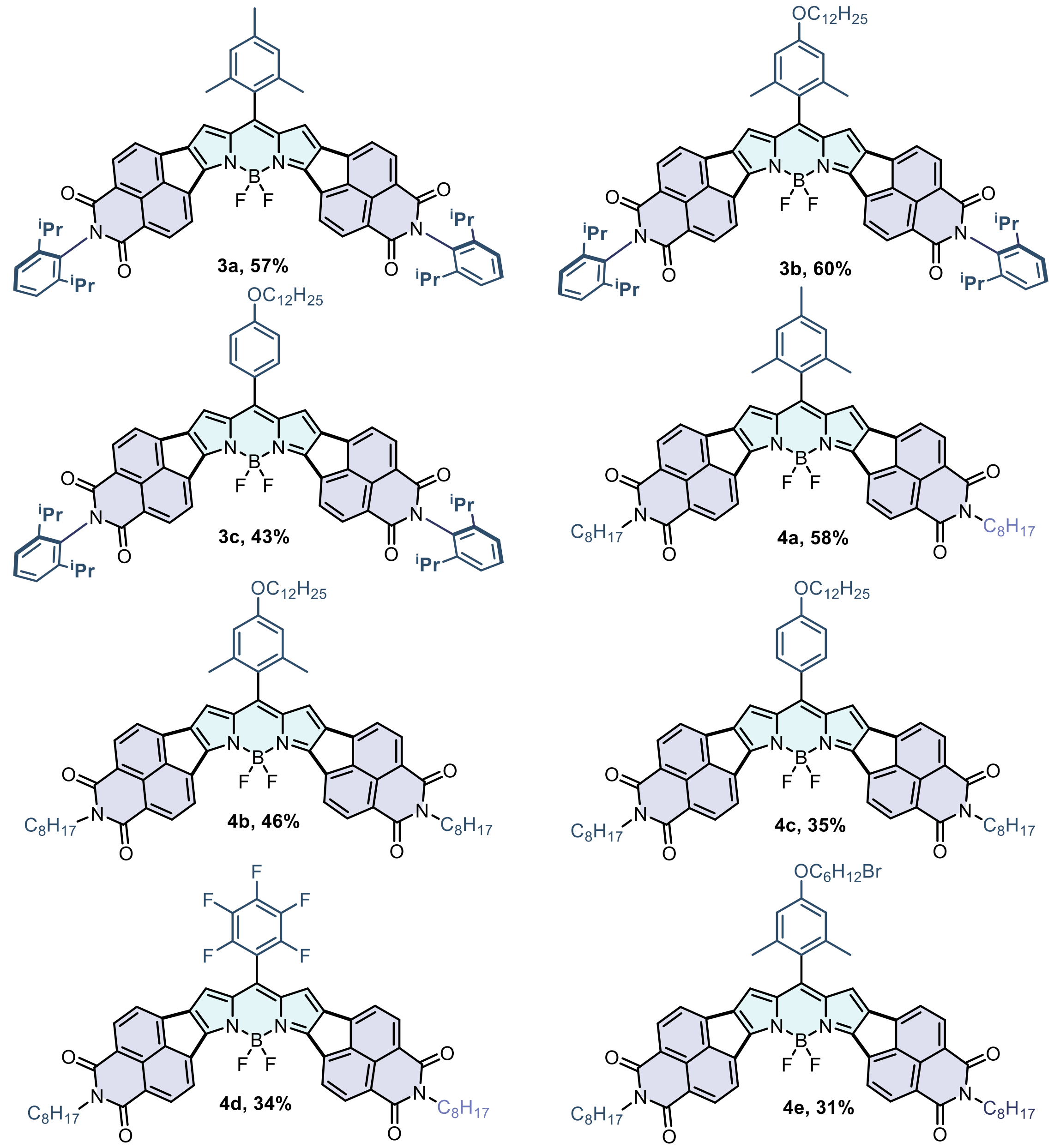

Figure S2. Chemical structure of BODIPYs **3a-3c/4a-4e**

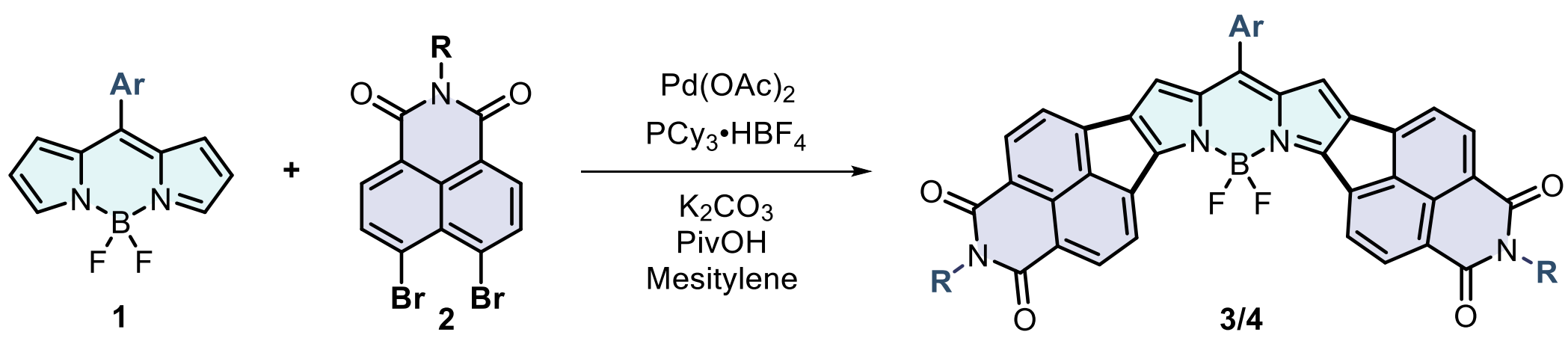

General Procedure for the Preparation of BODIPYs 3a-3c/4a-4e: To a 10 mL dry of Schlenk tube equipped with a magnetic stirring bar were added substrates BODIPY **1** (0.10 mmol), NMI **2** (0.40 mmol, 4 equiv), K_2CO_3 (55 mg, 0.40 mmol, 4 equiv), $Pd(OAc)_2$ (11 mg, 0.05 mmol, 0.5 equiv), $PCy_3{\cdot}HBF_4$ (37 mg, 0.10 mmol, 1 equiv) and PivOH (3 μL, 0.03 mmol, 0.3 equiv) under air. The

mixture was then evacuated and backfilled with argon three times. After that, dry mesitylene (3.0 mL) was added subsequently. After stirring at 130 °C under argon for 12 h. The reaction mixture was cooled to room temperature, extracted with DCM, and dried over anhydrous Na_2SO_4. The solvent was removed under reduced pressure, and the residue was purified by flash chromatography on silica gel with petroleum ether/DCM as the eluent to give the corresponding products **3/4**.

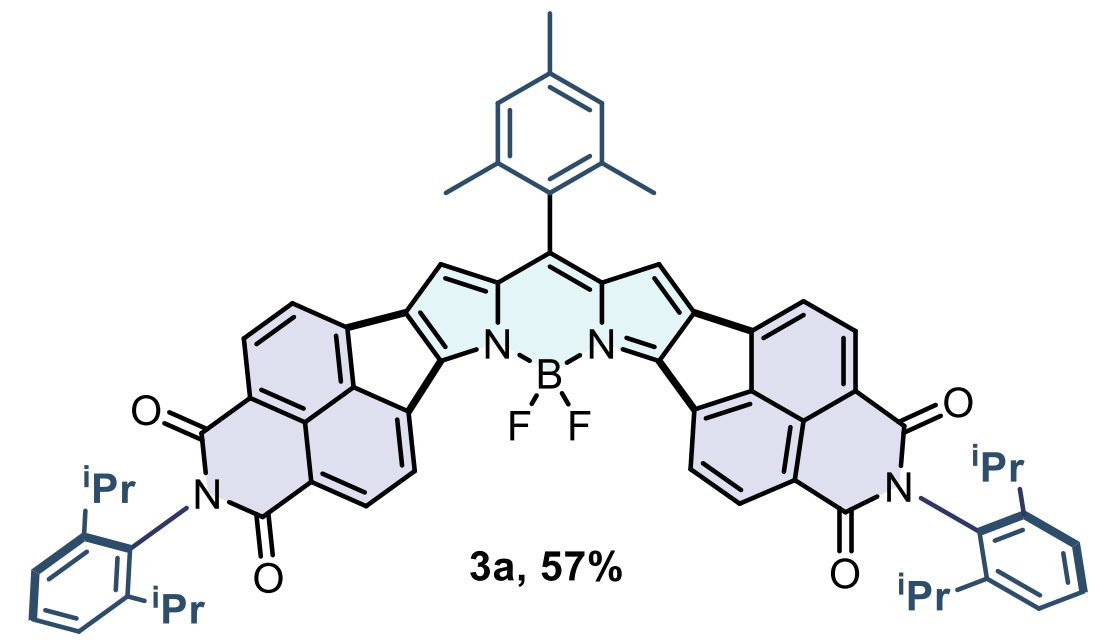

3a was prepared in 57% (58 mg) isolated yield as a black solid from **1a** (31 mg, 0.10 mmol), **2a** (205 mg, 0.40 mmol), K_2CO_3 (55 mg, 0.40 mmol), $Pd(OAc)_2$ (11 mg, 0.05 mmol), $PCy_3 \cdot HBF_4$ (37 mg, 0.10 mmol) and PivOH (3 μL, 0.03 mmol). ^1H NMR (400 MHz, $CDCl_3$) δ 8.67 (d, *J* = 7.5 Hz, 2H), 8.54 (d, *J* = 7.4 Hz, 2H), 8.48 (d, *J* = 7.3 Hz, 2H), 7.72 (d, *J* = 7.3 Hz, 2H), 7.49 (t, *J* = 7.8 Hz, 2H), 7.34 (d, *J* = 7.8 Hz, 4H), 7.09 (s, 2H), 6.79 (s, 2H), 2. 82 - 2.75 (m, 4H), 2.45 (s, 3H), 2.33 (s, 6H), 1.20 - 1.14 (m, 24H). ^{13}C NMR (101 MHz, $CDCl_3$) δ 163.5, 161.1, 145.8, 145.4, 142.4, 139.5, 137.6, 137.2, 136.7, 136.0, 133.1, 132.9, 132.1, 130.8, 129.6, 129.2, 128.6, 126.0, 125.6, 124.3, 124.0, 121.9, 121.8, 120.3, 29.1, 24.0, 21.3, 20.3 ppm. HRMS (APCI) m/z calcd for $C_{66}H_{56}O_4N_4BF_2^+$ $[M+H]^+$ 1017.4357, found 1017.4358.

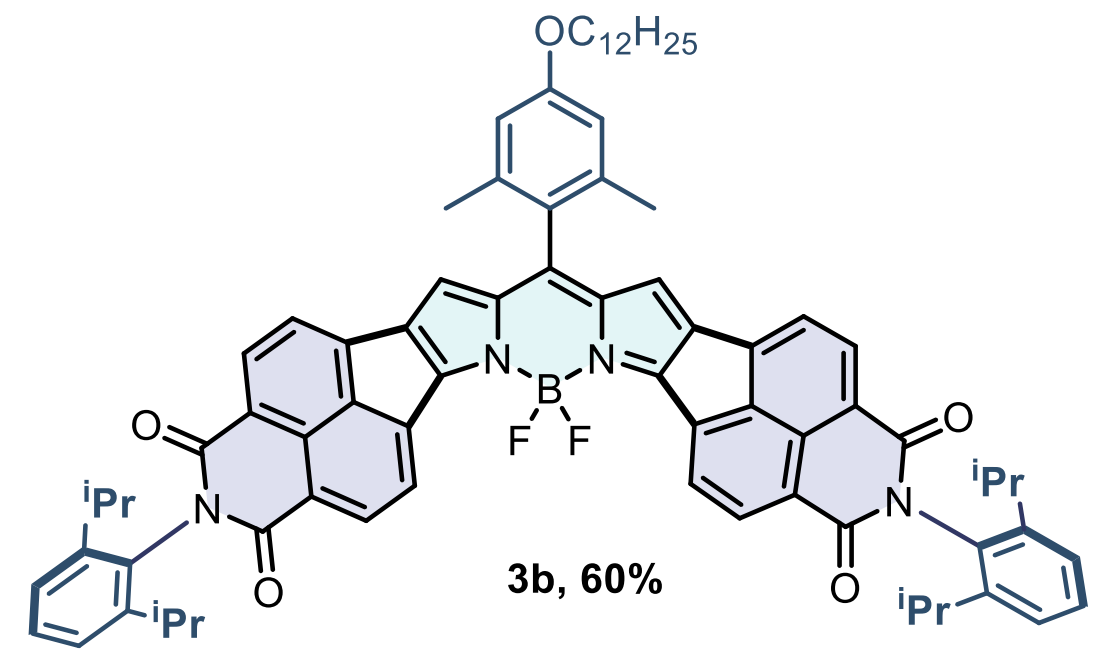

3b was prepared in 60% (71 mg) isolated yield as a black solid from **1b** (48 mg, 0.10 mmol), **2a** (205 mg, 0.40 mmol), K_2CO_3 (55 mg, 0.40 mmol), $Pd(OAc)_2$ (11 mg, 0.05 mmol), $PCy_3 \cdot HBF_4$ (37 mg, 0.10 mmol) and PivOH (3 μL, 0.03 mmol). ^1H NMR (400 MHz, $CDCl_3$) δ 8.67 (d, *J* = 7.4 Hz, 2H), 8.54 (d, *J* = 7.3 Hz, 2H), 8.49 (d, *J* = 7.3 Hz, 2H), 7.74 (d, *J* = 7.3 Hz, 2H), 7.48 (t, *J* = 7.8 Hz, 2H), 7.34

(d, J = 7.8 Hz, 4H), 6.81 - 6.80 (m, 4H), 4.09 - 4.03 (m, 2H), 2.82 - 2.75 (m, 4H), 2.33 (s, 6H), 1.87 (t, J = 7.3 Hz, 2H), 1.29 (s, 18H), 1.19 - 1.16 (m, 24H), 0.92 - 0.88 (m, 3H). ^{13}C NMR (101 MHz, $CDCl_3$) δ 163.6, 163.5, 161.9, 160.3, 145.8, 145.7, 141.9, 137.4, 137.0, 136.2, 133.1, 132.9, 132.5, 132.3, 130.8, 129.6, 126.0, 125.6, 124.2, 124.0, 121.8, 121.8, 114.9, 68.5, 31.9, 29.7, 29.6, 29.4, 29.2, 29.1, 26.1, 24.0, 22.7, 14.2 ppm. HRMS (APCI) m/z calcd for $C_{77}H_{78}O_5N_4BF_2^+$ $[M+H]^+$ 1187.6028, found 1187.6032.

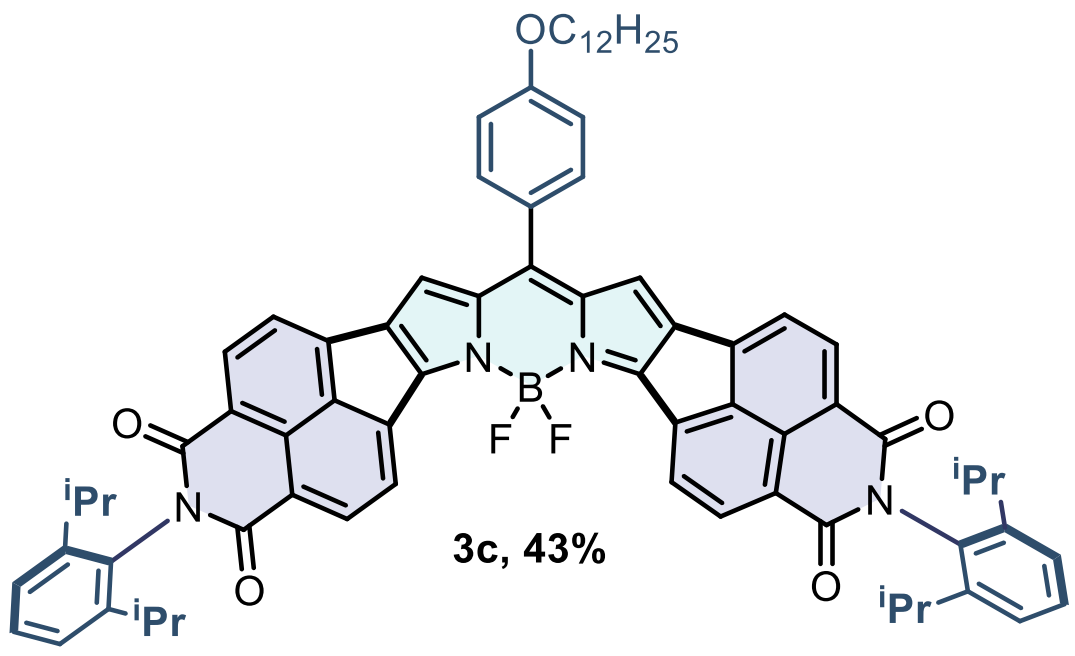

3c, 43%

3c was prepared in 43% (49 mg) isolated yield as a black solid from **1c** (45 mg, 0.10 mmol), **2a** (205 mg, 0.40 mmol), K_2CO_3 (55 mg, 0.40 mmol), $Pd(OAc)_2$ (11 mg, 0.05 mmol), $PCy_3 \cdot HBF_4$ (37 mg, 0.10 mmol) and PivOH (3 μL, 0.03 mmol). ^{1}H NMR (400 MHz, $CDCl_3$) δ 8.66 (d, J = 7.3 Hz, 2H), 8.54 (d, J = 7.4 Hz, 2H), 8.50 (d, J = 7.5 Hz, 2H), 7.78 (d, J = 7.4 Hz, 2H), 7.68 (d, J = 7.4 Hz, 2H), 7.51 - 7.47 (m, 2H), 7.34 (d, J = 7.8 Hz, 4H), 7.17 - 7.15 (m, 2H), 7.11 (s, 2H), 4.13 (t, J = 6.9 Hz, 2H), 2.83 - 2.75 (m, 4H), 1.93 - 1.87 (m, 2H), 1.29 (s, 18H), 1.18 (s, 24H), 0.89 (d, J = 7.0 Hz, 3H). ^{13}C NMR (101 MHz, $CDCl_3$) δ 163.6, 163.5, 161.9, 160.3, 145.7, 141.9, 137.4, 137.0, 136.2, 133.1, 132.9, 132.5, 132.3, 130.8, 129.6, 126.0, 125.6, 124.2, 124.0, 121.8, 121.8, 114.9, 68.5, 31.9, 29.7, 29.7, 29.6, 29.4, 29.2, 29.1, 26.1, 22.7, 14.2 ppm. HRMS (APCI) m/z calcd for $C_{75}H_{74}O_5N_4BF_2^+$ $[M+H]^+$ 1159.5715 , found 1159.5723.

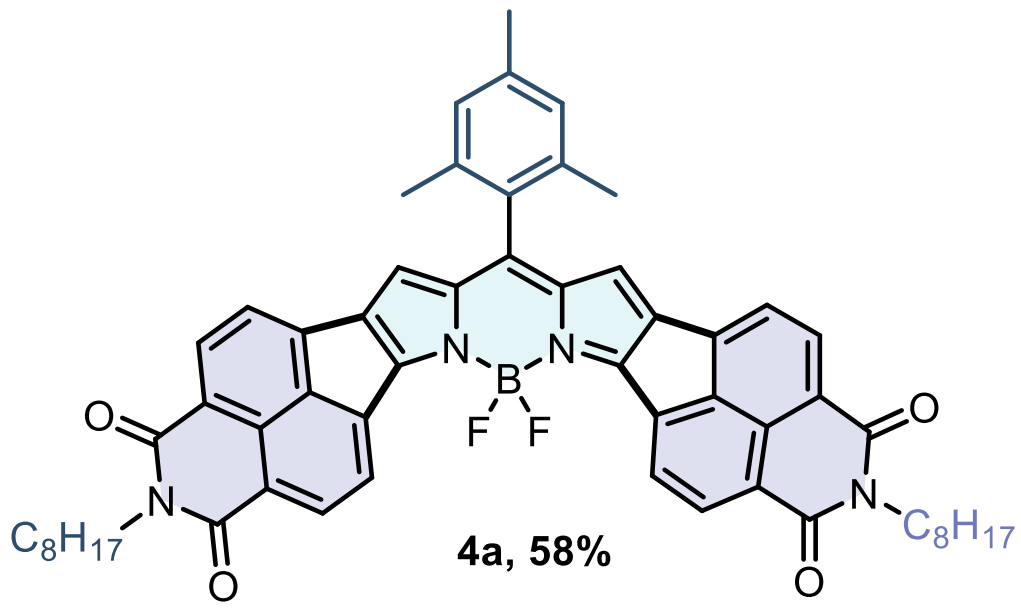

4a, 58%

4a was prepared in 58% (53 mg) isolated yield as a black solid from **1a** (31 mg, 0.10 mmol), **2b** (186 mg, 0.40 mmol), K_2CO_3 (55 mg, 0.40 mmol), $Pd(OAc)_2$ (11 mg, 0.05 mmol), $PCy_3 \cdot HBF_4$ (37 mg, 0.10

mmol) and PivOH (3 μL, 0.03 mmol). ^{1}H NMR (400 MHz, CDCl$_3$) δ 8.48 (d, *J* = 7.4 Hz, 2H), 8.35 (d, *J* = 7.3 Hz, 2H), 8.30 (d, *J* = 7.3 Hz, 2H), 7.55 (d, *J* = 7.1 Hz, 2H), 7.09 (s, 2H), 6.70 (s, 2H), 4.15 (t, *J* = 7.6 Hz, 4H), 2.45 (s, 3H), 2.33 (s, 6H), 1.78 - 1.70 (m, 4H), 1.29 - 1.25 (m, 20H), 0.89 (d, *J* = 6.9 Hz, 6H). ^{13}C NMR (101 MHz, CDCl$_3$) δ 163.4, 163.3, 160.9, 145.2, 142.3, 139.5, 137.3, 137.1, 136.6, 135.6, 132.3, 132.1, 131.6, 129.2, 128.6, 125.1, 125.1, 124.4, 121.8, 121.6, 120.0, 40.6, 31.8, 29.4, 29.3, 28.4, 27.2, 22.7, 21.2, 20.2, 14.1 ppm. HRMS (APCI) m/z calcd for $C_{58}H_{56}O_4N_4BF_2^+$ $[M+H]^+$ 921.4357, found 921.4360.

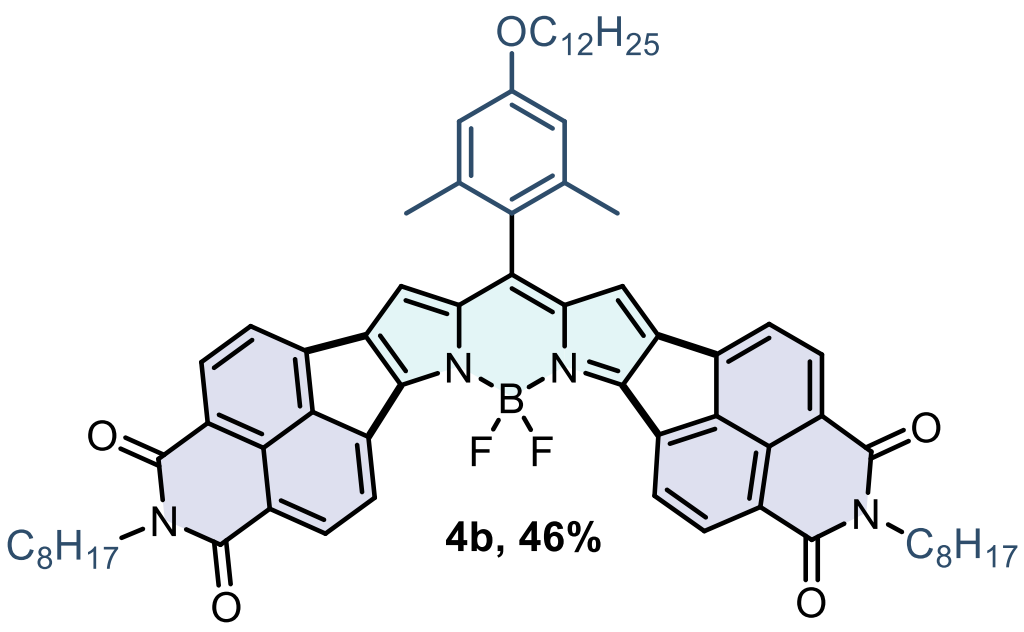

4b, 46%

4b was prepared in 46% (50 mg) isolated yield as a black solid from **1b** (48 mg, 0.10 mmol), **2b** (186 mg, 0.40 mmol), K_2CO_3 (55 mg, 0.40 mmol), $Pd(OAc)_2$ (11 mg, 0.05 mmol), $PCy_3{\cdot}HBF_4$ (37 mg, 0.10 mmol) and PivOH (3 μL, 0.03 mmol). ^{1}H NMR (400 MHz, CDCl$_3$) δ 8.54 (d, *J* = 7.4 Hz, 2H), 8.41 (d, *J* = 7.3 Hz, 2H), 8.36 (d, *J* = 7.3 Hz, 2H), 7.62 (d, *J* = 7.3 Hz, 2H), 6.79 (s, 2H), 6.73 (s, 2H), 4.16 (t, *J* = 7.8 Hz, 4H), 4.06 (t, *J* = 6.5 Hz, 2H), 2.31 (s, 6H), 1.90 - 1.83 (m, 2H), 1.77 - 1.70 (m, 4H), 1.44 - 1.41 (m, 10H), 1.29 (s, 28H), 0.88 (t, *J* = 6.7 Hz, 9H). ^{13}C NMR (101 MHz, CDCl$_3$) δ 163.5, 163.4, 161.0, 159.9, 145.1, 142.6, 138.4, 137.4, 137.2, 135.7, 132.4, 132.2, 131.7, 125.3, 124.4, 124.2, 121.9, 121.8, 120.1, 113.7, 68.1, 40.6, 32.0, 31.8, 29.7, 29.7, 29.7, 29.6, 29.5, 29.4, 29.3, 28.4, 27.2, 26.1, 22.7, 20.6, 14.2, 14.1 ppm. HRMS (APCI) m/z calcd for $C_{69}H_{78}O_5N_4BF_2^+$ $[M+H]^+$ 1091.6028, found 1091.6037.

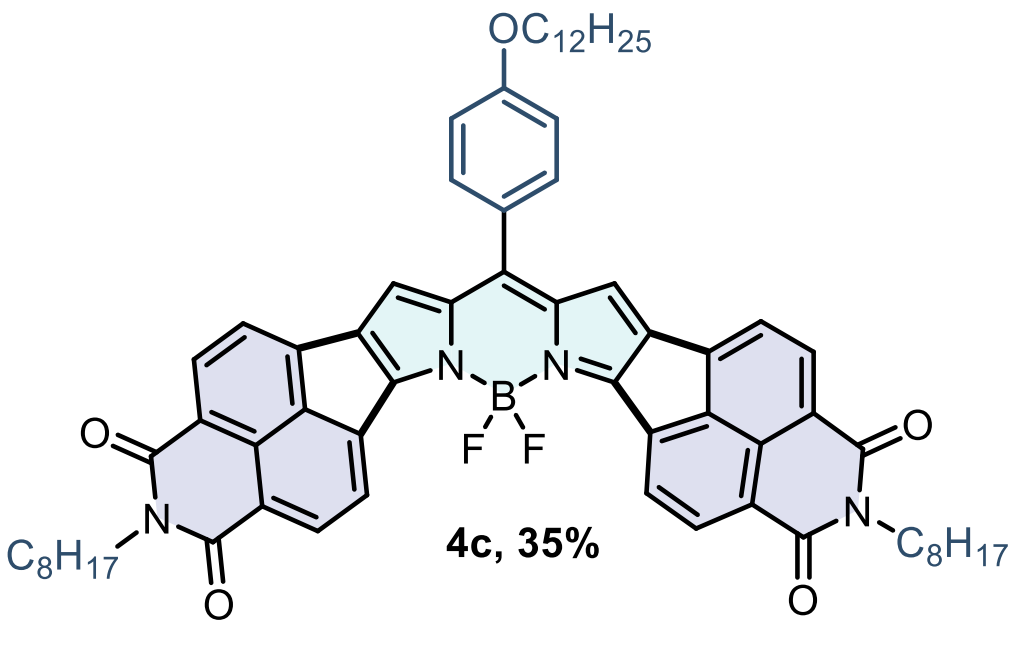

4c, 35%

4c was prepared in 35% (37 mg) isolated yield as a black solid from **1c** (45 mg, 0.10 mmol), **2b** (186

mg, 0.40 mmol), K_2CO_3 (55 mg, 0.40 mmol), $Pd(OAc)_2$ (11 mg, 0.05 mmol), $PCy_3{\cdot}HBF_4$ (37 mg, 0.10 mmol) and PivOH (3 μL, 0.03 mmol). ^{1}H NMR (400 MHz, $CDCl_3$) δ 8.45 (d, J = 7.4 Hz, 2H), 8.29 - 8.26 (m, 4H), 7.61 (d, J = 8.6 Hz, 2H), 7.56 (d, J = 7.3 Hz, 2H), 7.12 (d, J = 8.6 Hz, 2H), 6.93 (s, 2H), 4.13 - 4.07 (m, 6H), 1.88 (t, J = 7.3 Hz, 2H), 1.73 - 1.67 (m, 4H), 1.30 (s, 38H), 0.89 (d, J = 7.1 Hz, 9H). ^{13}C NMR (126 MHz, $CDCl_3$) δ 167.8, 163.4, 161.8, 160.1, 141.7, 137.6, 136.9, 135.8, 132.4, 132.3, 132.1, 131.8, 130.9, 128.8, 125.6, 125.2, 124.2, 121.8, 121.7, 121.4, 114.9, 68.5, 68.2, 40.6, 38.7, 31.9, 30.3, 29.7, 29.4, 29.3, 28.9, 28.4, 27.2, 26.1, 23.7, 22.9, 22.6, 14.1, 10.9 ppm. HRMS (APCI) m/z calcd for $C_{67}H_{74}O_5N_4BF_2^+$ $[M+H]^+$ 1063.5715 , found 1063.5729.

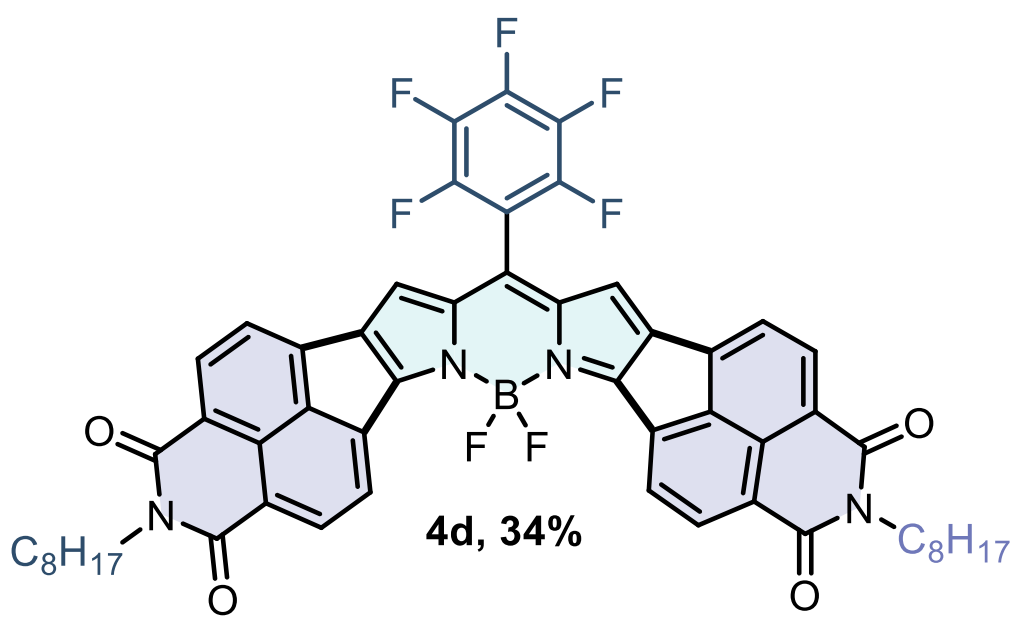

4d was prepared in 34% (32 mg) isolated yield as a black solid from **1d** (36 mg, 0.10 mmol), **2b** (186 mg, 0.40 mmol), K_2CO_3 (55 mg, 0.40 mmol), $Pd(OAc)_2$ (11 mg, 0.05 mmol), $PCy_3{\cdot}HBF_4$ (37 mg, 0.10 mmol) and PivOH (3 μL, 0.03 mmol). ^{1}H NMR (400 MHz, $CDCl_3$) δ 8.54 (d, J = 7.4 Hz, 2H), 8.41 - 8.35 (m, 4H), 7.66 (d, J = 7.3 Hz, 2H), 6.80 (s, 2H), 4.15 (t, J = 7.6 Hz, 4H), 1.77 - 1.70 (m, 4H), 1.44 - 1.35 (m, 8H), 1.31 - 1.29 (m, 12H), 0.90 - 0.86 (m, 6H). ^{13}C NMR (101 MHz, $CDCl_3$) δ 163.3, 163.1, 162.6, 141.7, 138.0, 137.9, 134.7, 132.4, 132.2, 130.9, 125.8, 125.2, 124.9, 122.2, 122.1, 119.4, 40.7, 31.8, 29.4, 29.3, 28.4, 27.2, 22.7, 14.1 ppm. HRMS (APCI) m/z calcd for $C_{55}H_{45}BF_7N_4O_4^+$ $[M+H]^+$ 969.3417, found 969.3413.

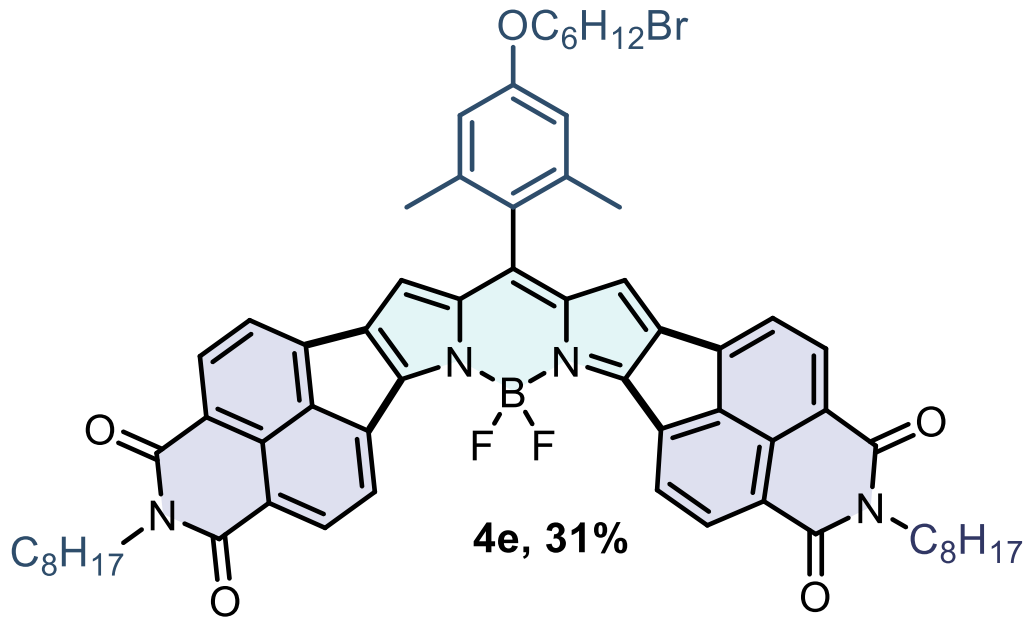

4e was prepared in 31% (32 mg) isolated yield as a black solid from **1e** (47 mg, 0.10 mmol), **2b** (186 mg, 0.40 mmol), K_2CO_3 (55 mg, 0.40 mmol), $Pd(OAc)_2$ (11 mg, 0.05 mmol), $PCy_3{\cdot}HBF_4$ (37 mg, 0.10

mmol) and PivOH (3 μL, 0.03 mmol). ^{1}H NMR (400 MHz, $CDCl_3$) δ 8.55 (d, *J* = 7.4 Hz, 2H), 8.43 (d, *J* = 7.3 Hz, 2H), 8.38 (d, *J* = 7.3 Hz, 2H), 7.63 (d, *J* = 7.3 Hz, 2H), 6.79 (s, 2H), 6.73 (s, 2H), 4.16 (t, *J* = 7.6 Hz, 4H), 4.07 (t, *J* = 6.2 Hz, 2H), 3.48 (t, *J* = 6.8 Hz, 2H), 2.30 (s, 6H), 1.96 (t, *J* = 6.8 Hz, 2H), 1.92 - 1.86 (m, 2H), 1.76 - 1.70 (m, 4H), 1.61 - 1.57 (m, 4H), 1.44 - 1.26 (m, 20H), 0.90 - 0.86 (m, 6H). ^{13}C NMR (101 MHz, $CDCl_3$) δ 163.5, 163.4, 161.0, 159.7, 145.0, 142.6, 138.4, 137.4, 135.7, 132.5, 132.2, 131.7, 125.3, 124.4, 124.3, 121.9, 121.8, 120.1, 113.7, 67.8, 40.6, 33.9, 32.7, 31.8, 29.2, 29.1, 28.4, 27.9, 27.2, 25.4, 22.7, 20.6, 14.1 ppm. HRMS (APCI) m/z calcd for $C_{63}H_{64}BBrF_2N_4O_5^+$ $[M+H]^+$ 1085.4194, found 1085.4191.

3. Crystal data

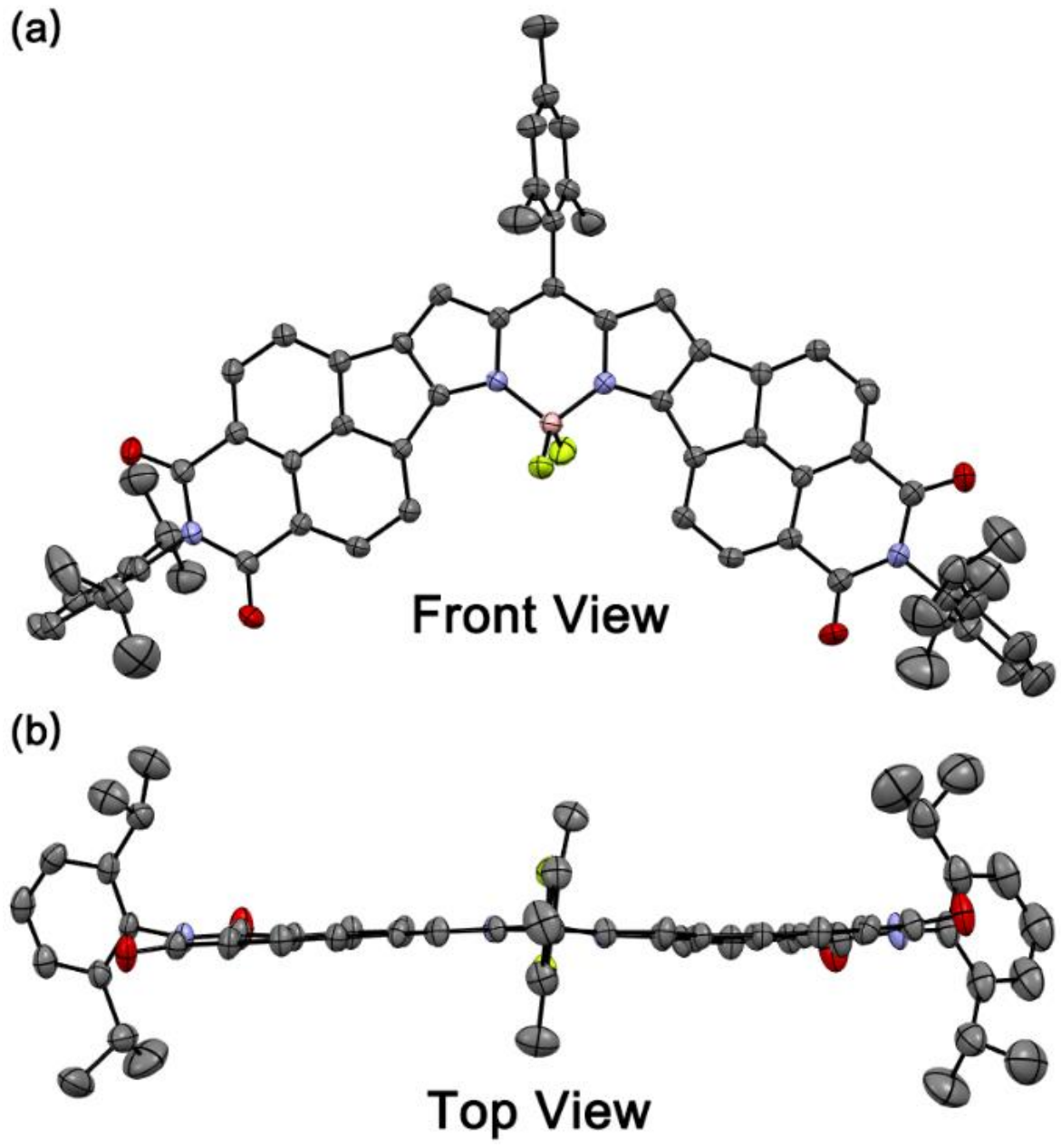

Figure S3. Top and front views of X-Ray structures of BODIPY **3a**, C, gray; N, blue; B, pink; F, green; O, red. Hydrogen atoms were removed for clarity.

Table S1. Selected geometrical parameters of compound **3a** obtained from crystallography

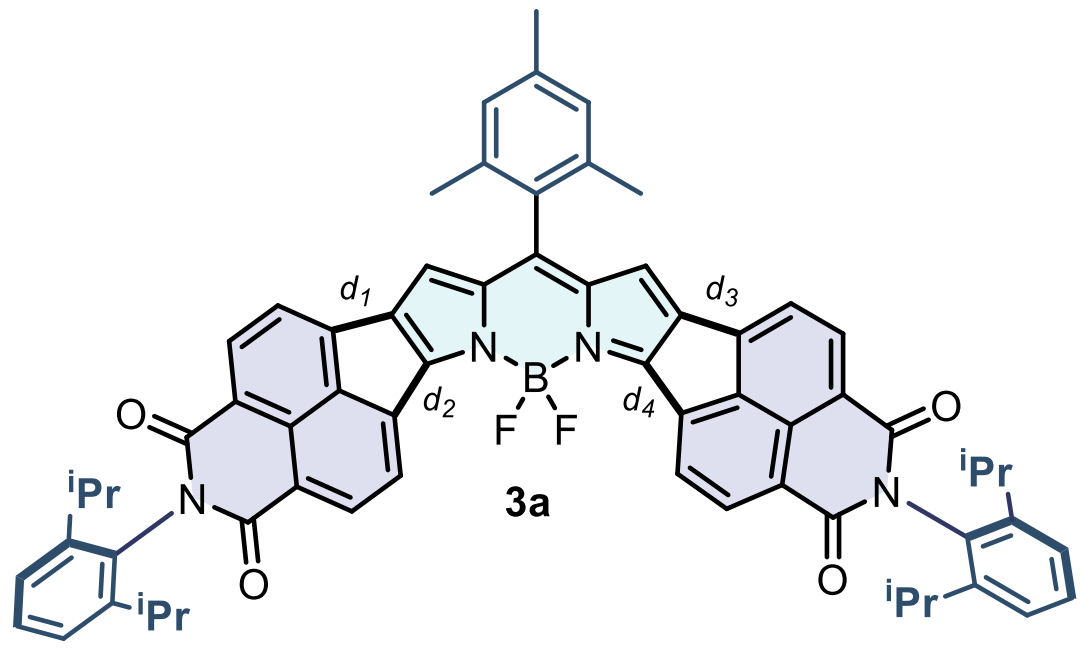

3a

The **B-N** bond distances (Å)	1.541 (6)<br>1.537 (6)
Dihedral angle of two pyrrole rings (deg)	2.550 (19)
Dihedral angle between NMI and the BODIPY core (deg)	4.193 (75)<br>3.302 (61)
The **C-C** bond（$\boldsymbol{d_1/d_2/d_3/d_4}$） distances (Å)	1.469 (6)<br>1.466 (6)<br>1.448 (5)<br>1.456 (6)
Dihedral angles between *meso*-mesityl ring and dipyyrin core (deg)	82.300 (126)

Table S2. Crystal data and structure refinement for **3a**.

Dyes	**3a**
Empirical formula	$C_{66}H_{55}BF_2N_4O_4$
Formula weight	1016.95
Temperature/K	293.15
Crystal system	triclinic
Space group	P-1
a/Å	14.969(4)
b/Å	15.677(4)
c/Å	15.843(4)
α/°	109.961(4)
β/°	105.784(4)
γ/°	106.582(4)
Volume/Å^3	3054.6(13)
Z	2
ρ_{calc}g/cm^3	1.106
μ/mm^{-1}	0.073
F(000)	1068.0
Crystal size/mm^3	0.22 × 0.21 × 0.20
Radiation	MoKα (λ = 0.71073)
2Θ range for data collection/°	3.112 to 49.998
Index ranges	-17≤h≤17, -18≤k≤18, -18≤l≤18
Reflections collected	22072
Independent reflections	10548 [Rint = 0.0994, Rsigma = 0.1831]
Data/restraints/parameters	10548/1/706
Goodness-of-fit on F^2	0.885
Final R indexes [I>=2σ (I)]	R_1 = 0.0759, wR_2 = 0.1664
Final R indexes [all data]	R_1 = 0.2078, wR_2 = 0.2227
Largest diff. peak/hole / e Å^3	0.22/-0.23

4. Photophysical data

Table S3. Spectroscopic and photophysical properties of BODIPYs **3a-3c** and **4a-4e** in different solvents.

Dyes	Solvents	λ_{abs}^{max} (nm)	$\varepsilon_{abs}^{max\ a}$	λ_{em}^{max} (nm)	Φ^{b}	Stokes shift (cm^{-1})
**3a**	Hexane	666	165800	671	0.56	110
	Toluene	676	120200	687	0.39	240
	CH_2Cl_2	678	116700	692	0.10	300
	THF	672	129300	686	0.12	300
**3b**	Hexane	666	183400	671	0.46	110
	Toluene	678	138300	687	0.35	190
	CH_2Cl_2	678	123200	693	0.13	320
	THF	672	120700	686	0.11	300
**3c**	Hexane	662	170800	666	0.53	90
	Toluene	672	104200	682	0.41	220
	CH_2Cl_2	672	94200	687	0.12	330
	THF	672	120700	681	0.11	200
**4a**	Hexane	666	189400	668	0.54	90
	Toluene	676	184900	687	0.43	240
	CH_2Cl_2	676	127200	691	0.11	320
	THF	672	171300	684	0.14	260
**4b**	Hexane	664	191400	671	0.55	160
	Toluene	676	169300	686	0.40	220
	CH_2Cl_2	678	150300	691	0.14	280
	THF	672	184900	685	0.10	280
**4c**	Toluene	672	159800	682	0.42	220
	CH_2Cl_2	672	114700	685	0.12	280
	THF	666	106700	685	0.10	270
**4d**	Hexane	682	155300	692	0.56	210
	Toluene	692	184900	708	0.37	330
	CH_2Cl_2	692	148300	706	0.15	290
	THF	686	172800	702	0.12	330
**4e**	Hexane	666	159800	670	0.55	90
	Toluene	676	126200	686	0.41	220

CH_2Cl_2	677	104700	692	0.11	320
THF	671	119200	686	0.10	330

[a]Corresponding to the strongest absorption maximum, the unit for ε is M^{-1} cm^{-1}. [b]Fluorescence quantum yields were measured by absolute PL quantum yield spectrometer (Hamamatsu) using integrating sphere. The standard errors are less than 5%.

UV-vis and fluorescence spectroscopies

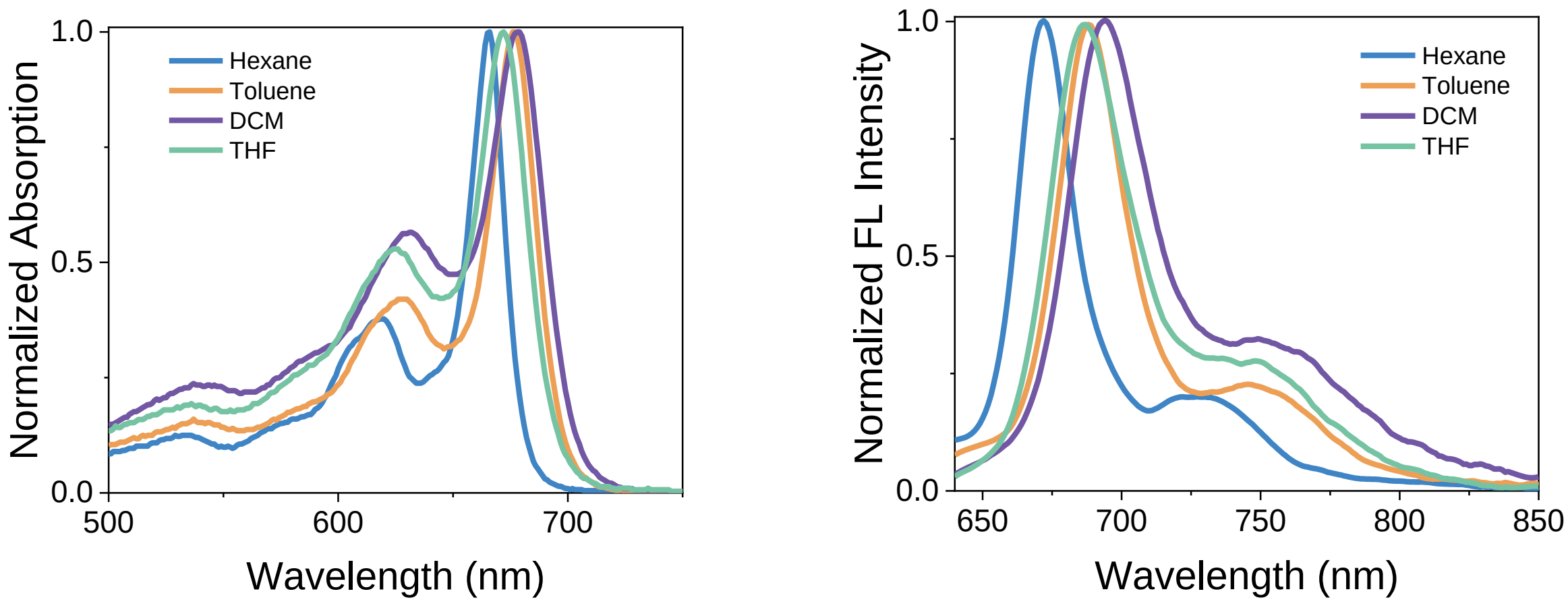

Figure S4. Absorption (left) and emission (right) spectra of compound **3a** (2 μM) recorded in different solvents. Excited at 630 nm.

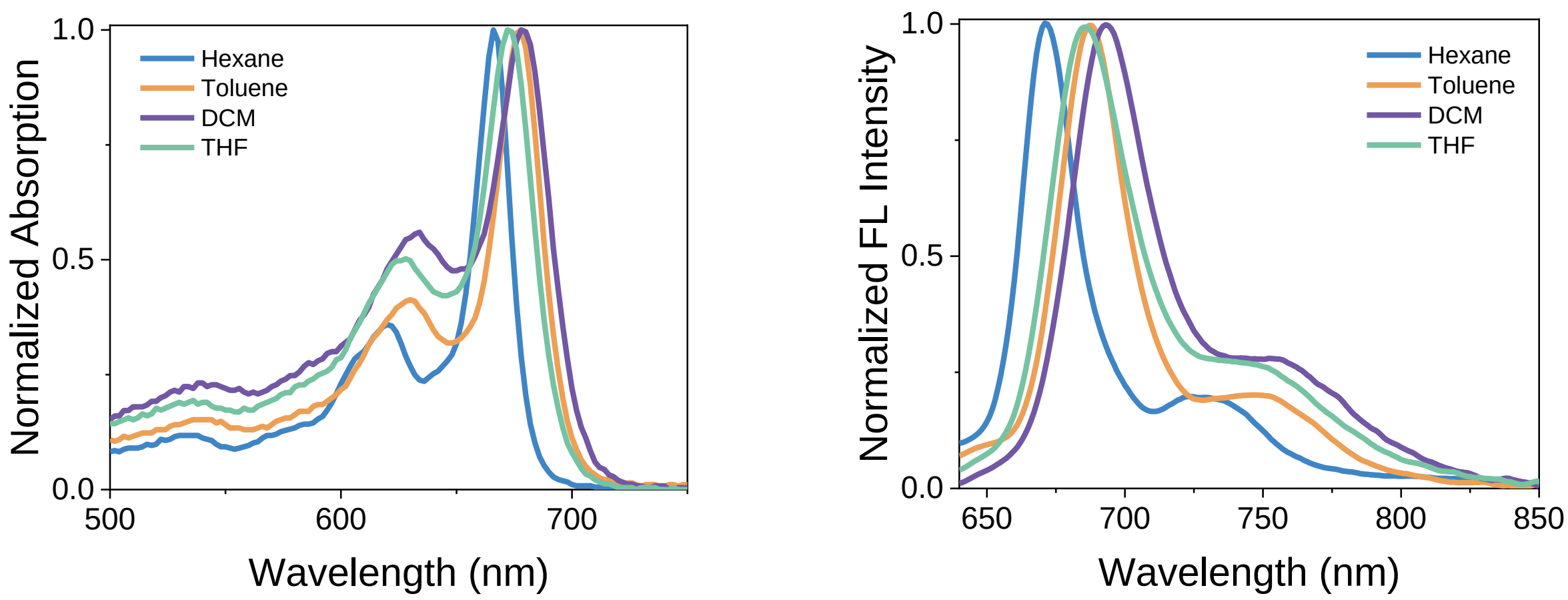

Figure S5. Absorption (left) and emission (right) spectra of compound **3b** (2 μM) recorded in different solvents. Excited at 630 nm.

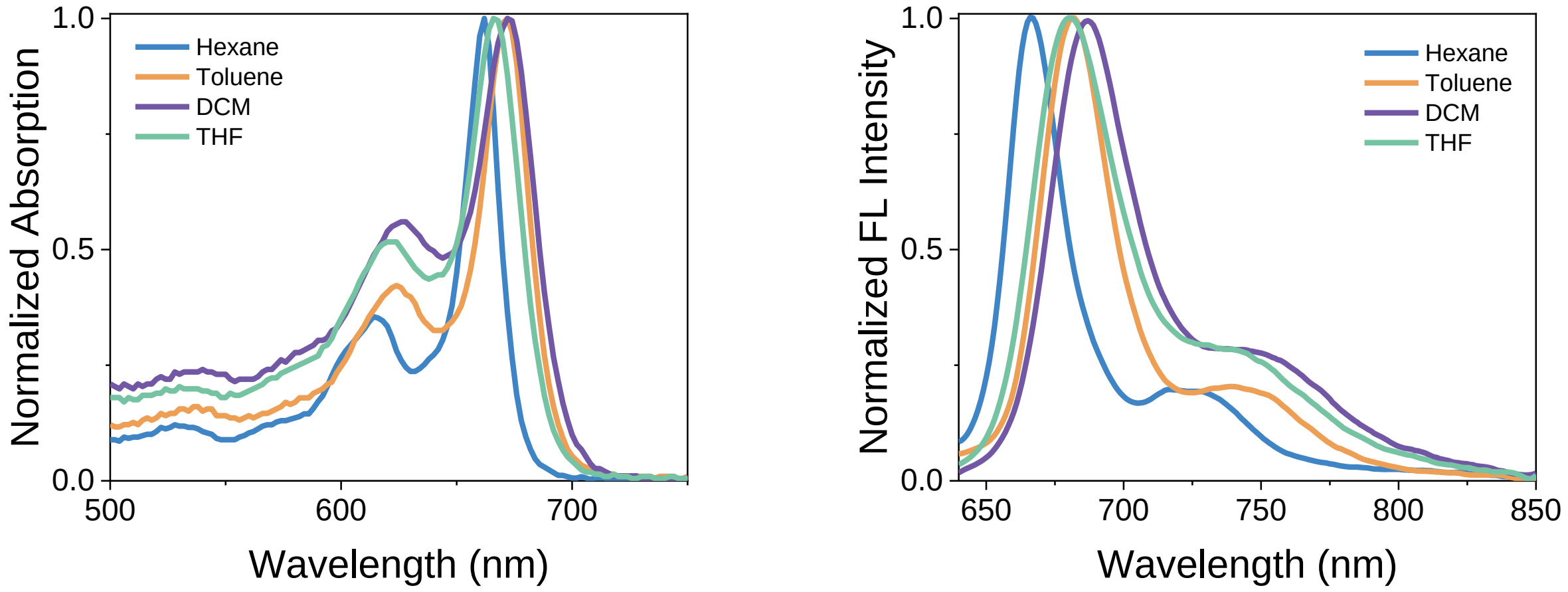

Figure S6. Absorption (left) and emission (right) spectra of compound **3c** (2 μM) recorded in different solvents. Excited at 630 nm.

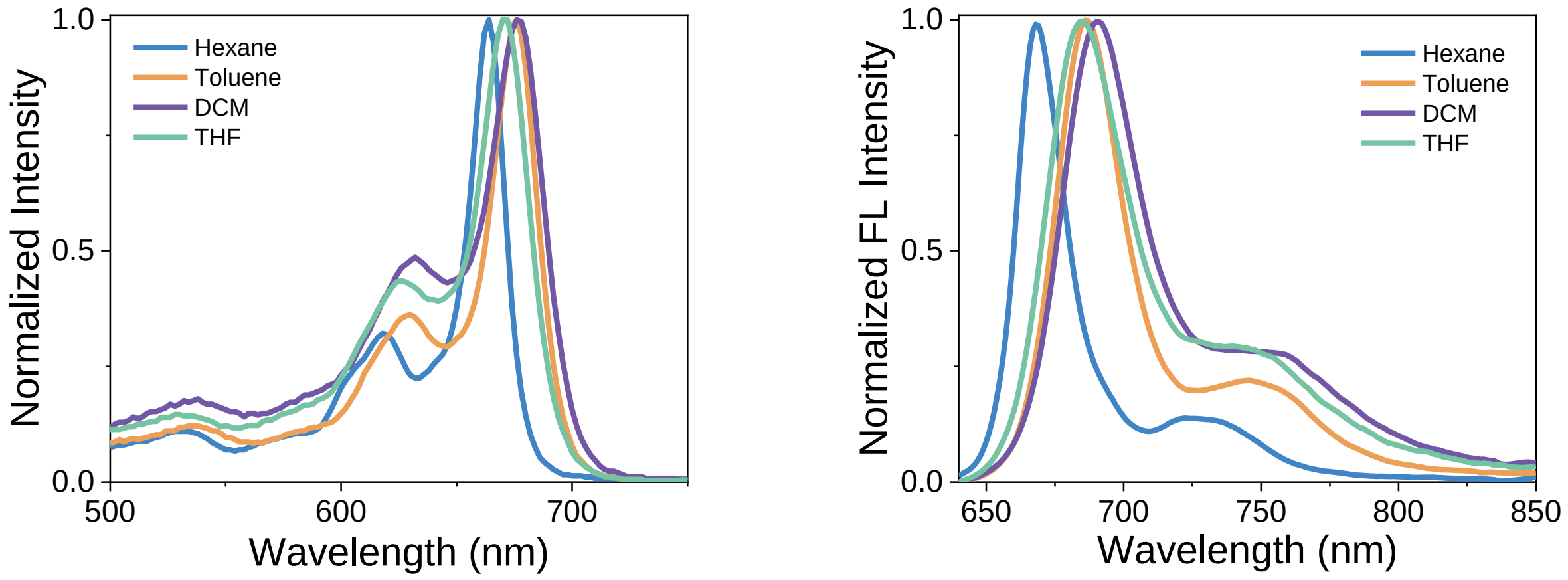

Figure S7. Absorption (left) and emission (right) spectra of compound **4a** (2 μM) recorded in different solvents. Excited at 630 nm.

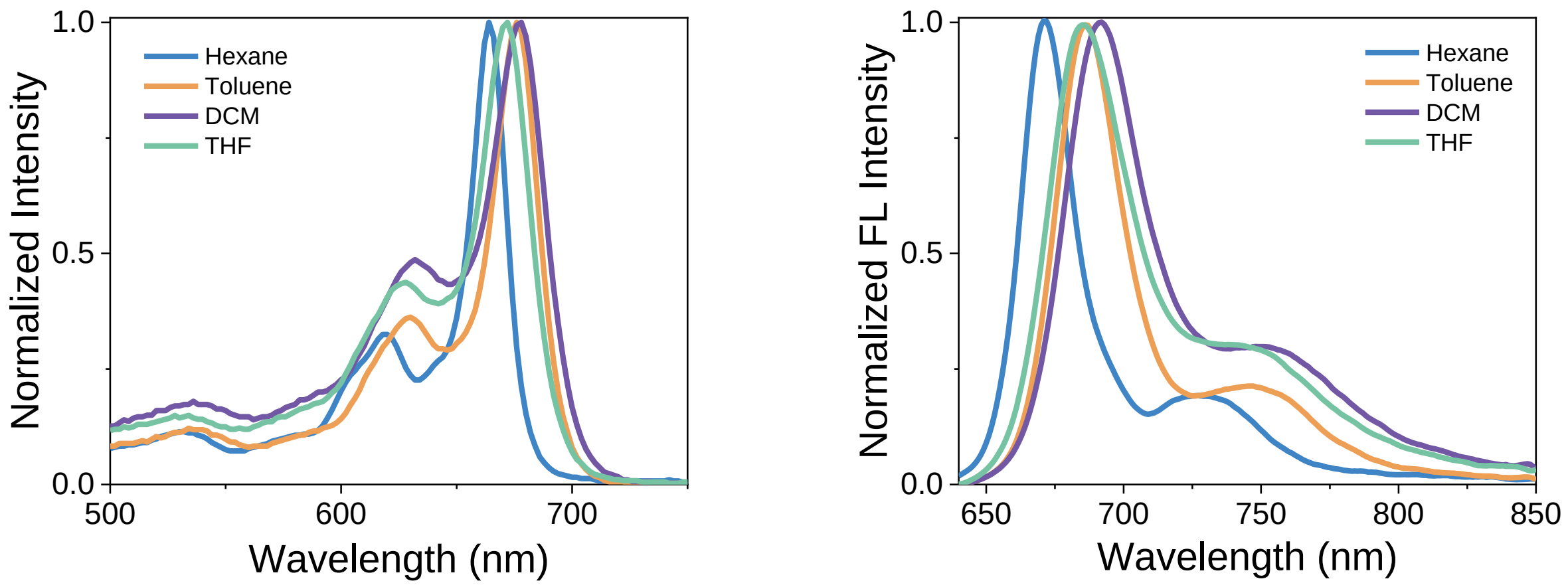

Figure S8. Absorption (left) and emission (right) spectra of compound **4b** (2 μM) recorded in different solvents. Excited at 630 nm.

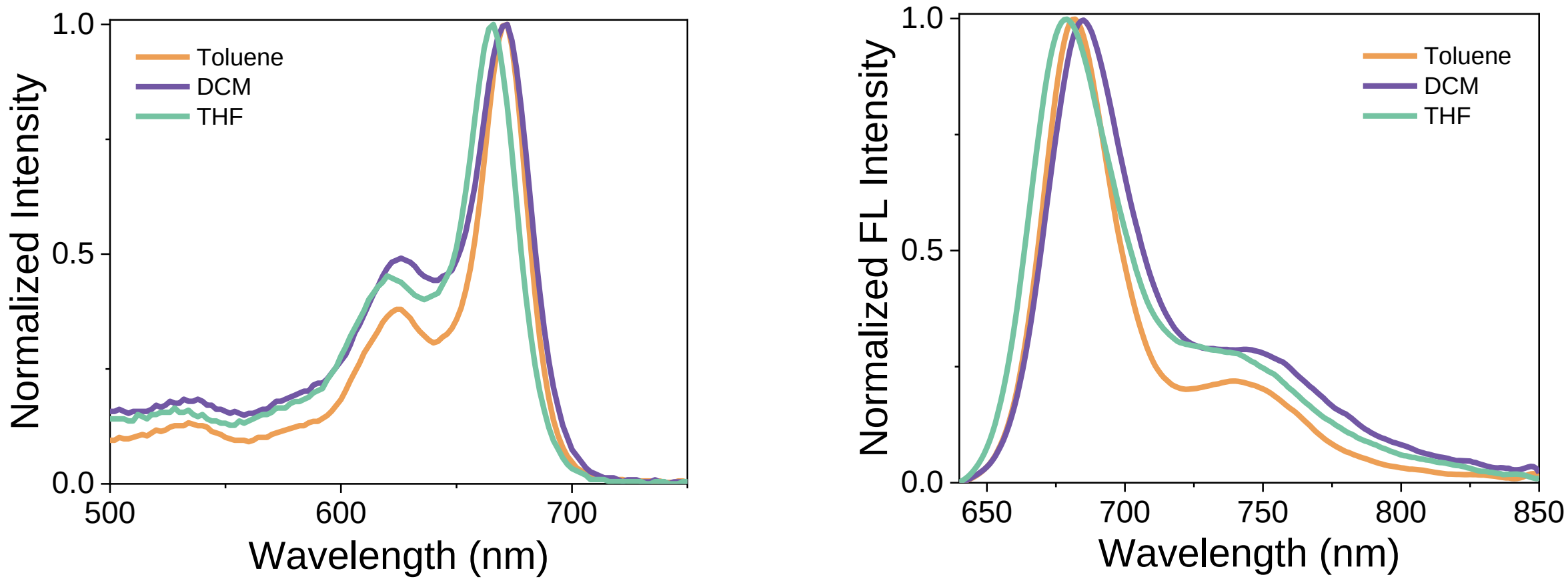

Figure S9. Absorption (left) and emission (right) spectra of compound **4c** (2 μM) recorded in different solvents. Excited at 630 nm.

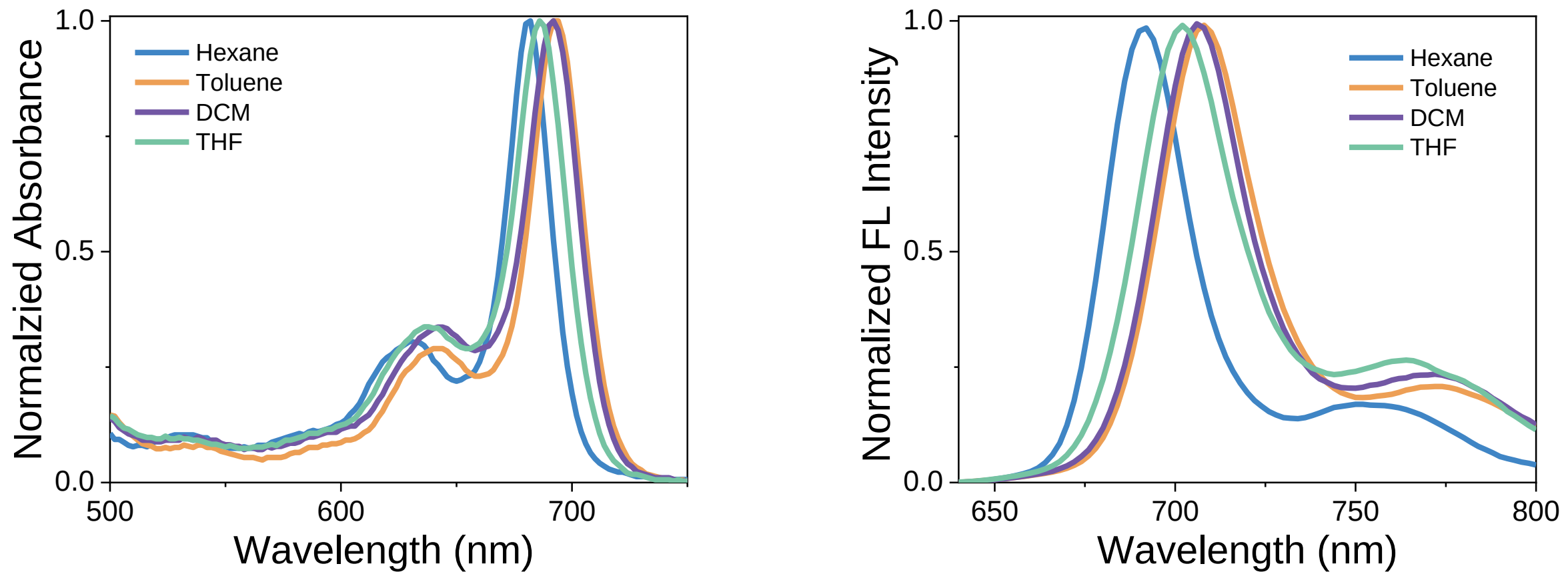

Figure S10. Absorption (left) and emission (right) spectra of compound **4d** (2 μM) recorded in different solvents. Excited at 630 nm.

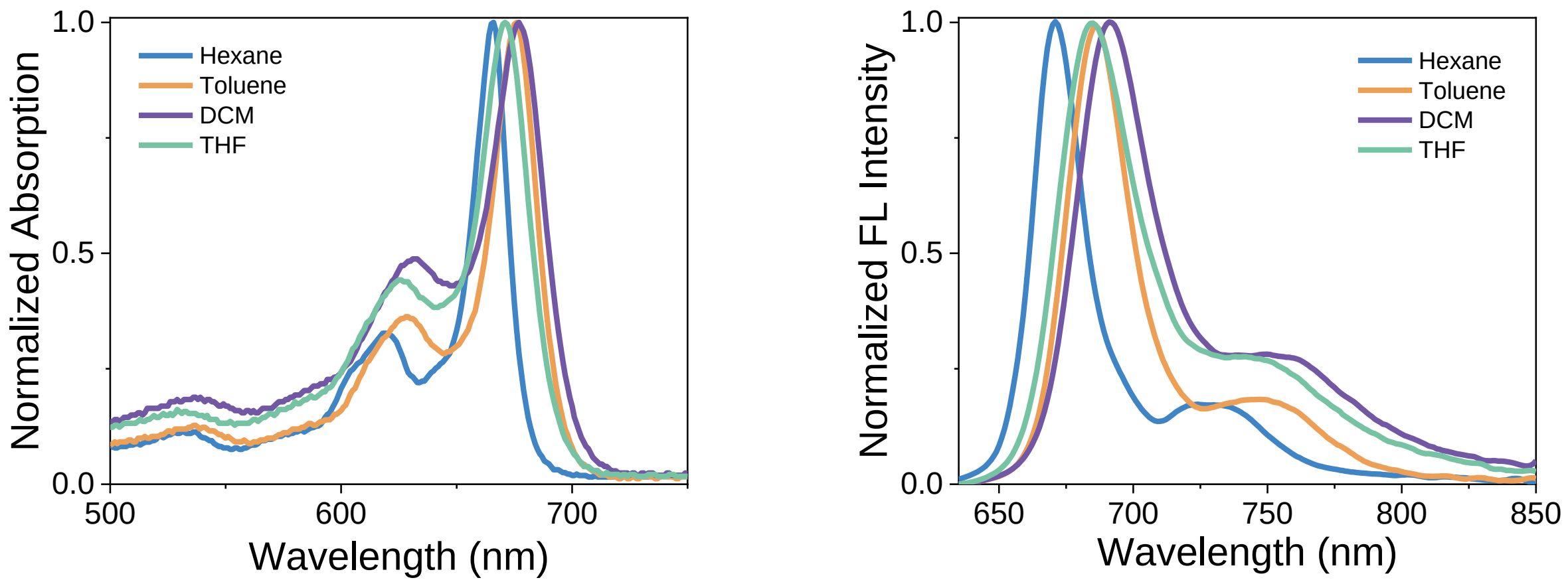

Figure S11. Absorption (left) and emission (right) spectra of compound **4e** (2 μM) recorded in different solvents. Excited at 630 nm.

5. Evaluation of the photostability

The photostability of molecule **3a** was systematically evaluated by monitoring the temporal evolution of its maximum absorbance under continuous illumination. All samples were irradiated with a 50 W white LED lamp for more than 30 min.[12] For a rigorous comparison, zinc phthalocyanine and methylene blue (two typical dyes with similar absorption ranges) were selected as reference materials. The absorption spectra were continuously recorded throughout the illumination process to track the photobleaching behavior.

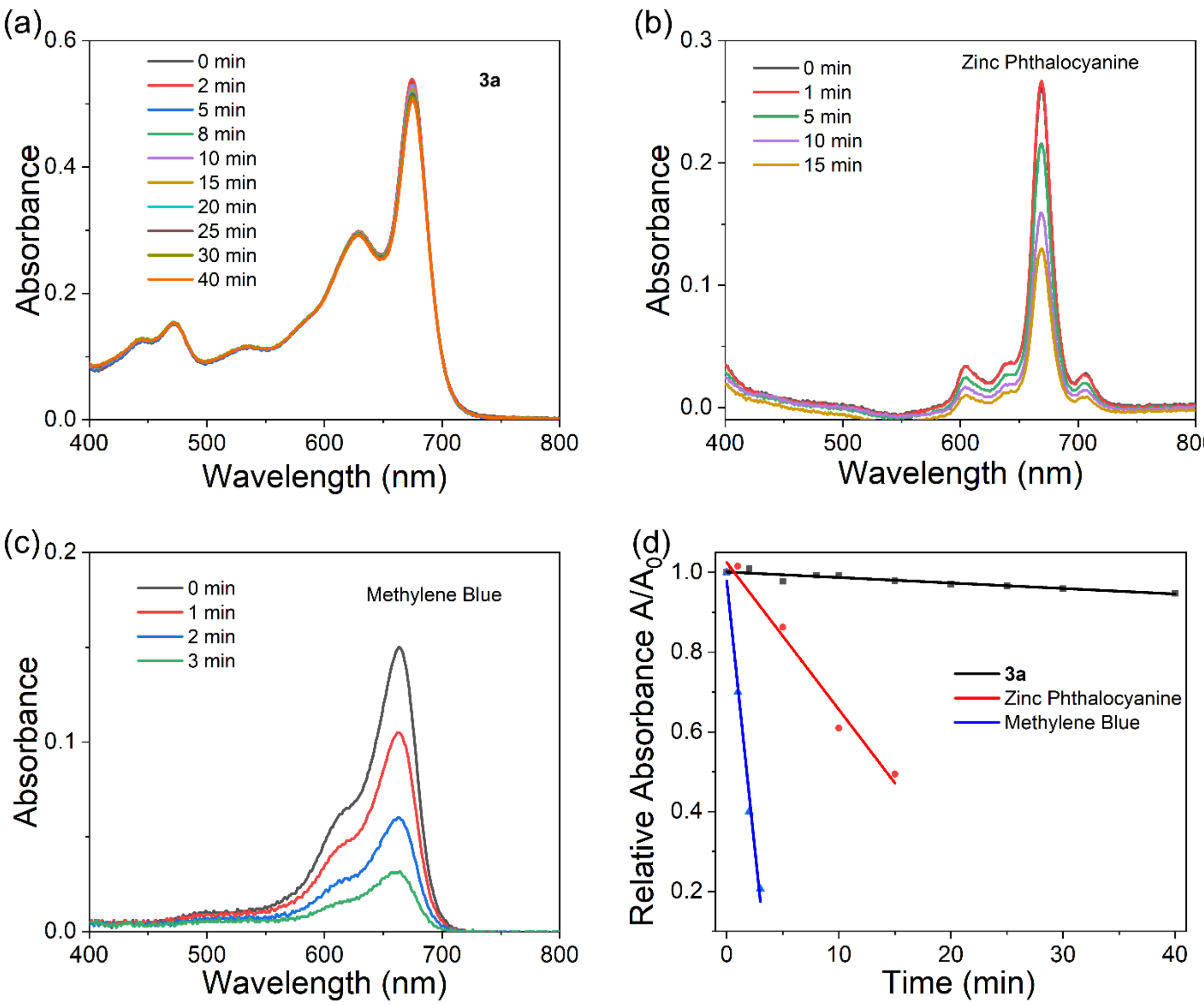

Figure S12. UV−Vis absorption spectral changes of (a) **3a** (5 μM), (b) zinc phthalocyanine (5 μM) and (c) methylene blue (5 μM) in DMF under continuous strong irradiation for over 40 min with a 50 W white LED lamp. (d) Absorption changes of **3a** (at 675 nm), Zinc Phthalocyanine (at 668 nm) and Methylene Blue (at 663 nm) as a function of irradiation time under continuous strong irradiation for 40 min with a 50 W white LED lamp.

6. Electrochemical data

Table S4. Electrochemical data acquired at 100 mV/s, and HOMO-LUMO gaps determined from spectroscopy of dyes **3a-c** and **4a-4e**[a].

Dyes	$E_{1/2}^{red}$ (V)	E_{red}^{onest} (V)	$E_{1/2}^{ox}$ (V)	E_{ox}^{onest} (V)	LUMO (eV)	HOMO (eV)	E_g^e (eV)
**1a**	-0.96	-0.87		1.48	-3.53	-5.88	2.35
**3a**	-0.55; -1.00; -1.26; -1.69	-0.48	1.36	1.30	-3.92	-5.70	1.78
**3b**	-0.56; -0.99; -1.26; -1.68	-0.50	1.38	1.29	-3.90	-5.69	1.79
**3c**	-0.56; -0.98; -1.24; -1.68	-0.48	1.36	1.25	-3.92	-5.65	1.73
**4a**	-0.59; -1.04; -1.30; -1.68	-0.51	1.35	1.25	-3.89	-5.65	1.76
**4b**	-0.59; -1.02; -1.28; -1.66	-0.50	1.34	1.26	-3.90	-5.66	1.76
**4c**	-0.59; -1.00; -1.26; -1.63	-0.47	1.33	1.13	-3.93	-5.63	1.60
**4d**	-0.38; -0.95; -1.27	-0.08	1.43	1.36	-4.32	-5.76	1.44
**4e**	-0.59; -1.02; -1.27; -1.67	-0.52	1.34	1.19	-3.88	-5.59	1.71

[a]$E_{1/2}^{red}$ = reversible reduction peak potentials; E_{red}^{onset} = the onset reduction potentials; E_{ox}^{onset} = the onset oxidation potentials; E_{LUMO} = -e (E_{red}^{onset} + 4.4); E_{HOMO} = -e (E_{ox}^{onset} + 4.4); E_g^e = bandgap, obtained from the intercept of the electrochemical data; $E_g^e = E_{LUMO} - E_{HOMO}$.

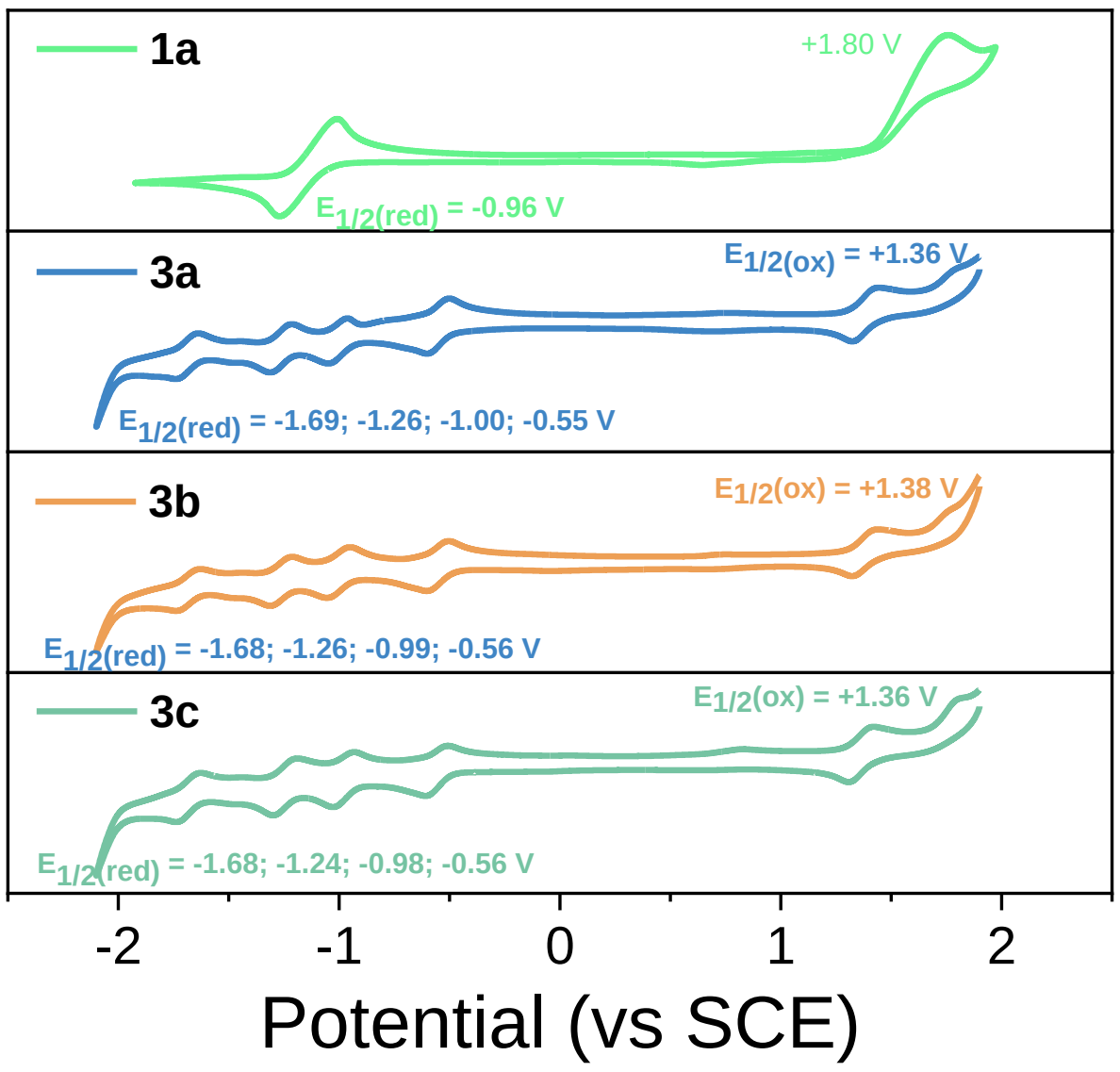

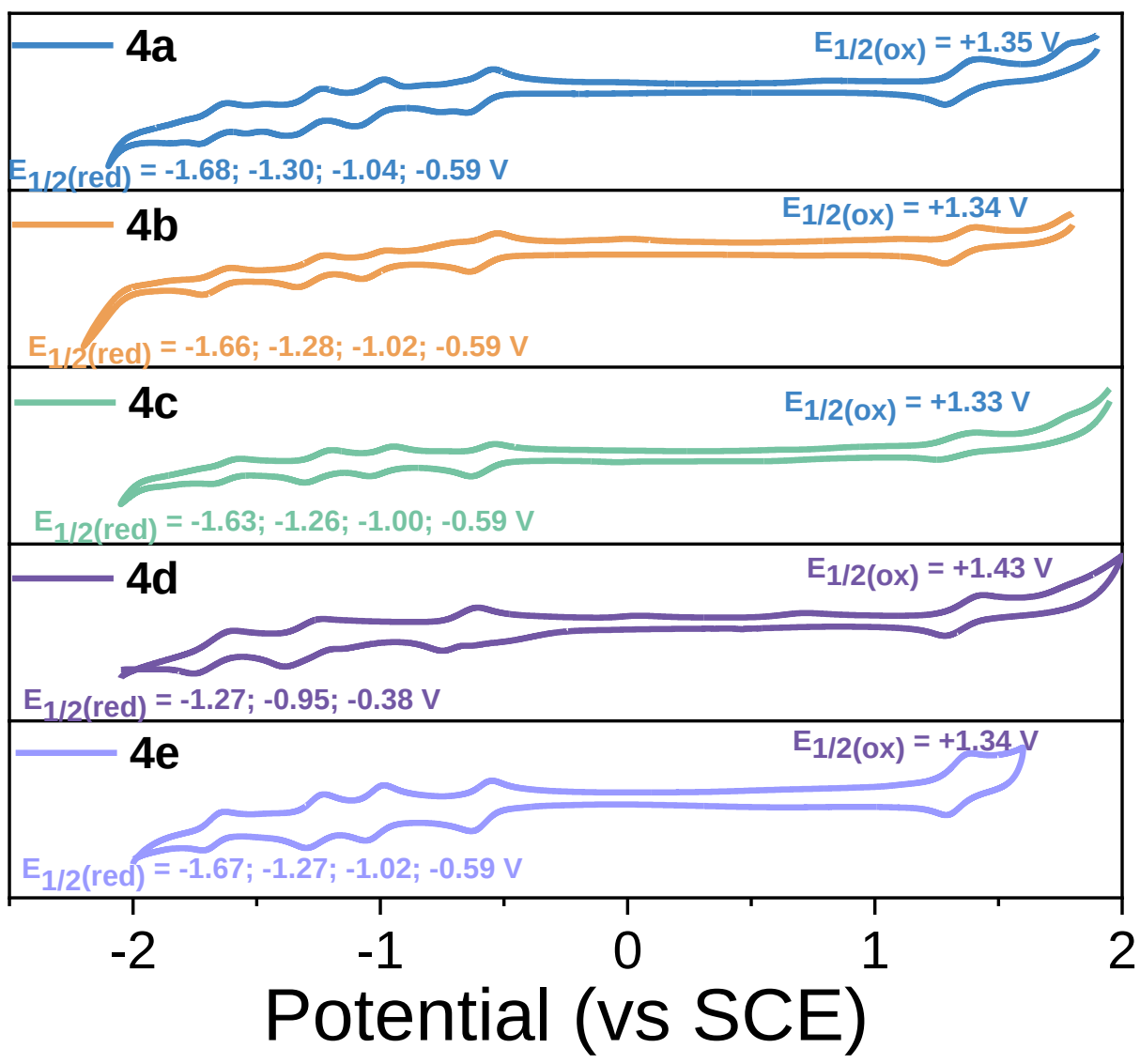

Figure S13. Cyclic voltammograms of 4.0 mM **1a, 3a, 3b, 3c, 4a, 4b, 4c, 4d** and **4e** measured in dichloromethane solution containing 0.1 M $TBAPF_6$ as the supporting electrolyte at room temperature. Glassy carbon electrode as a working electrode, and the scan rate at 100 mV s^{-1}. * CV plotting convention: the IUPAC convention.

7. DFT calculations

The ground state geometry was optimized by using DFT method at PBE1PBE/6-31G(d) level. The same method was used for vibrational analysis to verify that the optimized structures correspond to local minima on the energy surface. TD-DFT computations were used the optimized ground state geometries under the PBE1PBE/6-31+G(d,p) theoretical level. The calculated molecules in dichloromethane were done using the Self-Consistent Reaction Field (SCRF) method and Polarizable Continuum Model (PCM). All of the calculations were carried out by the methods implemented in Gaussian 09 package.[13]

Table S5. Selected electronic excitation energies (eV) and oscillator strengths (f), configurations ofthe low-lying singlet excited states of oligomers **3a** and **4d** calculated by TDDFT//B3LYP/6-31+G(d,p),based on the optimized ground state geometries.

	**Electronic transition**	**TD//B3LYP/6-31+G(d, p)**			
		**Energy/ eV [a]**	***f* [b]**	**Composition [c]**	**CI [d]**
**3a**	S0→S1	1.9699 eV 629.40 nm	0.5385	HOMO → LUMO	0.6738
				HOMO → LUMO +2	0.2041
	S0→S2	2.1615 eV 573.60 nm	0.0108	HOMO -1 → LUMO +1	0.6994
	S0→S3	2.4212 eV 512.09 nm	0.9982	HOMO → LUMO	0.2020
				HOMO → LUMO +2	0.6602
**4d**	S0→S1	1.9609 eV 632.29 nm	0.7130	HOMO → LUMO	0.6769
				HOMO → LUMO +2	0.1967
	S0→S2	2.2196 eV 558.59 nm	0.0097	HOMO → LUMO +1	0.6994
	S0→S3	2.3949 eV 517.69 nm	0.5220	HOMO -1 → LUMO	0.1815
				HOMO → LUMO	0.1811
				HOMO → LUMO +2	0.6458

[a] Only the selected low-lying excited states are presented. [b] Oscillator strength. [c] Only the main configurations are presented. [d] The CI coefficients are in absolute values.

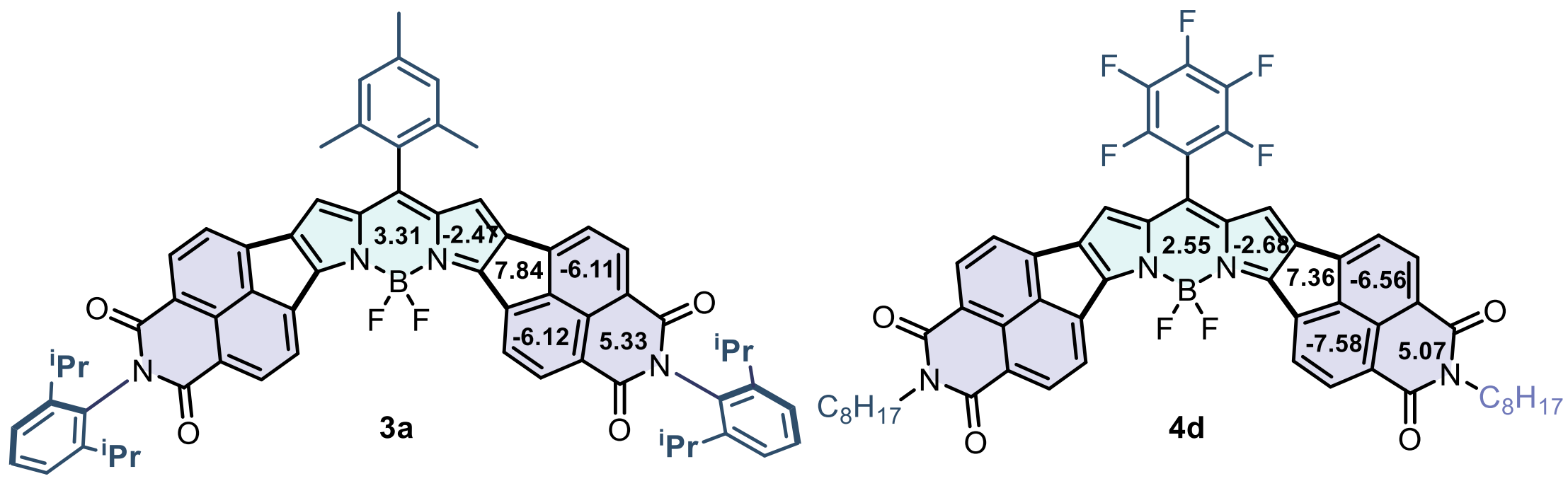

Figure S14. Calculated NICS(0) values at various positions of **3a** and **4d**, calculated at the B3LYP/6-31+G(d, p) level.

Optimized Geometries of the Compounds.

3a, optimized S0 state Geometry.

F	-0.00808700	0.72006400	-1.99386200
F	0.00047500	-0.26953500	0.07180600
N	1.23511900	1.79918700	-0.23112900
N	-1.23993700	1.79708500	-0.22189600
C	1.21231400	3.19845600	-0.10881300
C	3.37214700	2.56309600	0.00422100
C	2.51519500	1.42274000	-0.15758000
C	-0.00360600	3.89741100	-0.06285700
C	2.55160400	3.67760700	0.03170300
H	2.81792600	4.71878000	0.15096800
C	-1.21865200	3.19640600	-0.09993100
C	-2.55770300	3.67326400	0.05021100
C	-2.51878200	1.41844900	-0.13890200
C	-3.37652300	2.55734100	0.02889100
B	-0.00315900	0.93036800	-0.62914100
H	-2.82490300	4.71399100	0.17130000
C	3.07177900	-1.16683500	-0.29494200
C	3.30210000	0.19680400	-0.19120600
C	4.64192800	0.65883100	-0.05353100
C	5.73398100	-0.19955300	-0.03132500

C	5.49122200	-1.58818900	-0.14963800
C	4.18343900	-2.04856200	-0.27561100
H	2.06322900	-1.55609500	-0.37847500
C	4.75457800	2.07441400	0.07169900
C	7.02417300	0.36473200	0.11294800
H	4.02479700	-3.11909600	-0.35644300
C	7.15034200	1.74352700	0.23048200
C	6.02324300	2.61064100	0.21460700
H	8.14767100	2.15796200	0.33861600
H	6.17902900	3.68080800	0.31493600
C	6.64831100	-2.51637000	-0.11857700
C	8.19862200	-0.53718300	0.12643800
O	6.52032200	-3.72810600	-0.19729300
O	9.34474300	-0.12778200	0.22698900
N	7.92860000	-1.92473400	0.01237600
C	-6.02601400	2.60011700	0.25994600
C	-4.75755300	2.06622500	0.10677600
C	-4.64336100	0.65084300	-0.01928700
C	-5.73371100	-0.20945700	0.01094300
C	-7.02363300	0.35234400	0.16685300
C	-7.15116100	1.73076300	0.28740300
H	-6.18292400	3.66997400	0.36183500
C	-3.30378000	0.19115800	-0.16684900
C	-5.48954900	-1.59748600	-0.11142500
H	-8.14785700	2.14295500	0.40910400
C	-4.18209700	-2.05545300	-0.24895500
C	-3.07189900	-1.17200500	-0.27322100
H	-4.02265500	-3.12529000	-0.33712300
H	-2.06334200	-1.55949800	-0.36453200
C	-8.19471300	-0.55312500	0.20840900
C	-6.64637200	-2.52625600	-0.09141900
O	-9.33761600	-0.14849300	0.35471300

O	-6.52013700	-3.73486000	-0.21061900
N	-7.92481700	-1.93862600	0.07169900
C	-0.00458700	5.38866900	0.06671700
C	-0.00669300	6.18661100	-1.09698900
C	0.00159600	5.98566200	1.34495200
C	-0.00379500	7.57768700	-0.95616700
C	0.00435000	7.38145600	1.43293100
C	-0.00170900	8.19615700	0.29734700
C	-0.03039600	9.70155500	0.42083900
H	0.47561600	10.18284100	-0.42265100
H	-1.06197100	10.07775300	0.43818500
H	0.45294400	10.03757000	1.34413500
C	0.00937600	5.15115200	2.60677400
H	0.01232100	5.79012000	3.49450700
H	-0.86936200	4.49842200	2.66571200
H	0.89097500	4.50158000	2.65750100
C	-0.00793300	5.56674100	-2.47670700
H	0.87250600	4.93360100	-2.63697100
H	-0.88691100	4.93073600	-2.63358500
H	-0.01073400	6.34014300	-3.25011800
H	0.01272200	7.84260900	2.41841500
H	-0.00181300	8.19333700	-1.85331900
C	-9.06837000	-2.84191800	0.10095200
C	-9.75724400	-3.11469800	-1.10178900
C	-9.45421400	-3.41983600	1.33090800
C	-10.84838600	-3.99296500	-1.03852000
C	-10.56039200	-4.28133400	1.32319600
C	-11.25396000	-4.57246300	0.15618500
H	-11.38956300	-4.21762700	-1.95441600
H	-10.87432200	-4.73393600	2.26065100
H	-12.10567800	-5.24719700	0.17766700
C	9.07414400	-2.82571000	0.03472900

C	9.65990100	-3.21993600	-1.18881900
C	9.56541500	-3.27940500	1.27911400
C	10.76240100	-4.08423600	-1.13049300
C	10.66476800	-4.14950500	1.26326100
C	11.26430600	-4.55080500	0.07700700
H	11.22850800	-4.39700000	-2.06168800
H	11.05553400	-4.51198200	2.21096000
H	12.11779600	-5.22343800	0.09349200
C	-8.76666400	-3.25752800	2.69606200
H	-9.55315800	-3.50989800	3.41941800
C	-9.48493400	-2.51596700	-2.49107300
H	-9.93191500	-3.24128900	-3.18359000
C	9.06550000	-2.88978500	2.67949400
H	9.40752800	-3.71295800	3.32047700
C	9.19745900	-2.85125600	-2.60756100
H	10.09250200	-3.00371100	-3.22493400
C	9.77650100	-1.62131100	3.19894400
H	10.86476400	-1.74057300	3.16300800
H	9.52471200	-0.74151500	2.60147200
H	9.48864300	-1.42928000	4.24011500
C	7.54582800	-2.80814300	2.90868000
H	7.10999300	-1.87996500	2.52711200
H	7.02080000	-3.65126400	2.45067400
H	7.34789900	-2.83058300	3.98683600
C	8.14203300	-3.84810100	-3.13356200
H	8.51058600	-4.87762700	-3.07005900
H	7.21381700	-3.79786800	-2.55888500
H	7.91249300	-3.63220100	-4.18443800
C	8.76981600	-1.39695700	-2.87362500
H	7.75647900	-1.18284500	-2.52105000
H	9.45572600	-0.68156200	-2.41092900
H	8.77221300	-1.21875100	-3.95543600

C	-8.28543700	-1.85284800	3.10090500
H	-7.33962000	-1.57652200	2.62541300
H	-9.02864400	-1.08595200	2.86456700
H	-8.11203900	-1.83551100	4.18338700
C	-7.65218900	-4.30767100	2.89467400
H	-8.03785900	-5.32114300	2.74005900
H	-6.82712200	-4.16206400	2.19295000
H	-7.25554400	-4.24580700	3.91572600
C	-10.25998800	-1.19631900	-2.69676500
H	-11.32953000	-1.33736000	-2.50688500
H	-9.90964100	-0.41077000	-2.02252800
H	-10.14018700	-0.84751200	-3.73006800
C	-8.02108600	-2.37734200	-2.94552000
H	-7.52208800	-1.50872400	-2.50560300
H	-7.43612500	-3.27036500	-2.70748200
H	-7.99885400	-2.23755500	-4.03271500

SCF done: -3292.913396 Hartree

No imaginary Frequency.

4d, optimized S0 state Geometry.

F	0.01918400	-0.69970000	2.02402800
F	0.00235900	-1.66832200	-0.05072700
N	-1.23640100	0.39170700	0.27645200
N	1.23809200	0.39860600	0.25712200
C	-1.22203100	1.78893700	0.15297900
C	-3.37553400	1.13989300	0.00905100
C	-2.51247100	0.00494300	0.18433500
C	-0.00452800	2.48542300	0.11597700
C	-2.56329300	2.25813000	-0.01068900
H	-2.84370200	3.29332000	-0.14570900
C	1.21785900	1.79842800	0.15203000
C	2.56076800	2.27789100	0.03596300

C	2.51845800	0.02164100	0.19264300
C	3.37858800	1.16408000	0.05483300
B	0.00546100	-0.47662100	0.66283900
H	2.84092200	3.31720800	-0.06024100
C	-3.05092600	-2.58810600	0.30876900
C	-3.28990000	-1.22589200	0.20361600
C	-4.63120300	-0.77308200	0.04573200
C	-5.71643200	-1.63948900	0.00612500
C	-5.46429000	-3.02632400	0.12724000
C	-4.15570700	-3.47777600	0.27178100
H	-2.04101700	-2.97026500	0.40625500
C	-4.75312400	0.64143900	-0.08111300
C	-7.00791400	-1.08409500	-0.16069300
H	-3.99052700	-4.54718000	0.35457200
C	-7.14274800	0.29284900	-0.28307900
C	-6.02247800	1.16866900	-0.24588800
H	-8.14064000	0.69973600	-0.41258200
H	-6.18443100	2.23762200	-0.34897200
C	-6.61725600	-3.96017900	0.08359900
C	-8.17228000	-1.99920400	-0.20505400
O	-6.48721400	-5.17357500	0.16125100
O	-9.31968300	-1.60670500	-0.36279300
N	-7.89396200	-3.37540600	-0.05030100
C	-9.04858600	-4.29738300	-0.08630500
H	-9.89266900	-3.75812800	0.34413300
H	-8.79343700	-5.14367100	0.55195200
C	-9.37508200	-4.76902400	-1.50327000
H	-10.23484600	-5.44798700	-1.47869900
H	-9.62923300	-3.92078000	-2.14631000
H	-8.52795400	-5.30798100	-1.93853100
C	6.03158700	1.21224700	-0.12388500
C	4.76155300	0.67543500	-0.00058200

C	4.64547600	-0.74103300	0.10911600
C	5.73711400	-1.60014100	0.09322300
C	7.02912800	-1.03495700	-0.03101800
C	7.15823200	0.34391700	-0.13689400
H	6.18929800	2.28307000	-0.21312500
C	3.30339700	-1.20393300	0.22497200
C	5.49071000	-2.98952900	0.19528300
H	8.15669100	0.75835100	-0.23405500
C	4.18157800	-3.45065100	0.29909400
C	3.07034900	-2.56842600	0.31236800
H	4.02131800	-4.52175700	0.36842400
H	2.06050400	-2.95733300	0.37958100
C	8.20034500	-1.94217100	-0.04973500
C	6.65067400	-3.91557800	0.17665300
O	9.34920400	-1.54094500	-0.17045600
O	6.52649000	-5.13041000	0.24003700
N	7.92676300	-3.32149200	0.08456600
C	9.08803300	-4.23574500	0.07366800
H	8.82024100	-5.08936600	0.69680500
H	9.91580800	-3.69504300	0.53299100
C	9.45824700	-4.69243600	-1.33743400
H	10.32152300	-5.36598400	-1.29412300
H	8.62764500	-5.23292800	-1.80167600
H	9.72503600	-3.83678600	-1.96536300
C	-0.01827300	3.96840000	-0.00130000
C	-0.56526700	4.76911800	1.00952800
C	0.50461800	4.62488600	-1.12224500
C	-0.58815000	6.15834000	0.91627900
C	0.49158400	6.01264000	-1.23498300
C	-0.05744400	6.78144400	-0.21106400
F	-1.07626500	4.20520800	2.10984500
F	1.02857500	3.91887600	-2.13230500

F	0.99377900	6.60746500	-2.32043600
F	-1.11041400	6.89361600	1.90123000
F	-0.07498900	8.11025600	-0.31013100

SCF done: -2894.512844 Hartree

No imaginary Frequency.

8. OFET characterization

Organic field-effect transistors (OFETs) with a bottom-gate top-contact (BGTC) structure were fabricated. The semiconductor was added to PhCl and shaken for 4 hours at room temperature to ensure uniform dispersion, yielding a semiconductor solution. The n-type doped silicon substrate, used as the gate electrode, was sequentially cleaned with piranha solution and deionized water, dried with nitrogen gas, spin-coated with Cytop, and then dried. In a glovebox, the solution was spin-coated onto these substrates at a speed of 3000 rpm for 40 seconds. Subsequently, Au was thermally evaporated onto the organic layer through a shadow mask to form source and drain electrodes with a thickness of 40 nm, while the channel length and width were 100 and 1000 micrometers, respectively. All electrical performance tests were conducted at room temperature under ambient conditions.

The evaluations of the OFETs were carried out in atmosphere using a Keithley 4200 parameter analyzer on a probe stage.[14] The carrier mobility, μ, was calculated from the data in the saturated regime according to the equation $I_{DS} = (W/2L)C_i\mu(V_G–V_T)^2$, where I_{SD} is the drain current in the saturated regime. W and L are, respectively, the semiconductor channel width and length. Ci is the capacitance per unit area of the gate dielectric layer, and VGand VTare the gate voltage and threshold voltage. $V_G–V_T$ of the device was determined from the relationship between the square root of I_{SD} at the saturated regime.

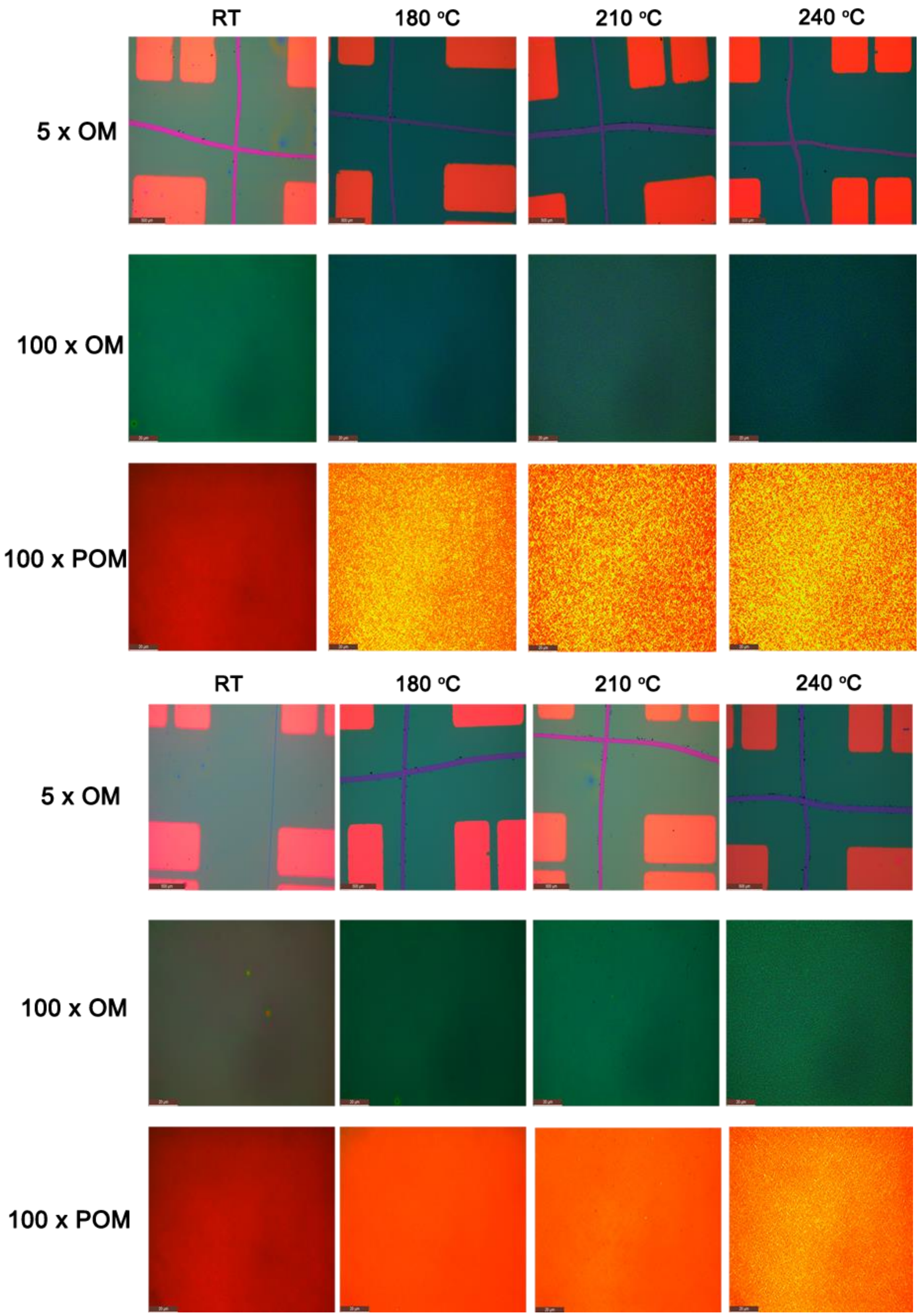

Figure S15. OM (5×, 100×) and POM (100×) images of **3b** (Top) and **3c** (Bottom) films annealed at RT, 180 °C, 210 °C, and 240 °C.

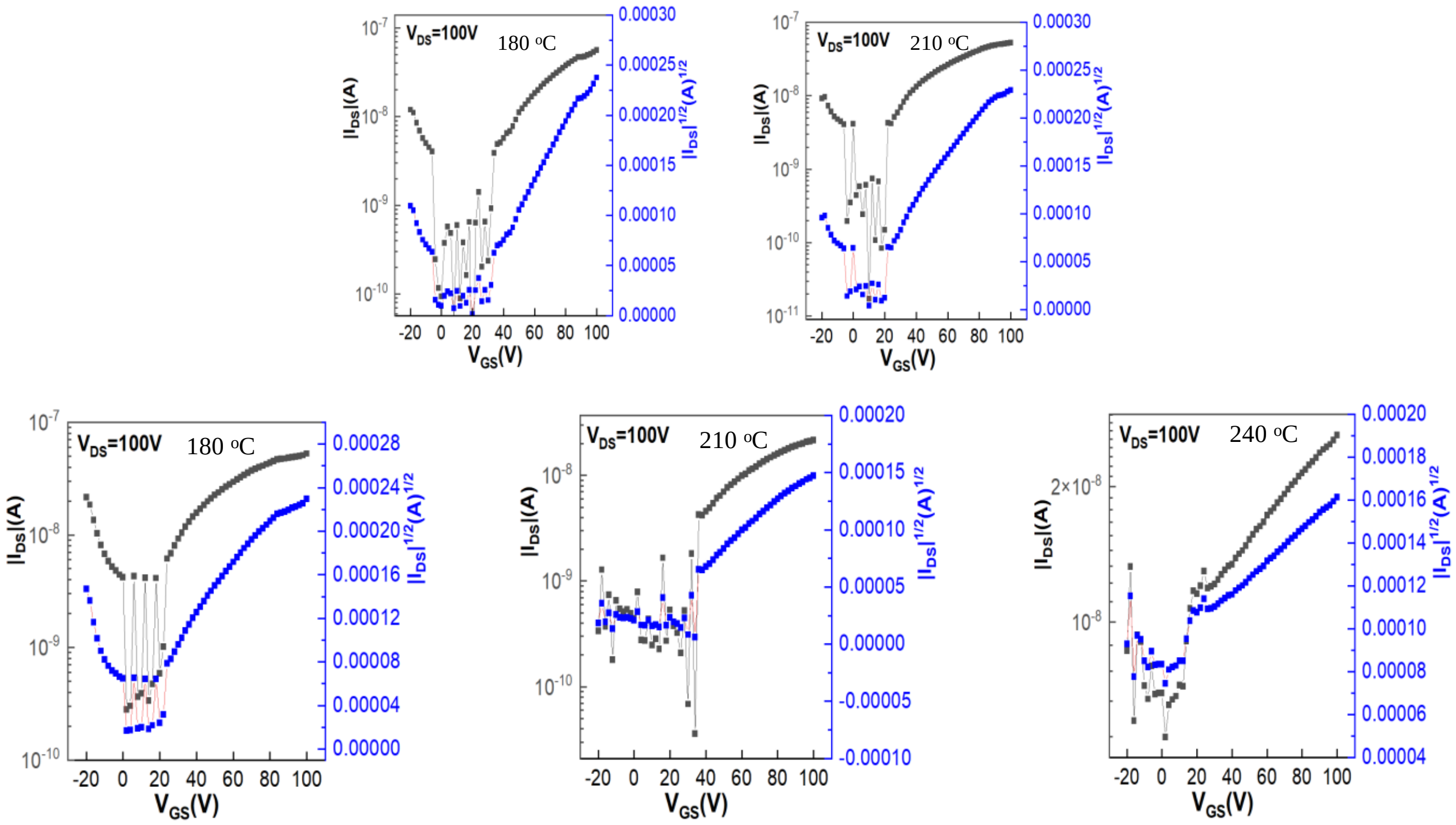

Figure S16. Representative I_{DS}–V_{GS} transfer curves of **3b** (Top) and **3c** (Bottom) OFETs at V_{DS}=100 V.

Table S6. Summarized OFET performance parameters for **3b** and **3c**.

Small molecule	Annealing temperature (°C)	$\mu_{e,max}$ ($cm^2 v^{-1} s^{-1}$)	$V_{th,max}$ (V)
**3b**	RT	/	/
	180	2.04×10^{-4}	19.66
	210	6.34×10^{-5}	27.51
	240	1.63×10^{-5}	18.93
**3c**	RT	/	/
	180	3.47×10^{-4}	28.18
	210	2.24×10^{-4}	20.30
	240	/	/

9. ^{1}H and ^{13}C NMR spectra for all new compounds

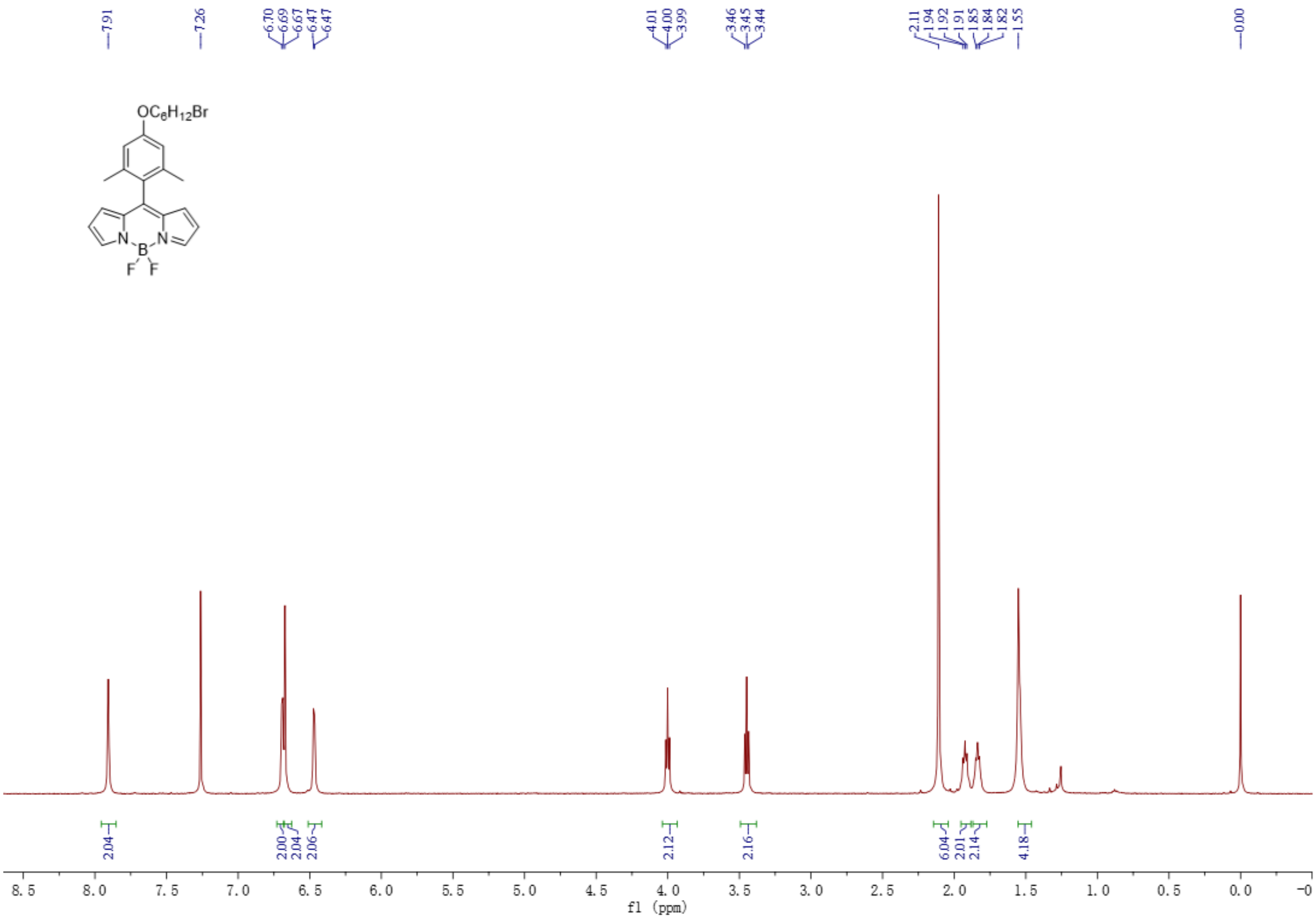

^{1}H NMR spectrum of **1e** in $CDCl_3$ (500 MHz)

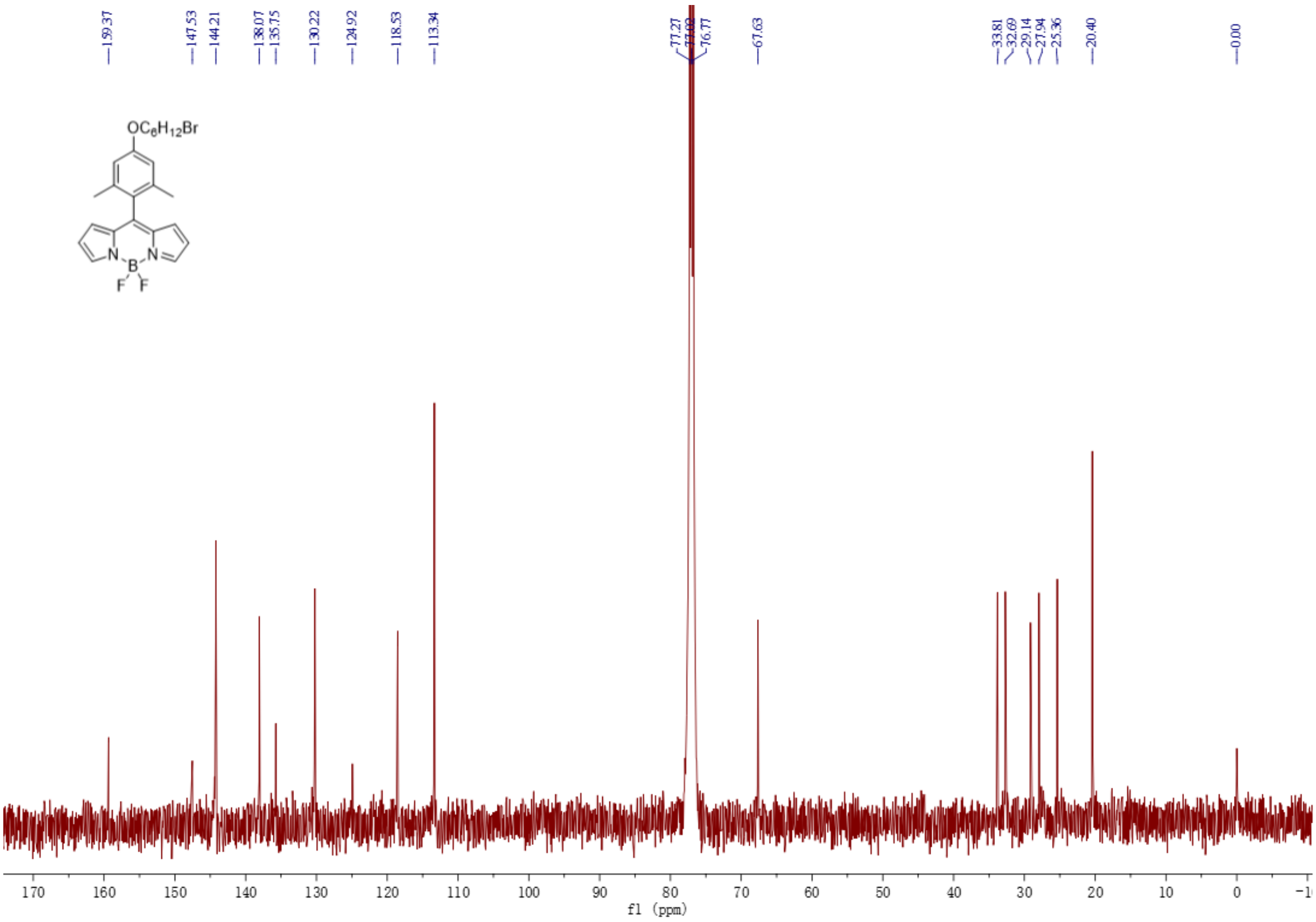

^{13}C NMR spectrum of **1e** in $CDCl_3$ (126 MHz)

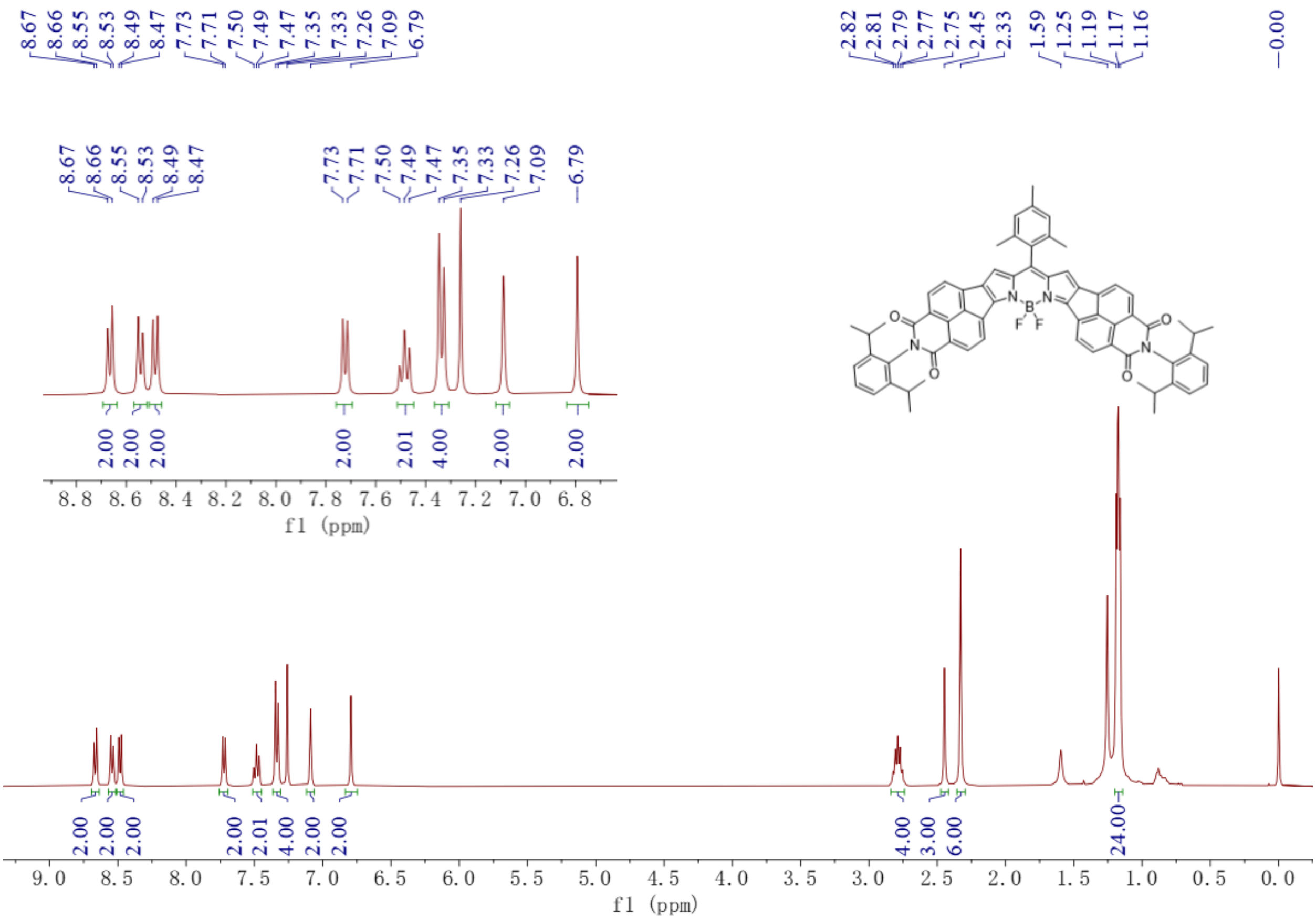

^{1}H NMR spectrum of **3a** in $CDCl_3$ (400 MHz)

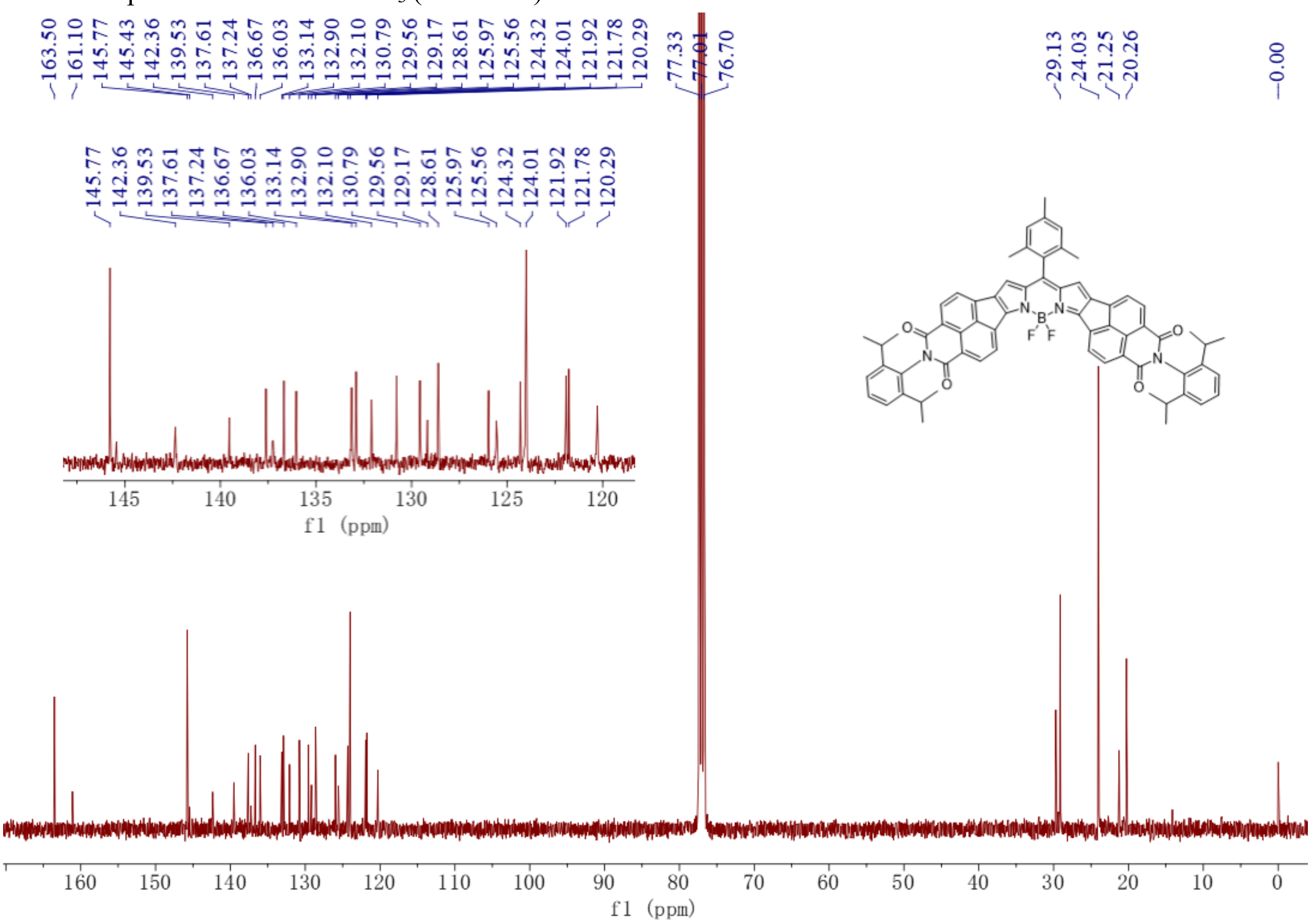

^{13}C NMR spectrum of **3a** in $CDCl_3$ (101 MHz)

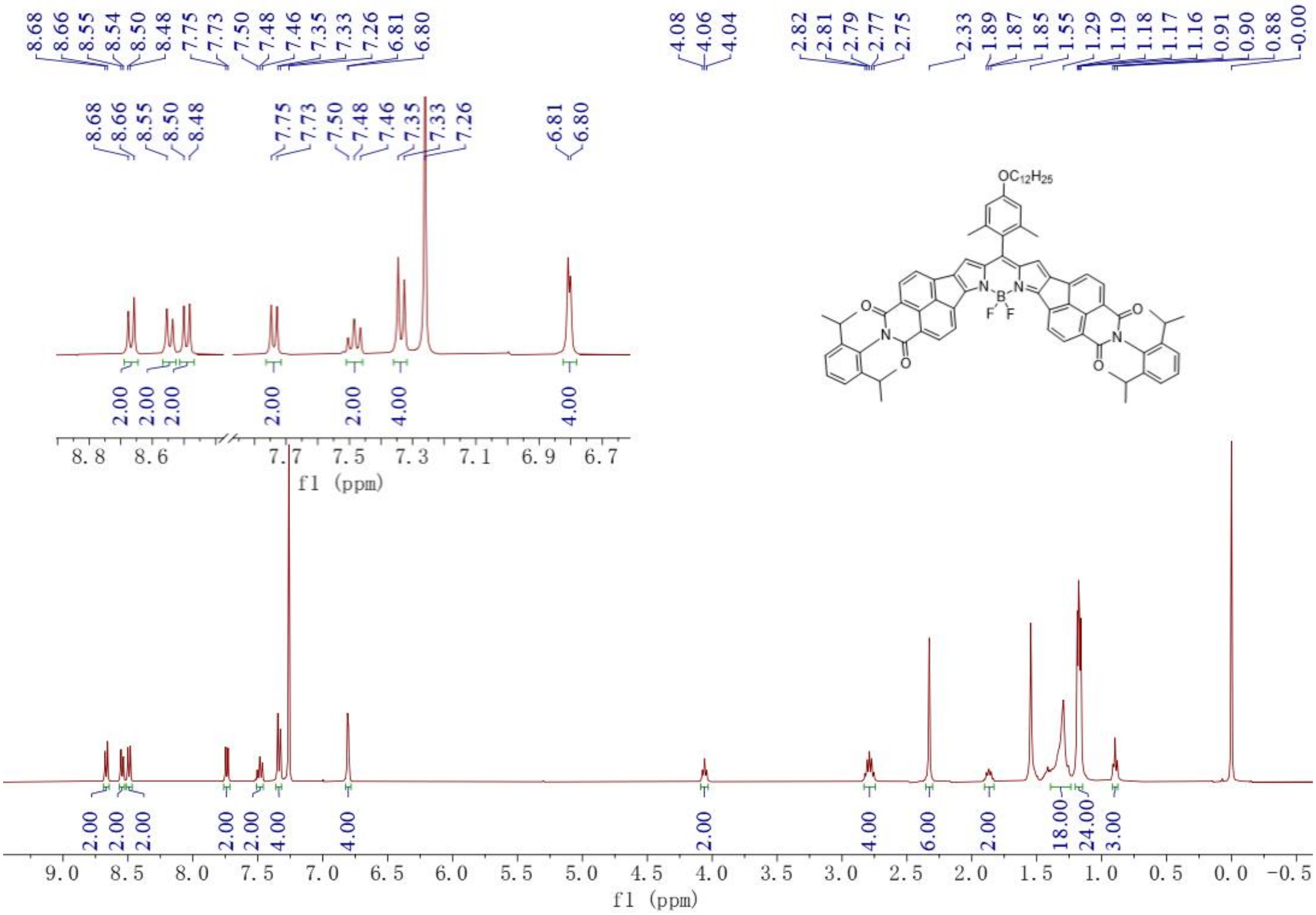

^{1}H NMR spectrum of **3b** in $CDCl_3$ (400 MHz)

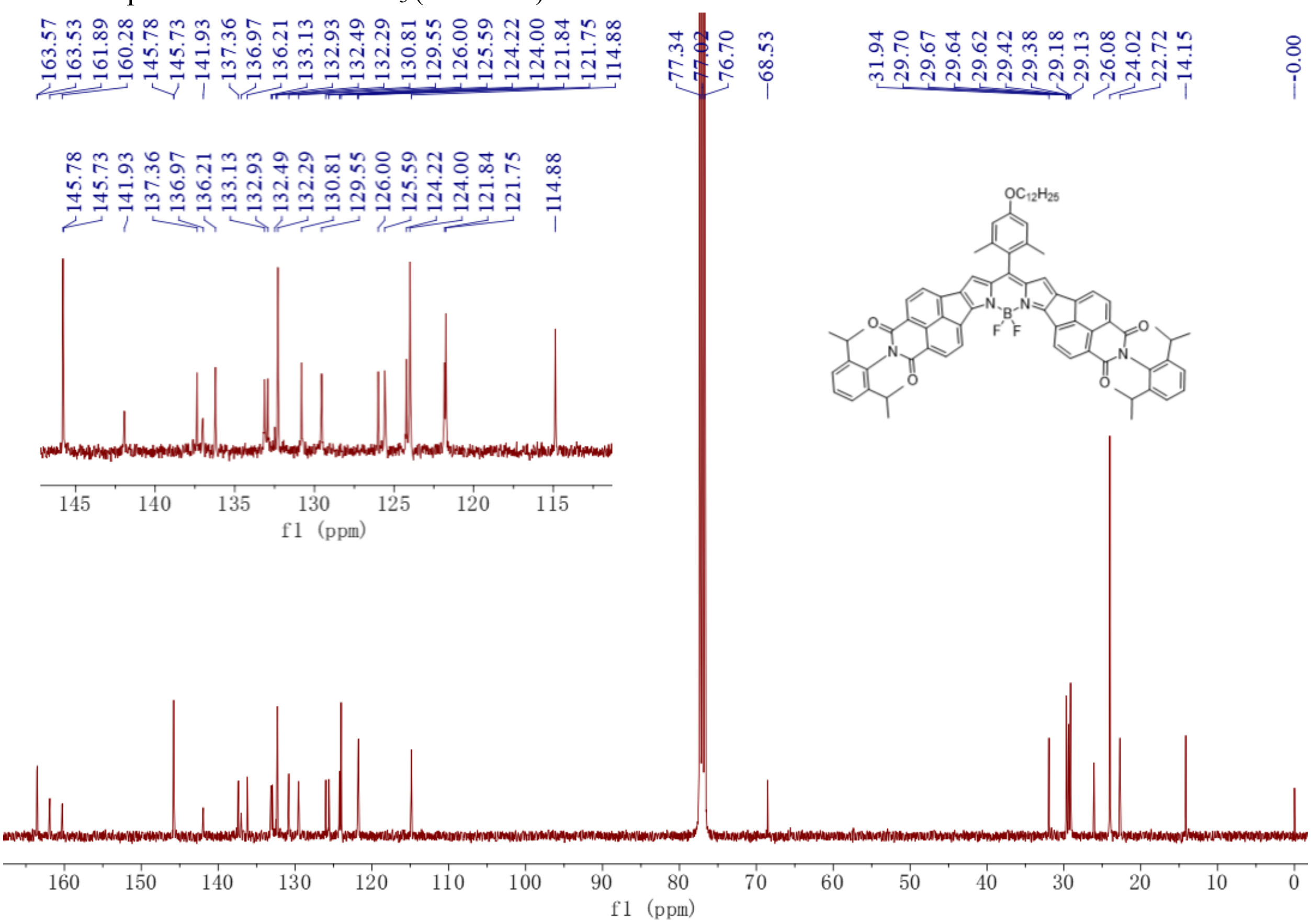

^{13}C NMR spectrum of **3b** in $CDCl_3$ (101 MHz)

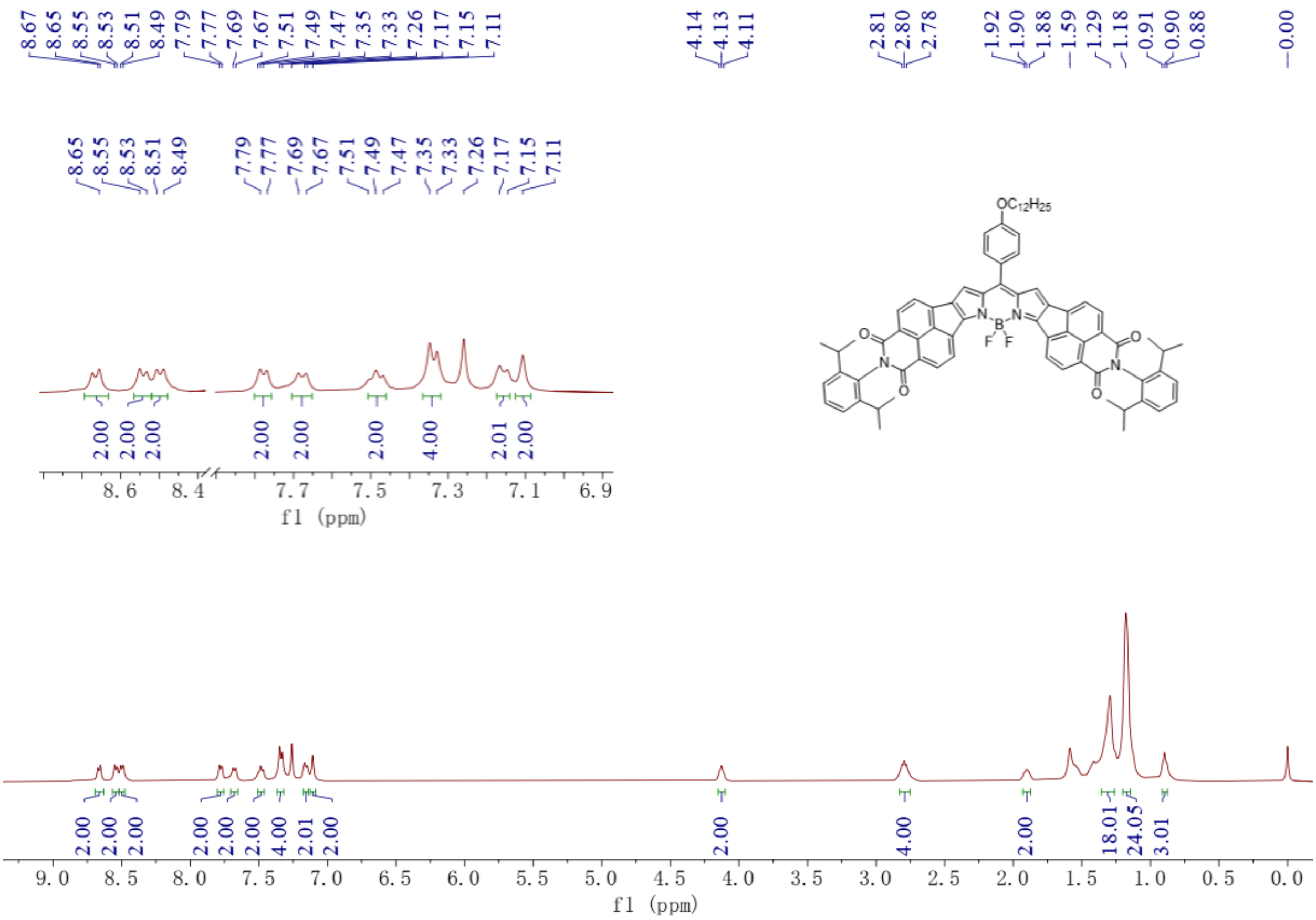

^{1}H NMR spectrum of **3c** in $CDCl_3$ (400 MHz)

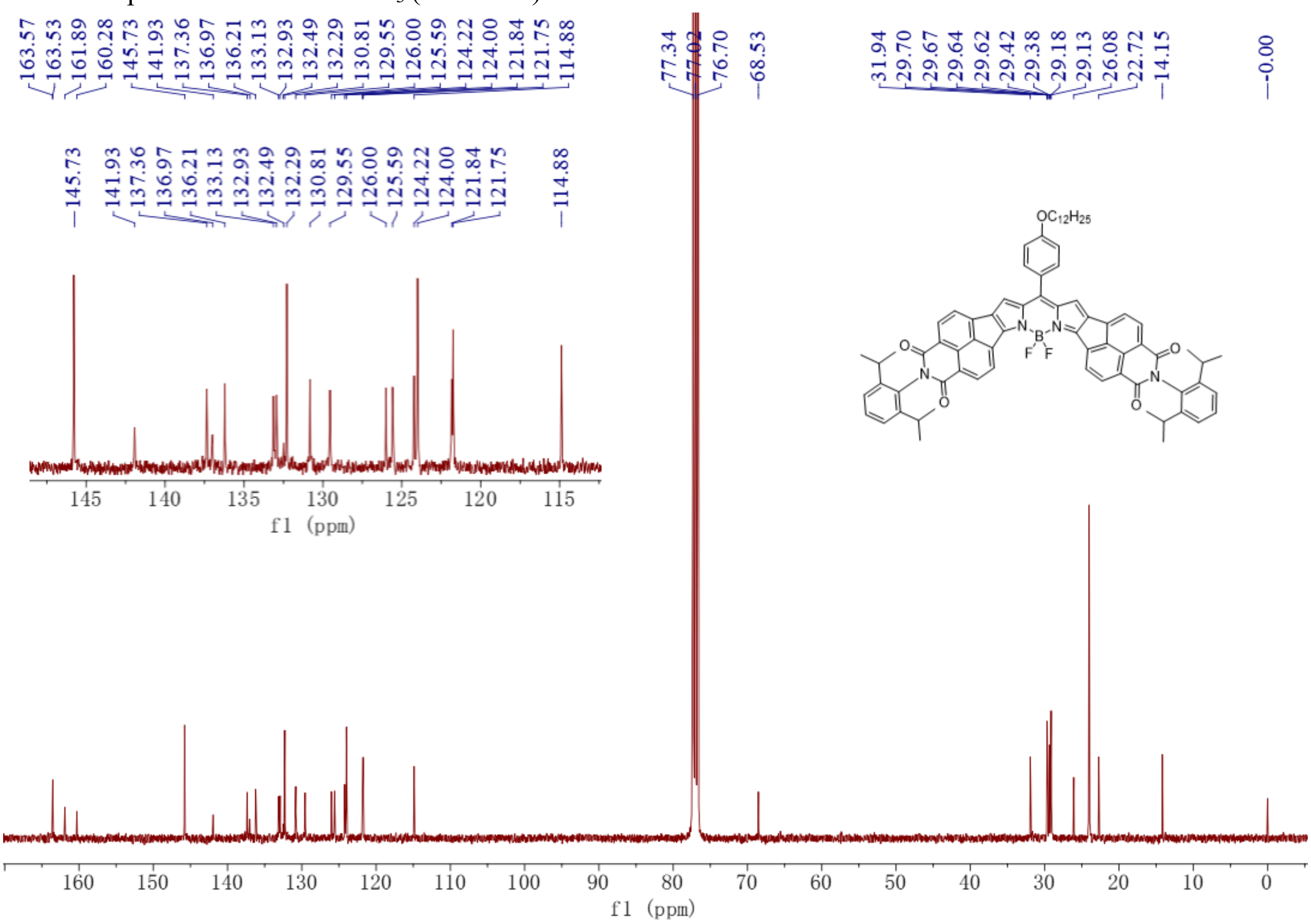

^{13}C NMR spectrum of **3c** in $CDCl_3$ (101 MHz)

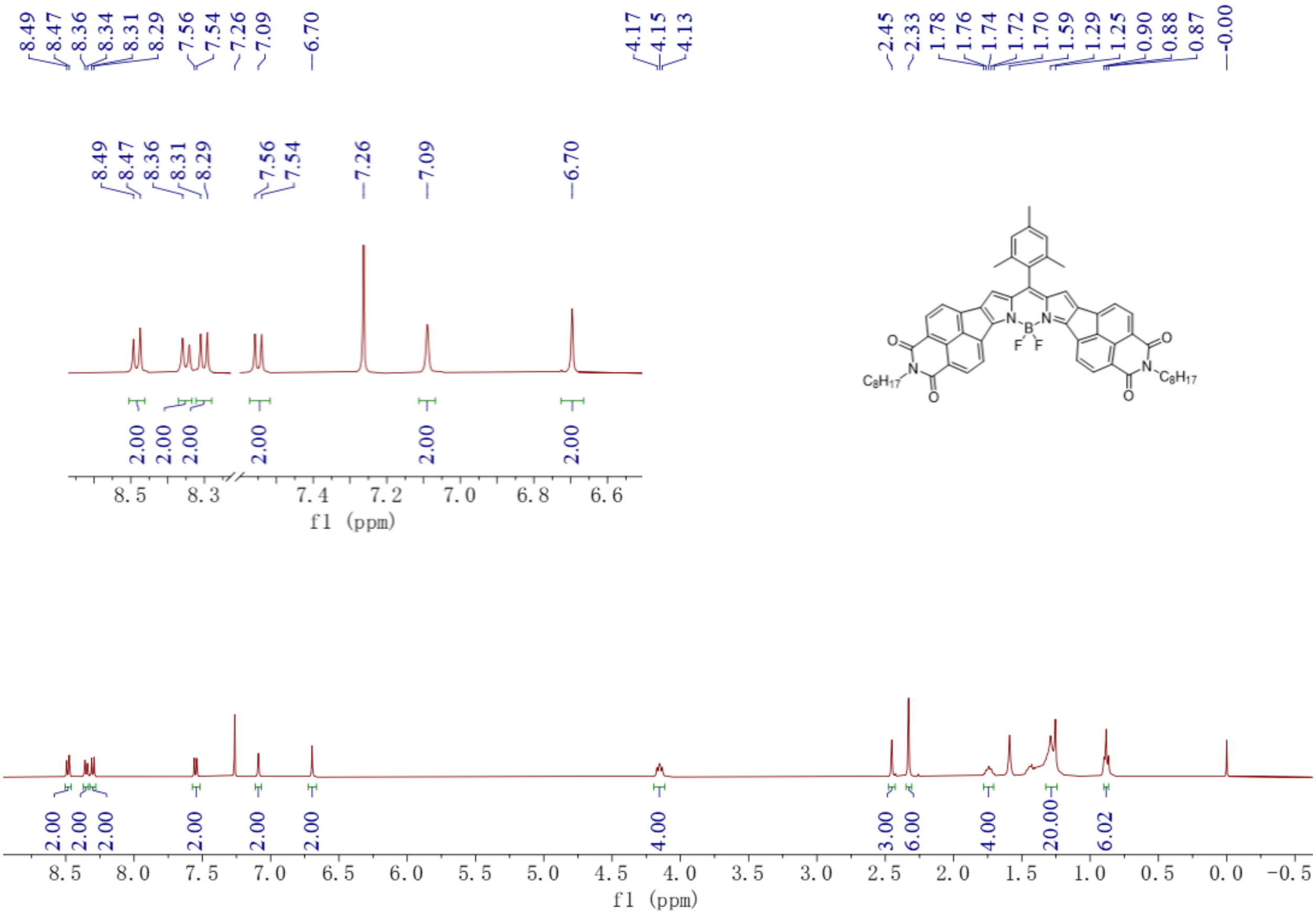

^{1}H NMR spectrum of **4a** in $CDCl_3$ (400 MHz)

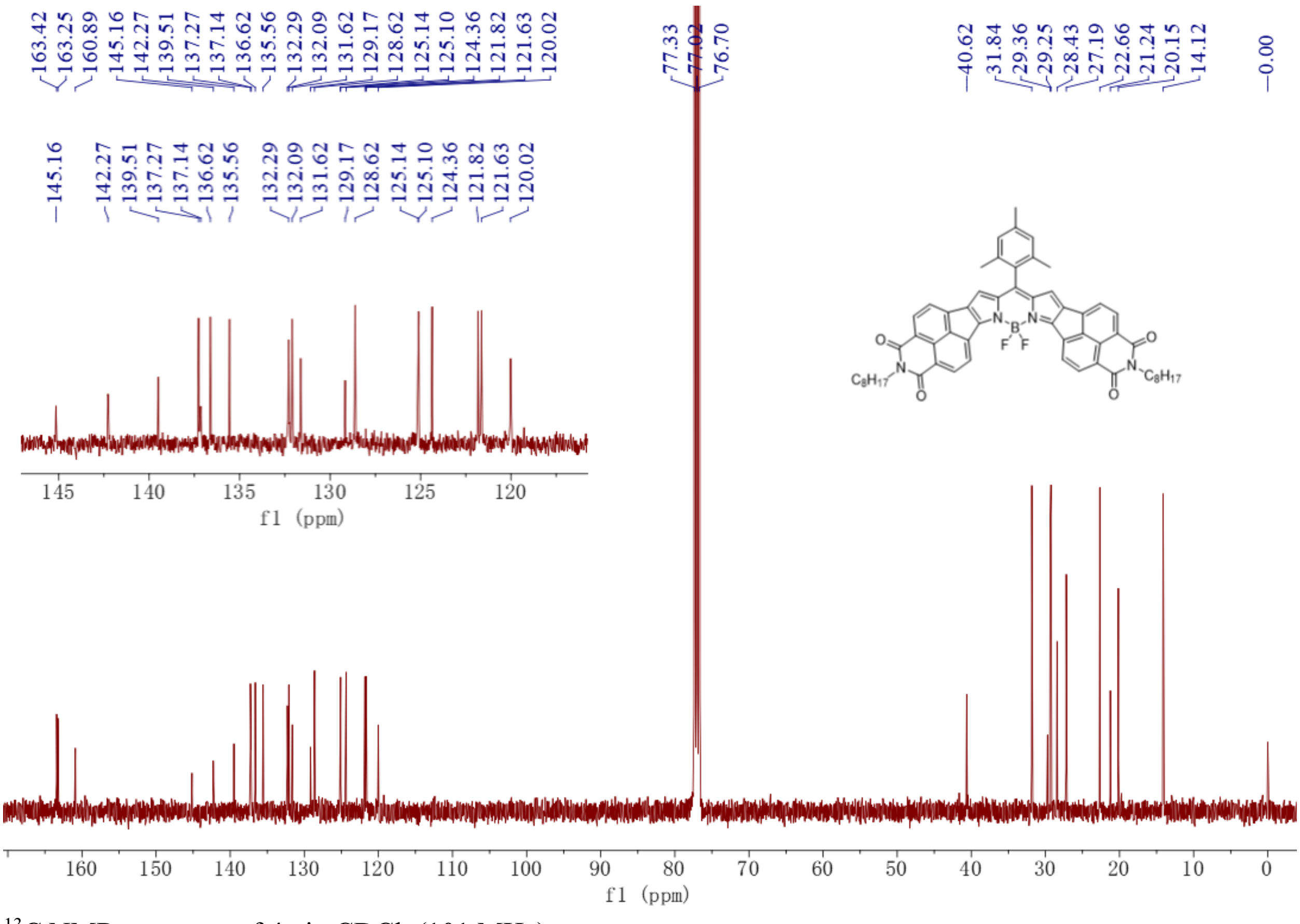

^{13}C NMR spectrum of **4a** in $CDCl_3$ (101 MHz)

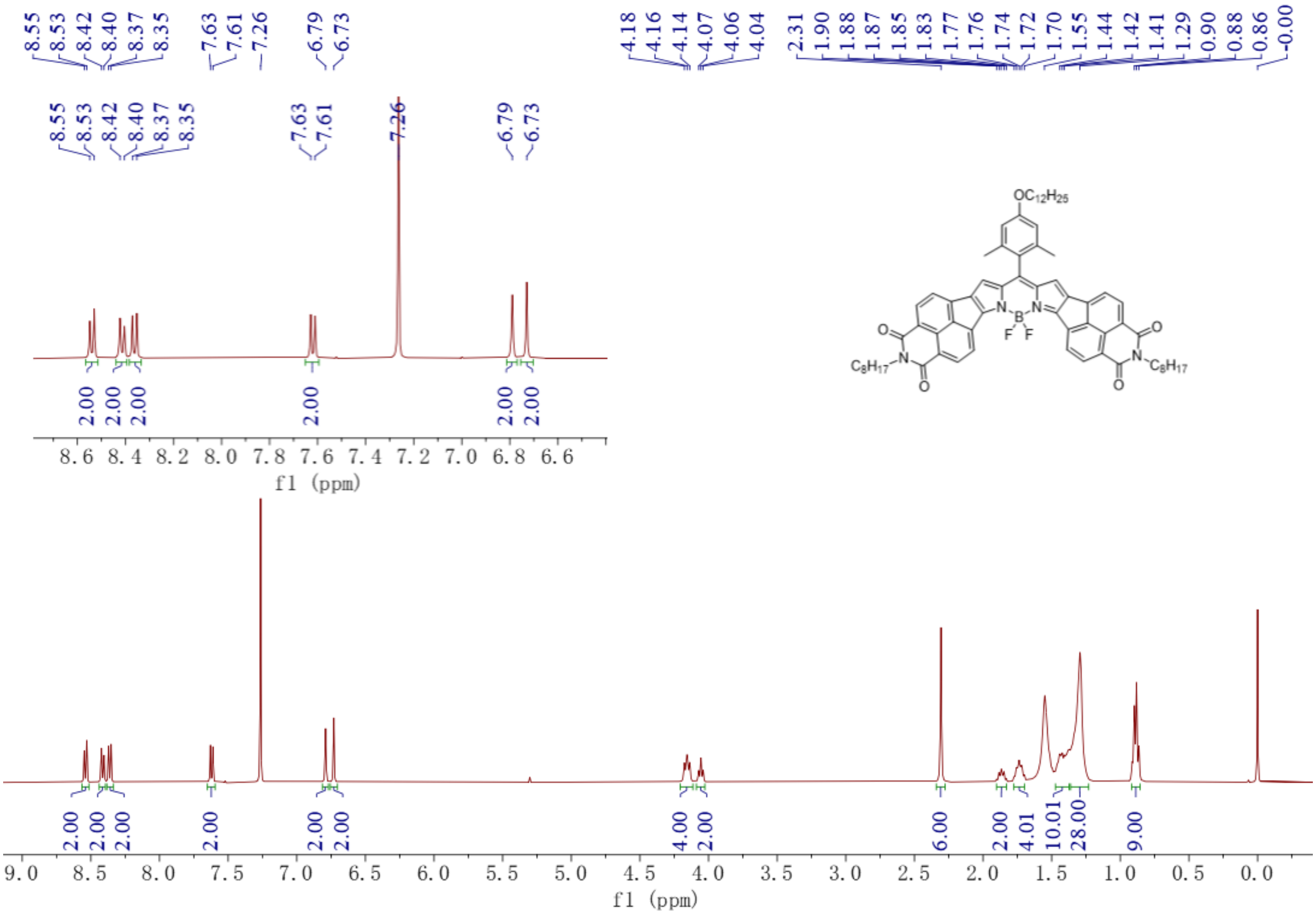

^{1}H NMR spectrum of **4b** in $CDCl_3$ (400 MHz)

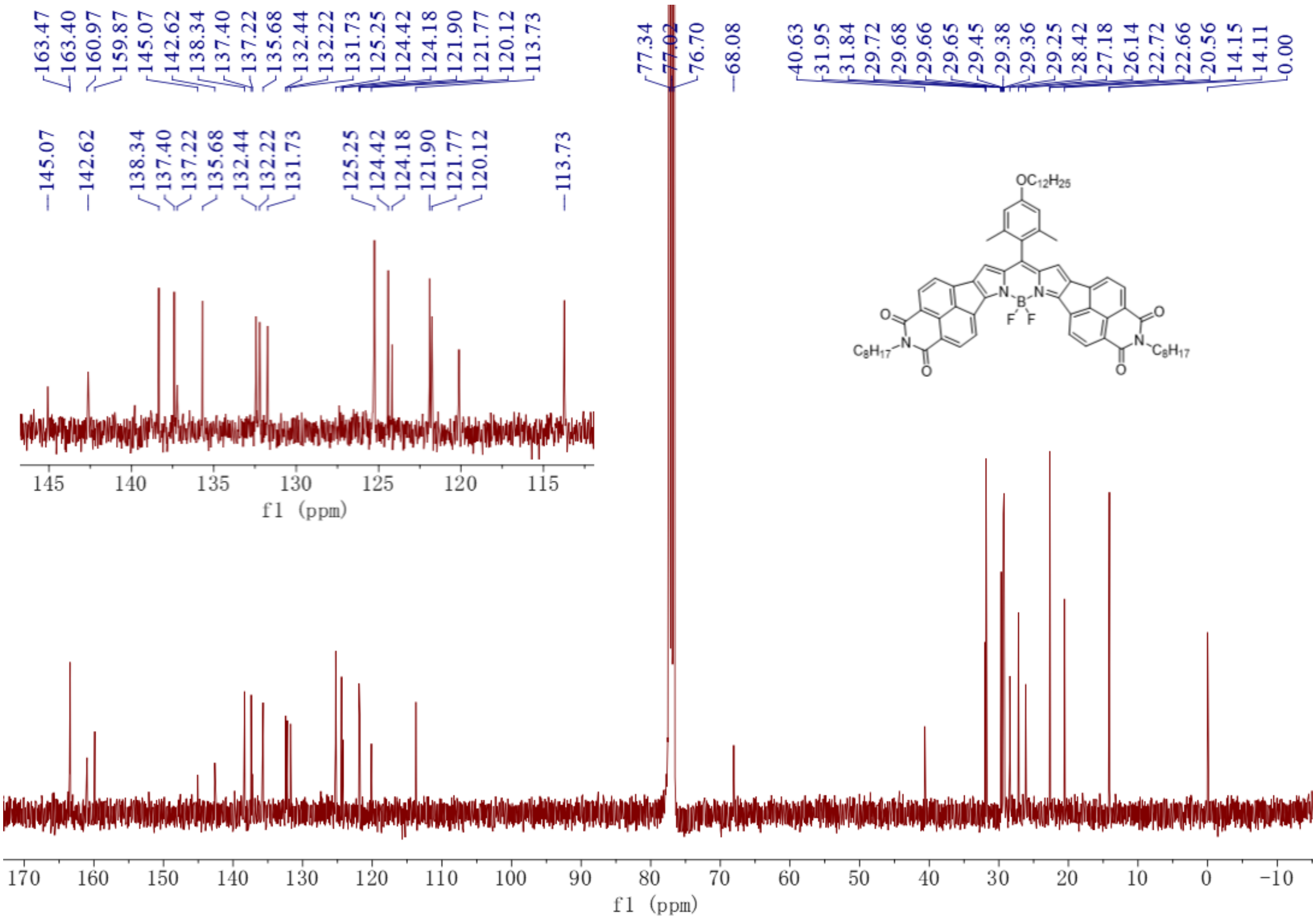

^{13}C NMR spectrum of **4b** in $CDCl_3$ (101 MHz)

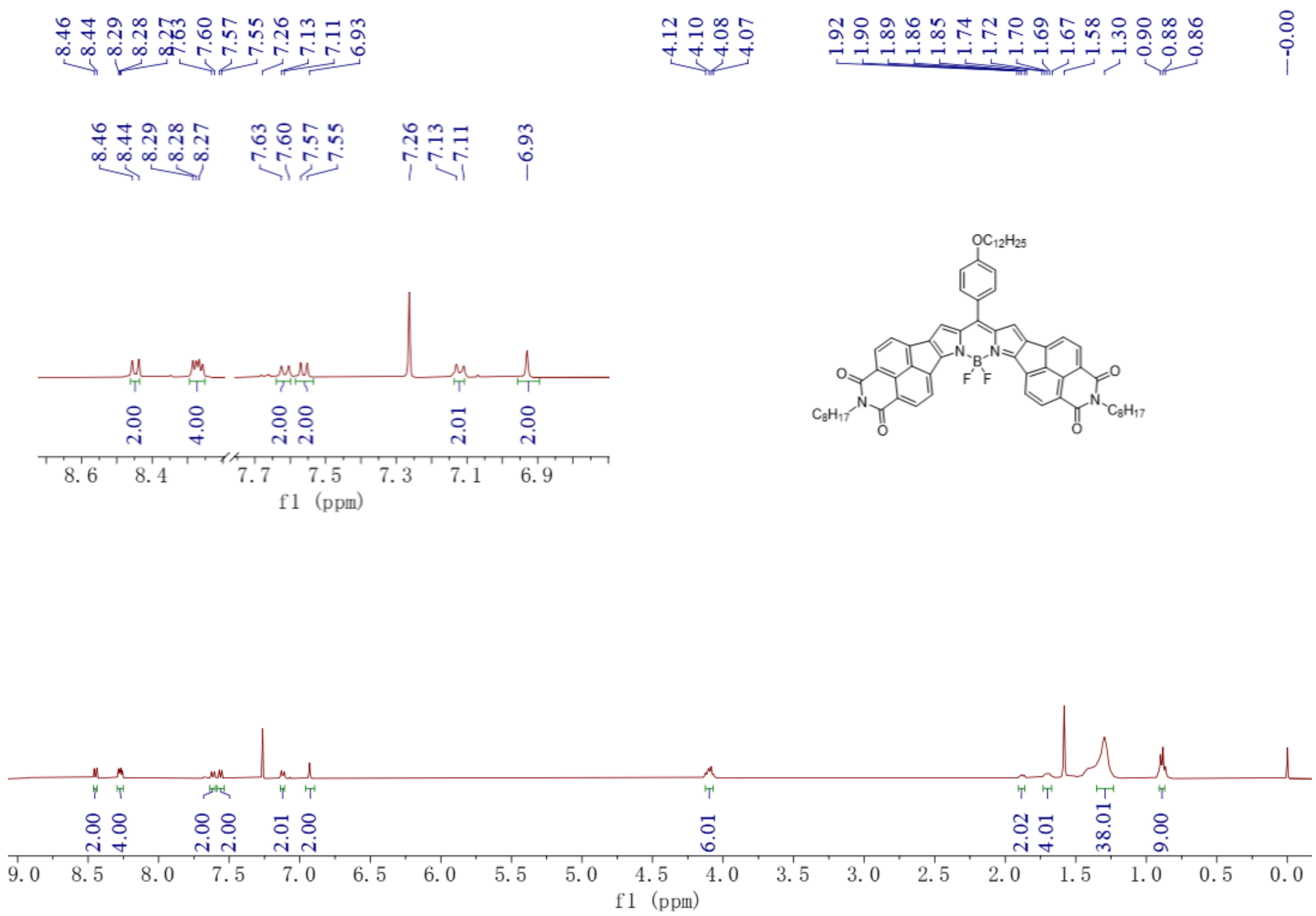

^{1}H NMR spectrum of **4c** in $CDCl_3$ (400 MHz)

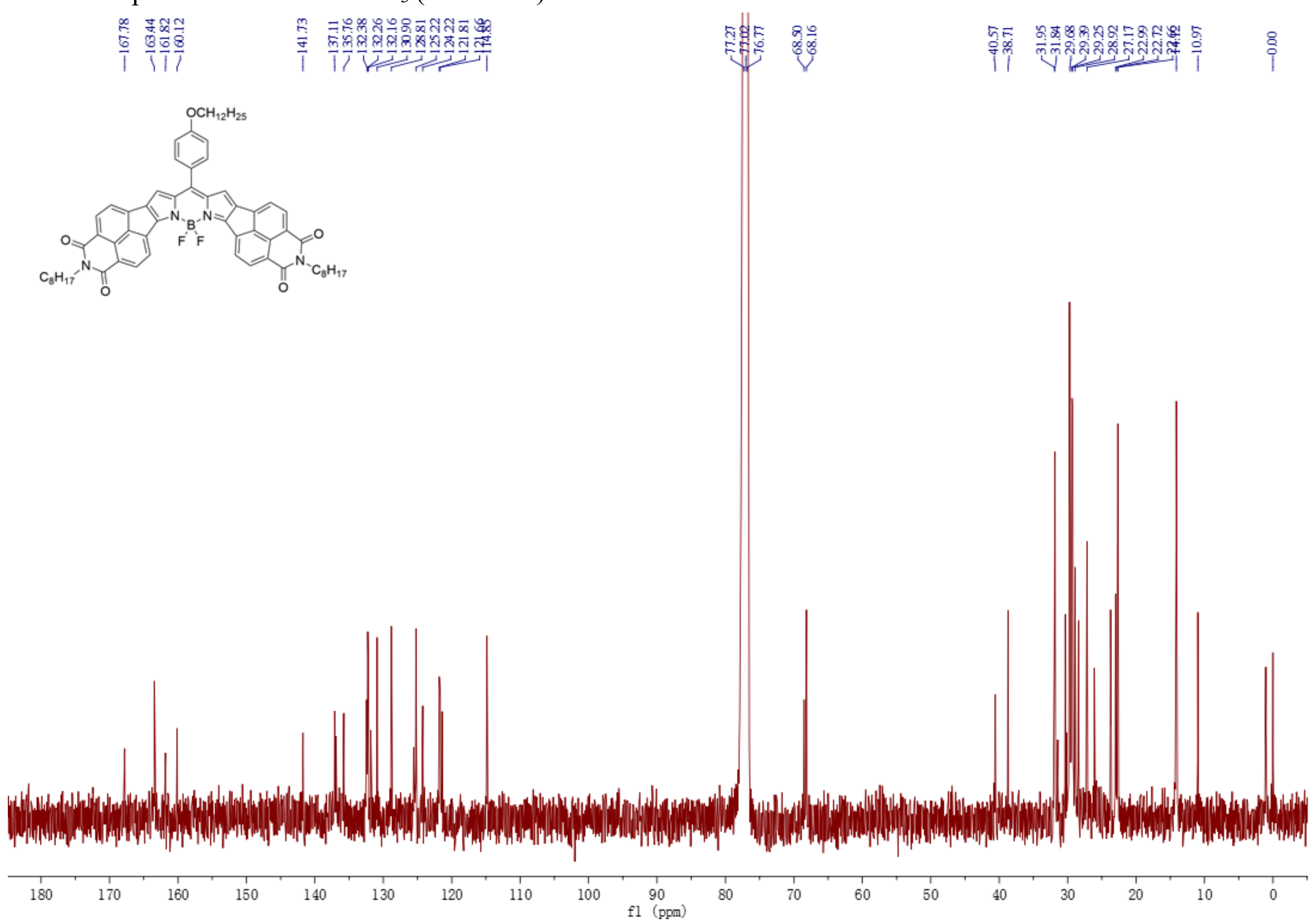

^{13}C NMR spectrum of **4c** in $CDCl_3$ (101 MHz)

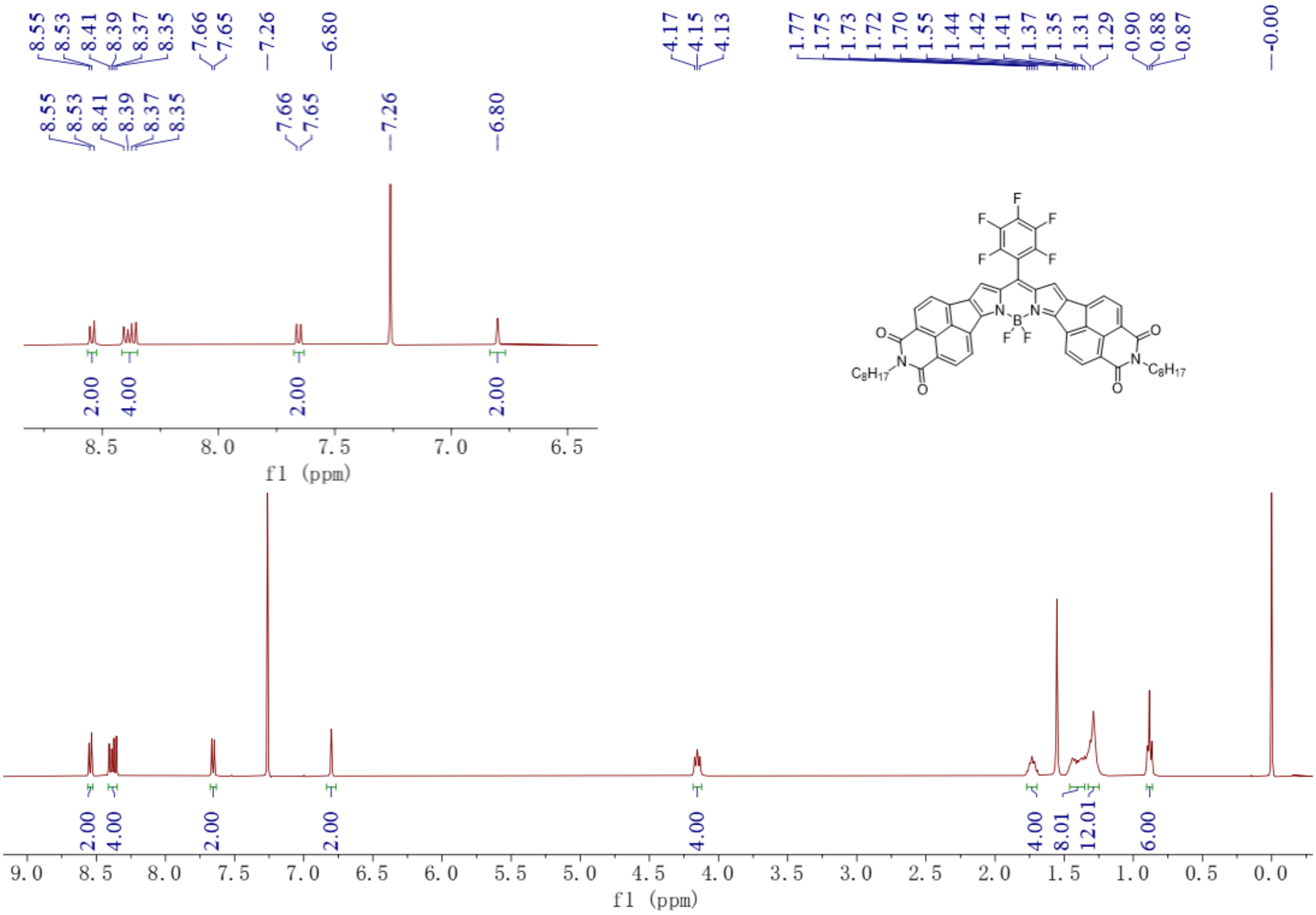

^{1}H NMR spectrum of **4d** in $CDCl_3$ (400 MHz)

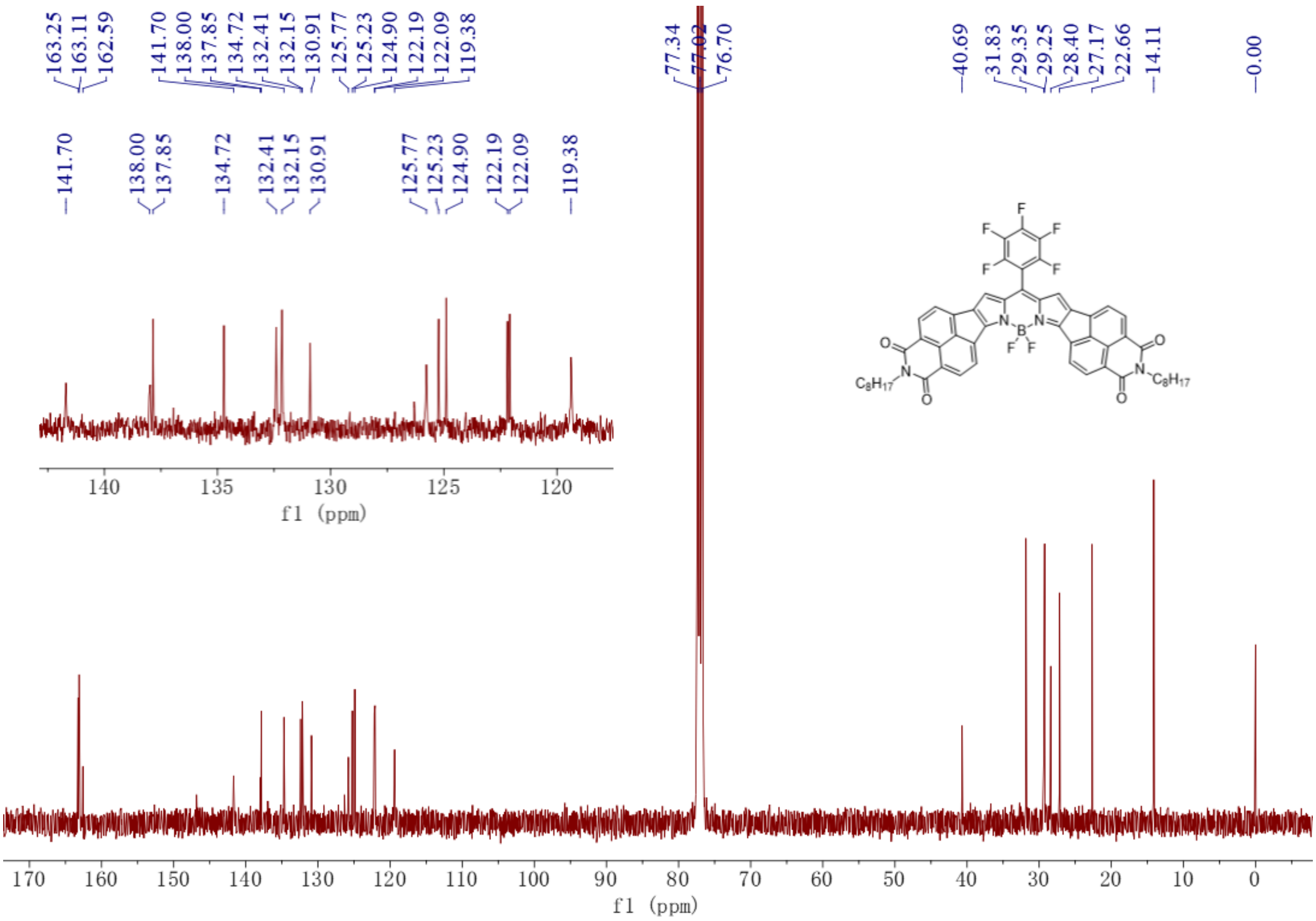

^{13}C NMR spectrum of **4d** in $CDCl_3$ (101 MHz)

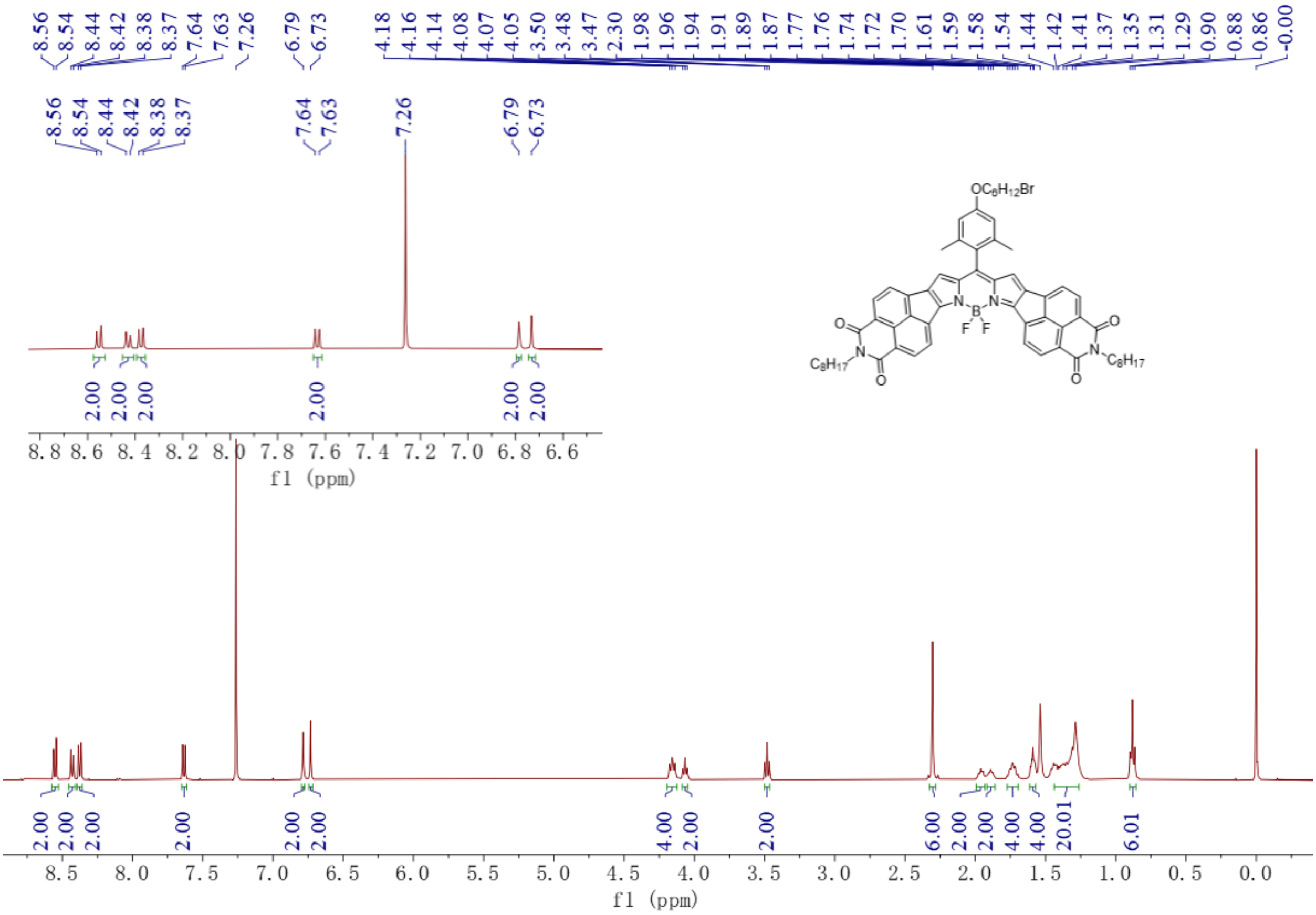

^{1}H NMR spectrum of **4e** in $CDCl_3$ (400 MHz)

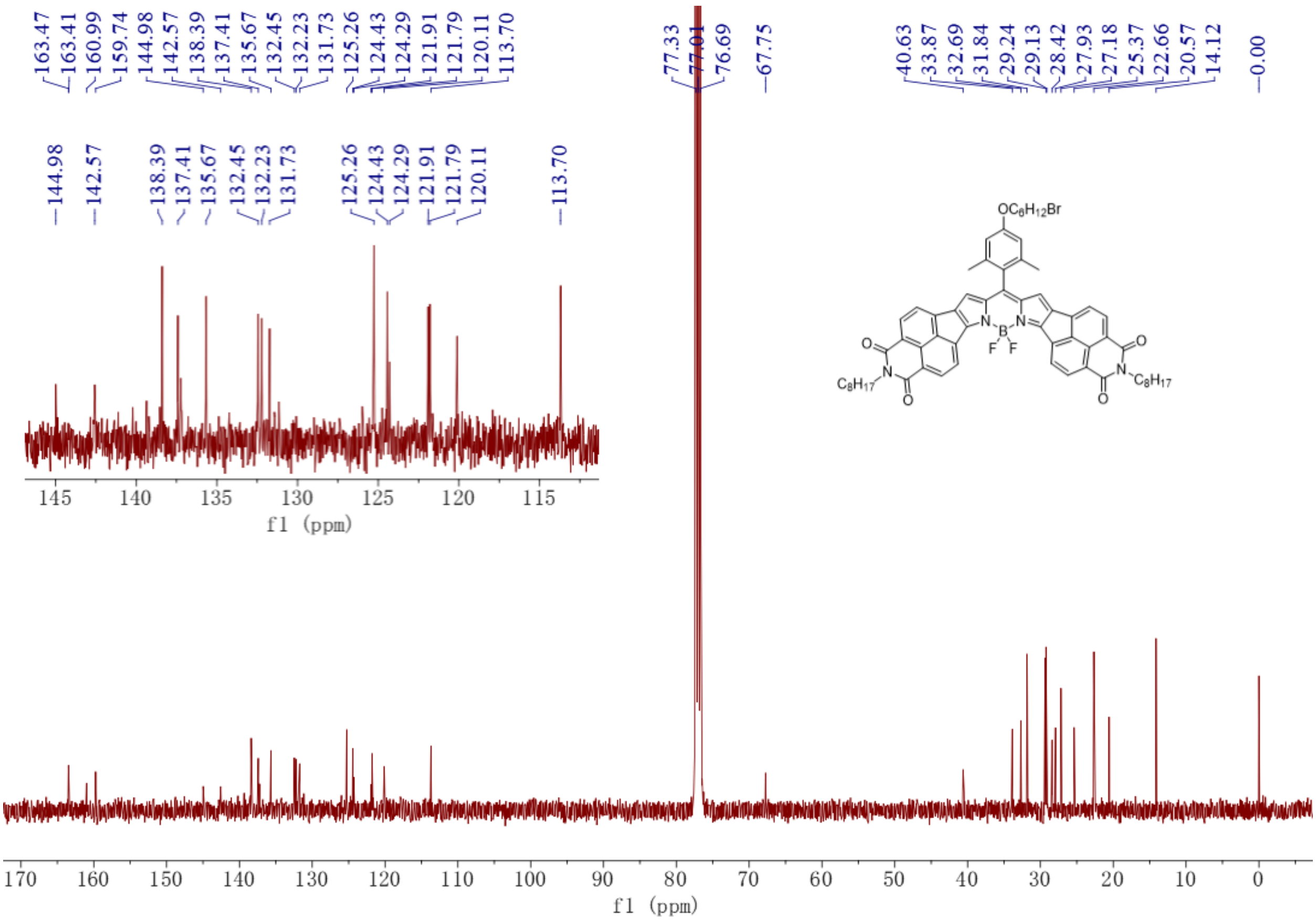

^{13}C NMR spectrum of **4e** in $CDCl_3$ (101 MHz)

10. High resolution mass spectra for all new compounds

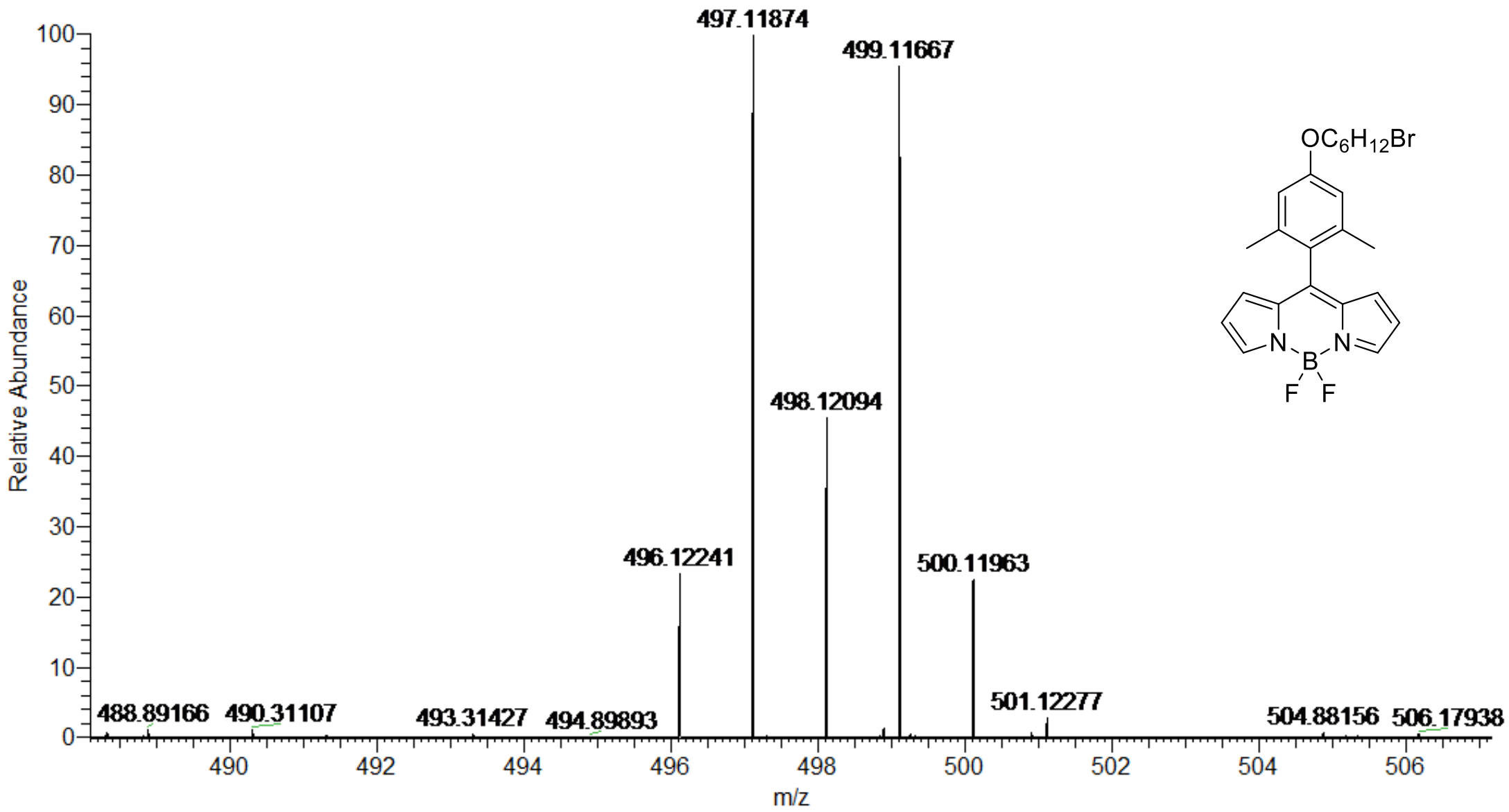

HRMS for **1e**

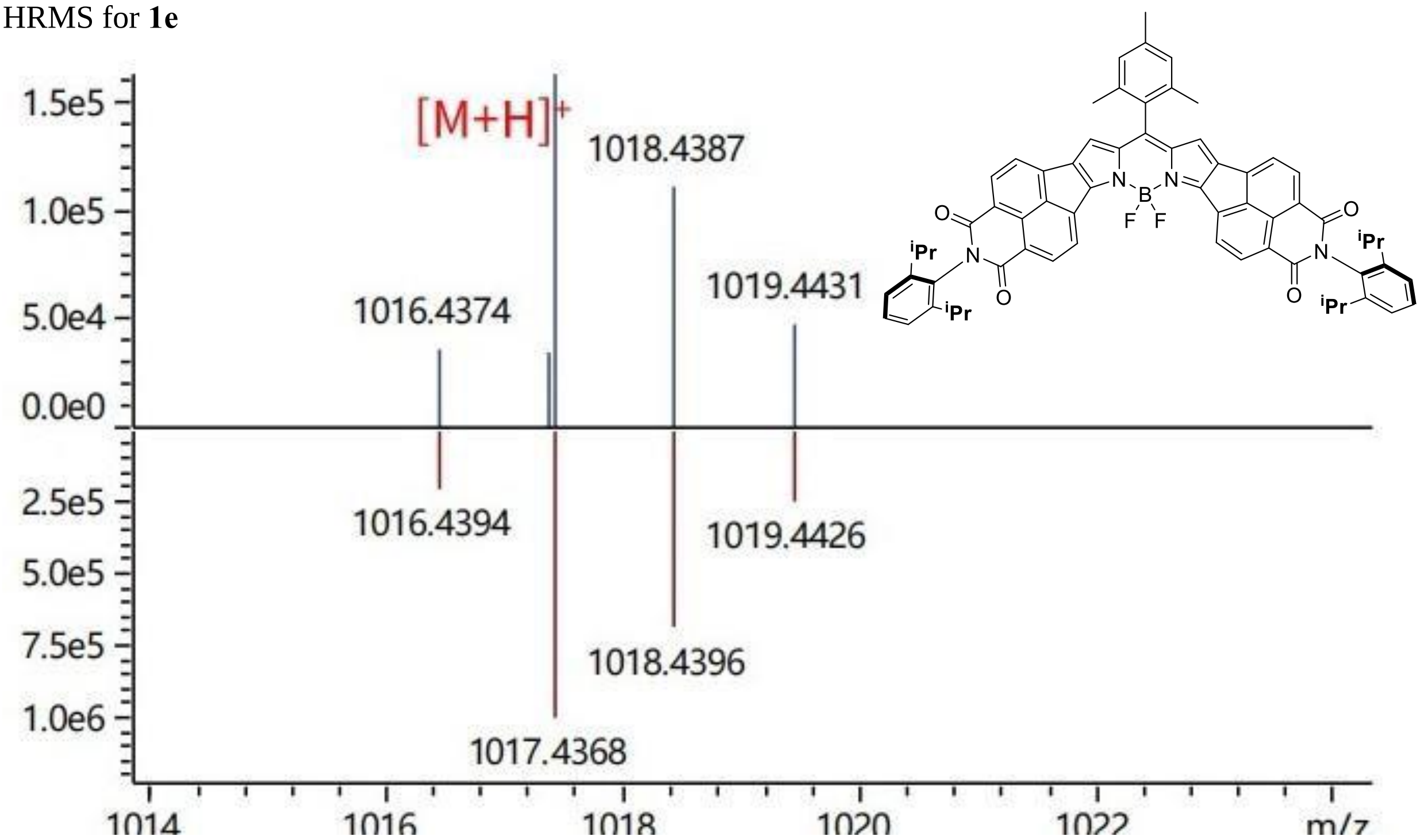

Observed (top) and calculated (bottom) HRMS for **3a**

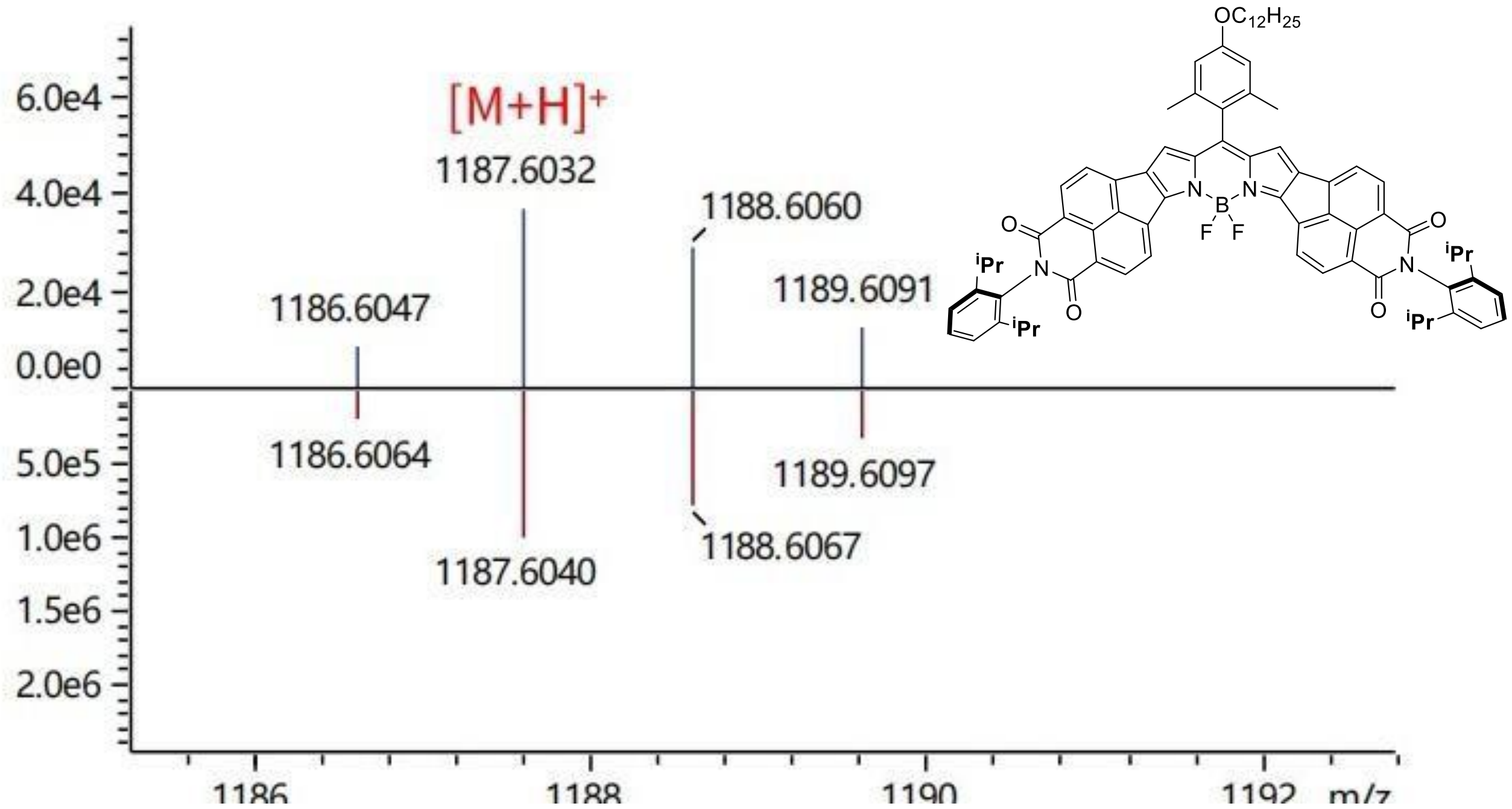

Observed (top) and calculated (bottom) HRMS for **3b**

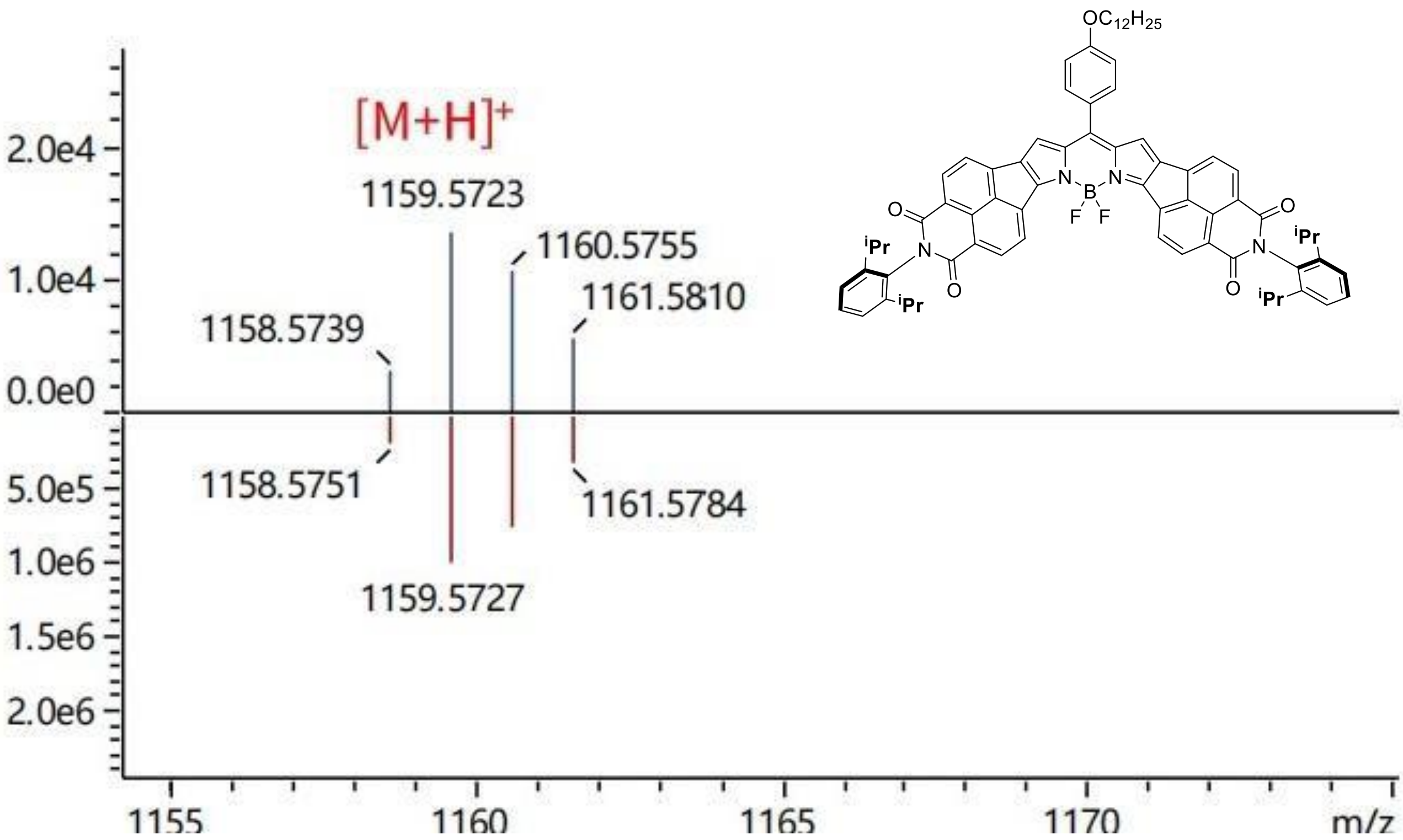

Observed (top) and calculated (bottom) HRMS for **3c**

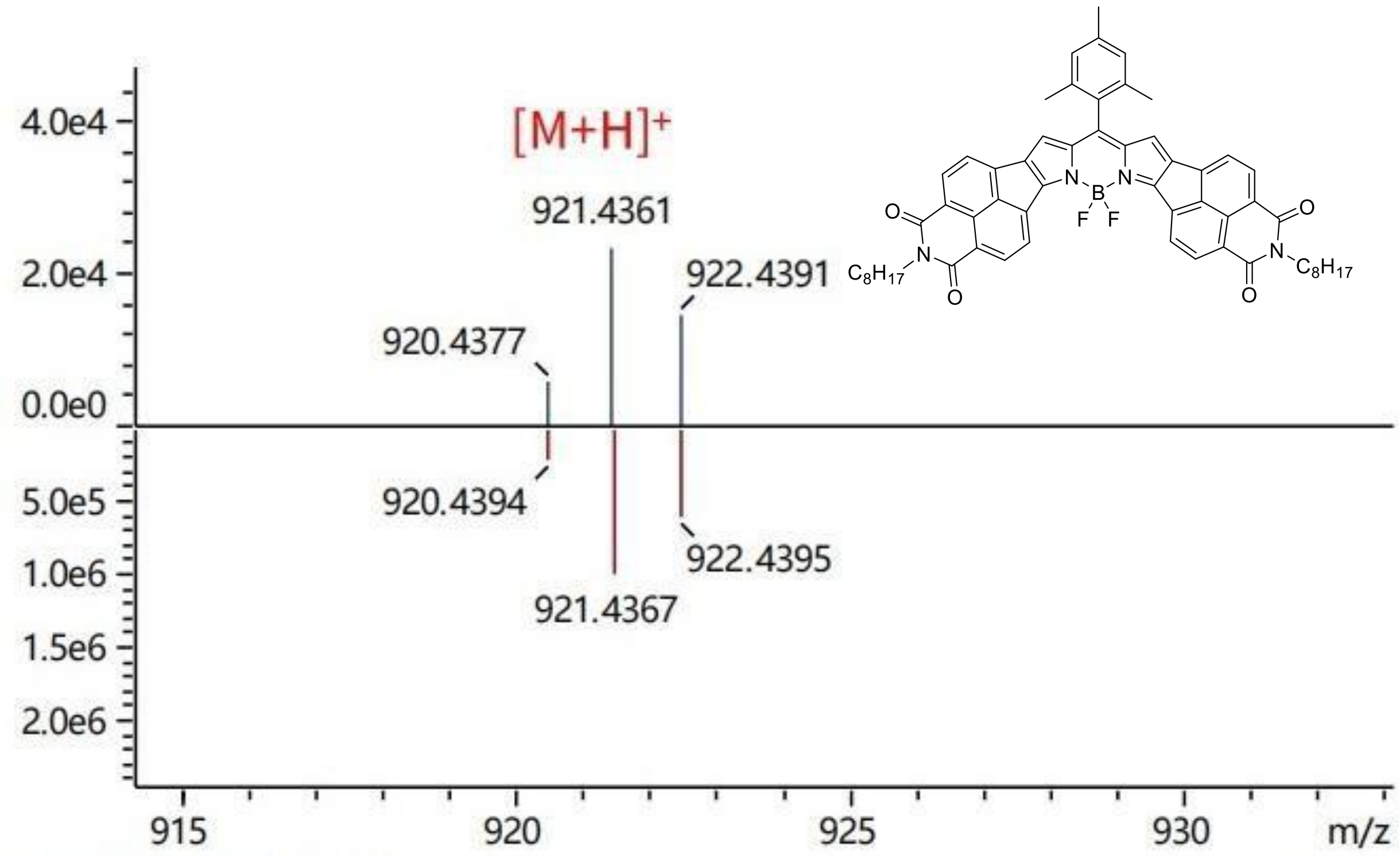

Observed (top) and calculated (bottom) HRMS for **4a**

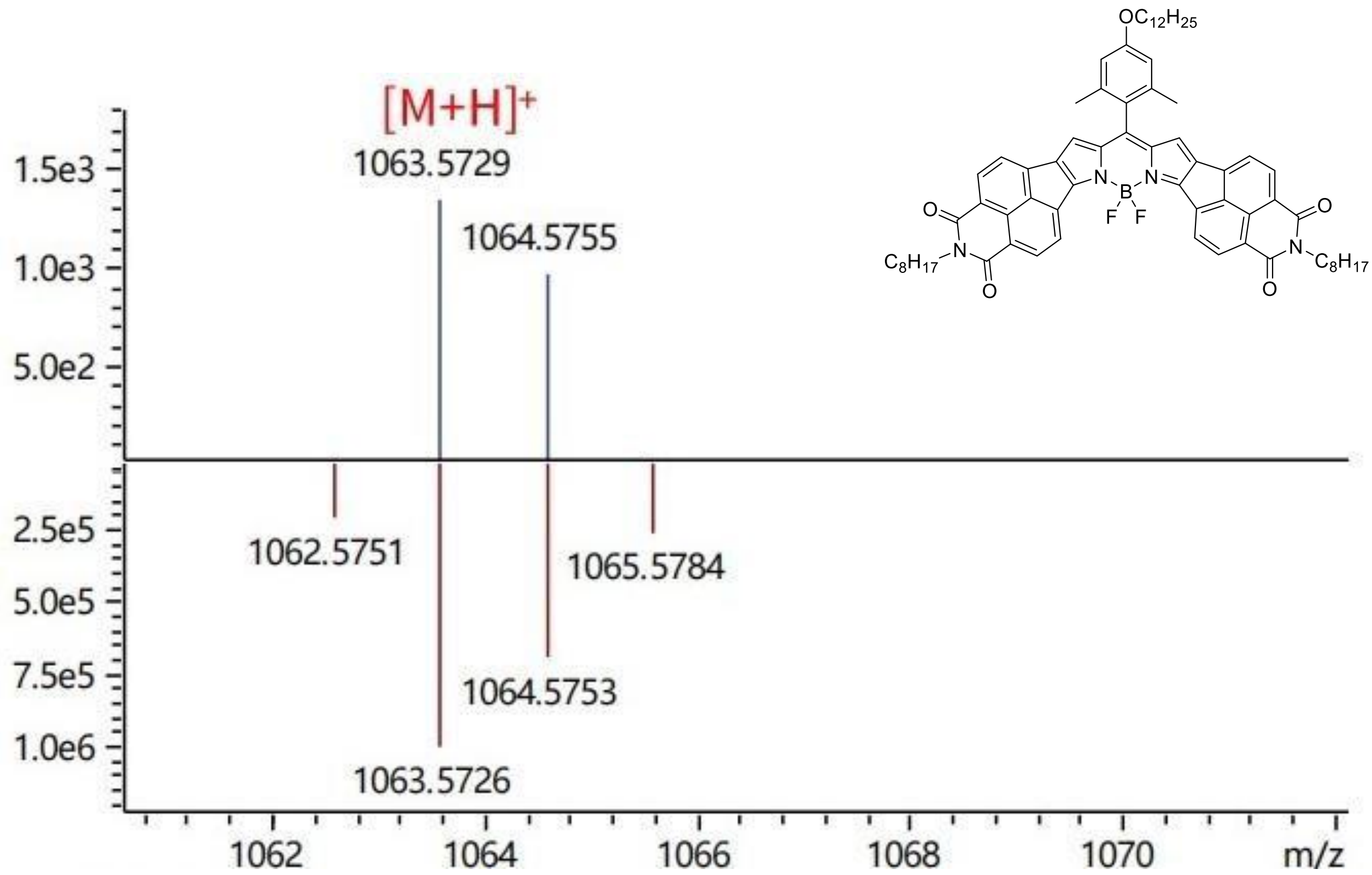

Observed (top) and calculated (bottom) HRMS for **4b**

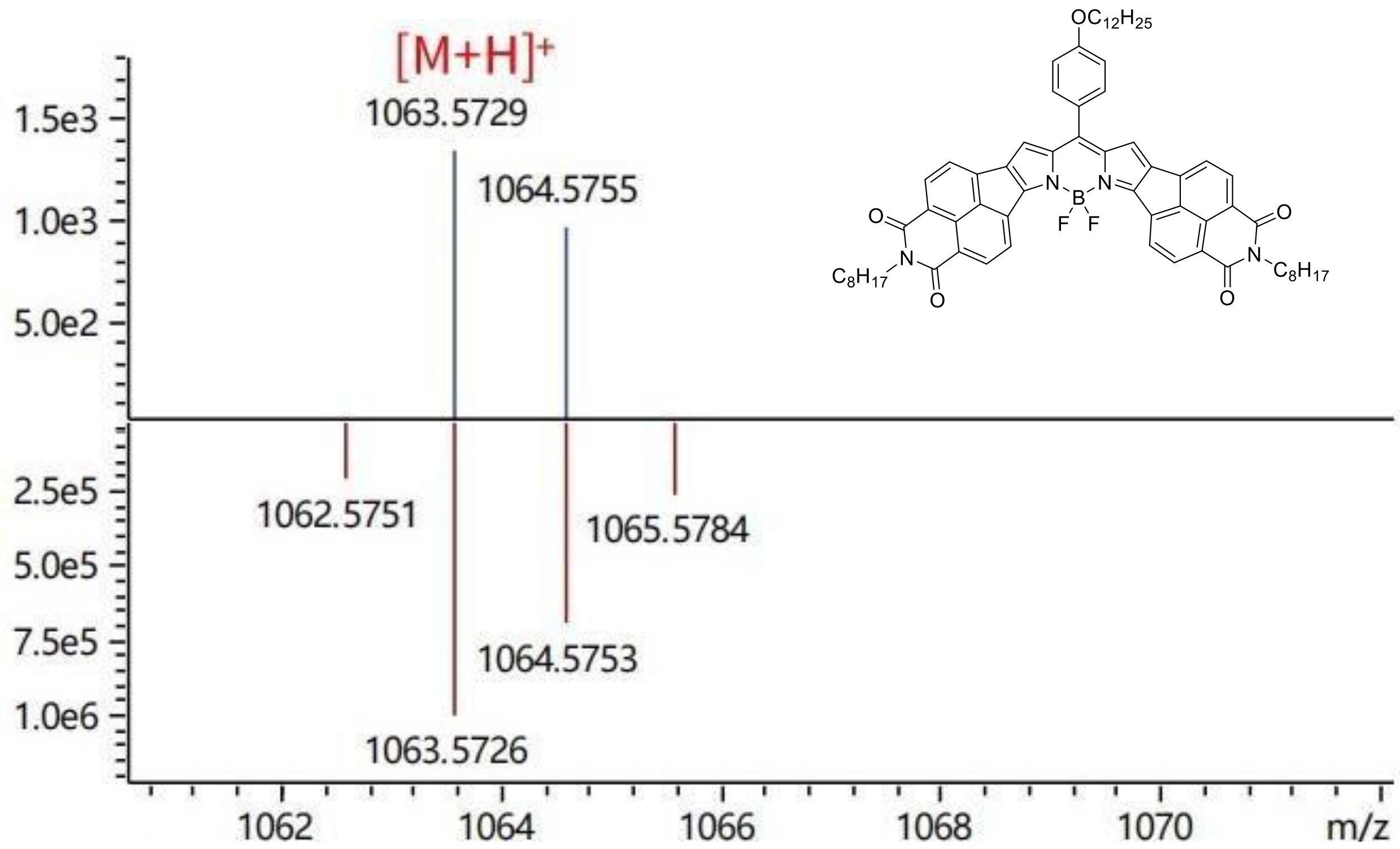

Observed (top) and calculated (bottom) HRMS for **4c**

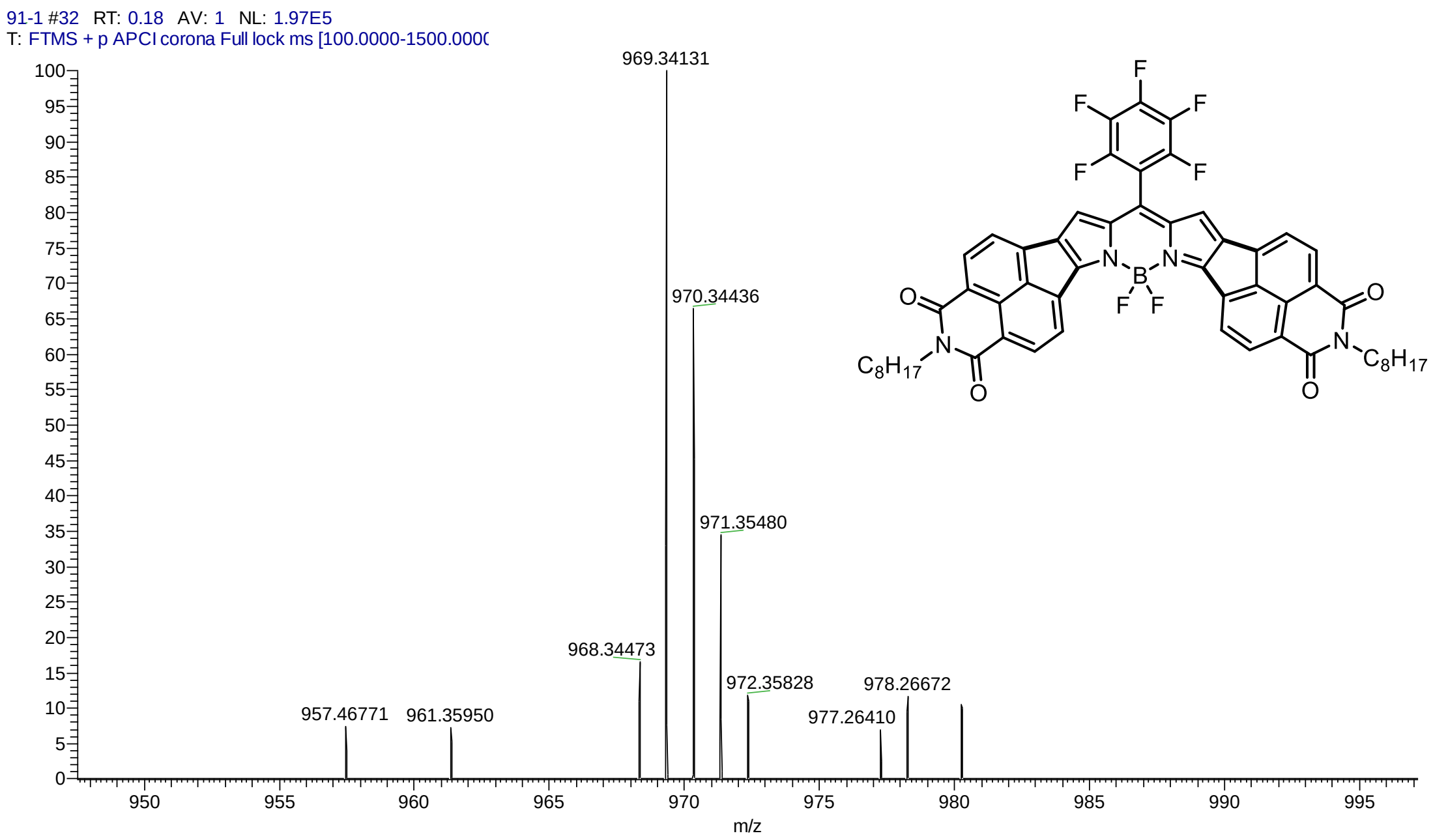

HRMS for **4d**

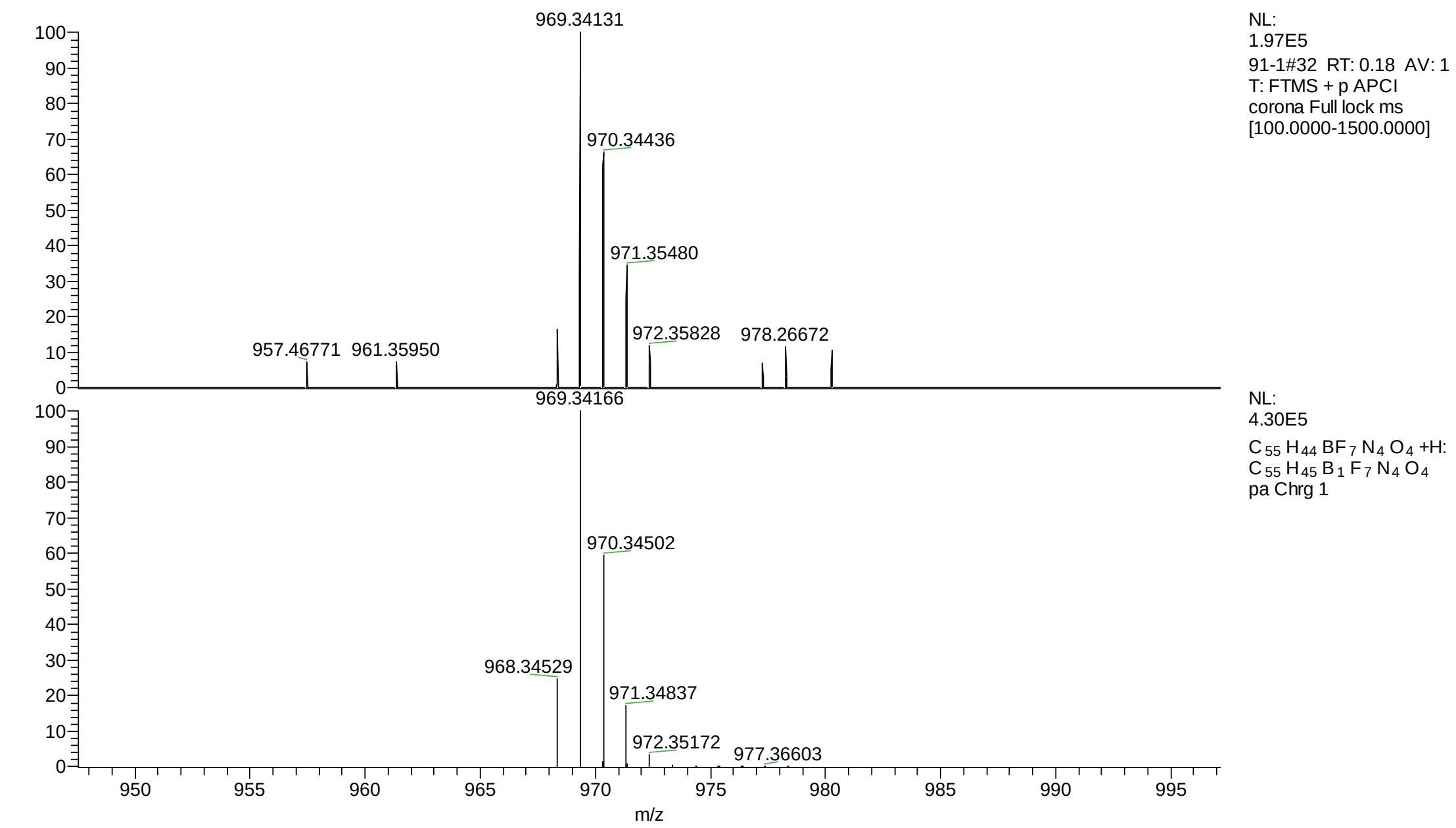

Observed (top) and calculated (bottom) HRMS for **4d**

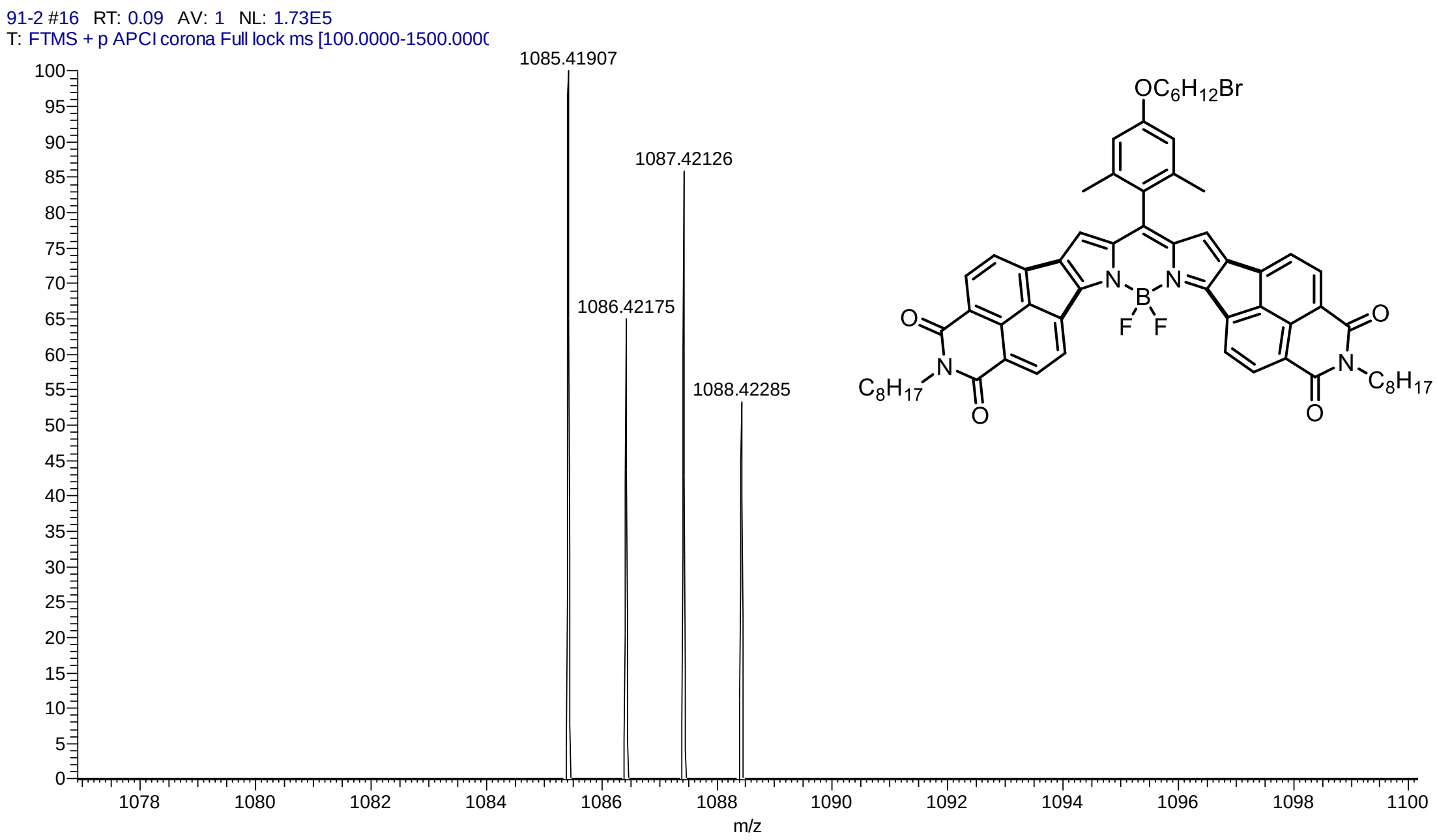

HRMS for **4e**

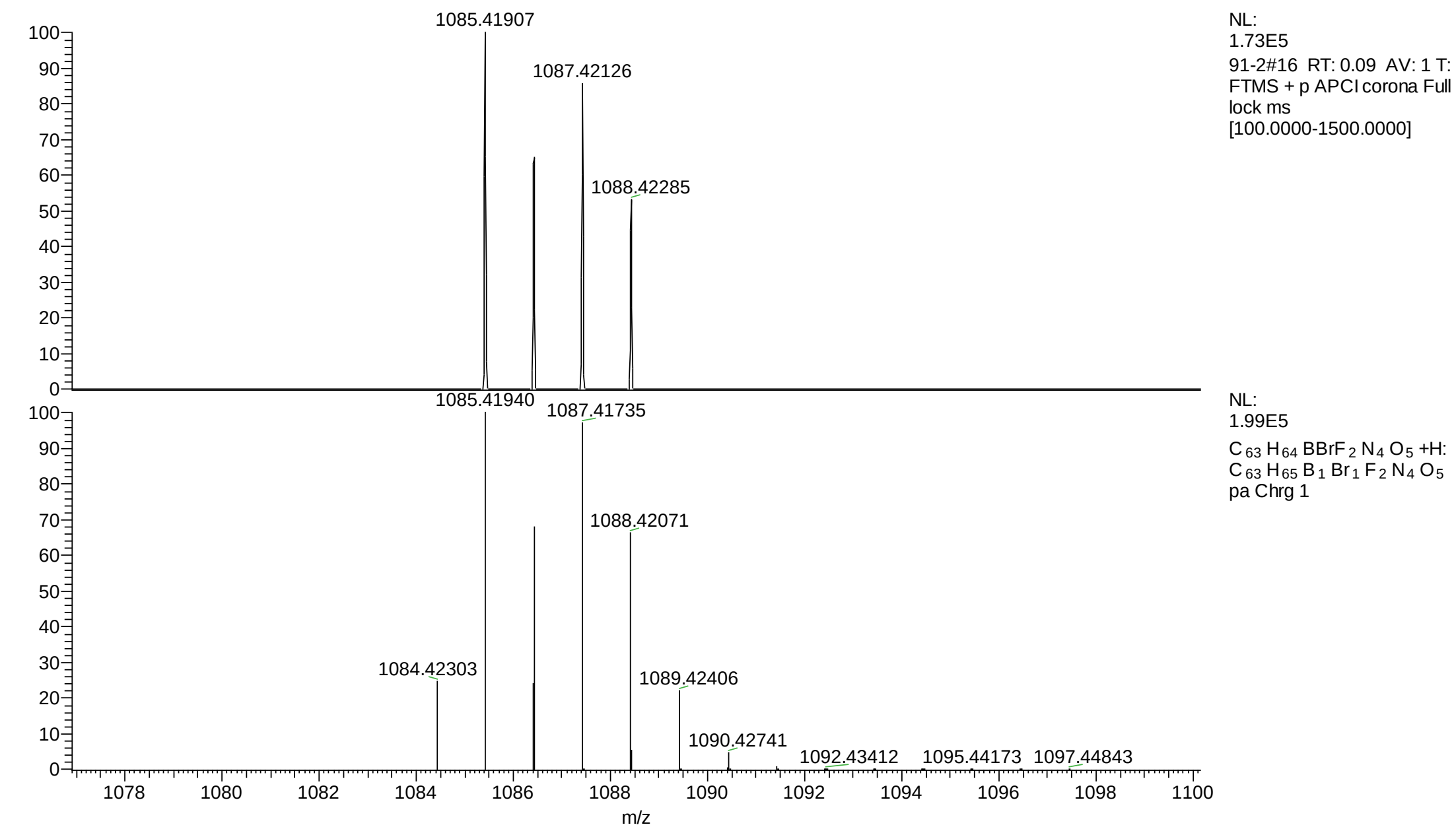

Observed (top) and calculated (bottom) HRMS for **4e**

11. Reference

1. Y. Kawamura, H. Sasabe and C. Adachi, Simple accurate system for measuring absolute photoluminescence quantum efficiency in organic solid-state thin films. *Jpn. J. Appl. Phys.,* 2004, **43**, 7729–7730.

2. Software for the integration of CCD detector system Bruker analytical X-ray systems, Bruker axs, Madison, WI (after 2013).

3. APEX2 suite for crystallographic software, Bruker axs, Madison, WI.

4. O. V. Dolomanov, L. J. Bourhis, R. J. Gildea, J. A. K. Howard and H. Puschmann, OLEX2: a complete structure solution, refinement and analysis program. *J. Appl. Cryst.,* 2009, **42**, 339–341.

5. G. M. Sheldrick, Crystal structure refinement with ShelXL. *Acta Cryst. C,* 2015, **71**, 3–8.

6. H. L. Kee, C. Kirmaier, L. Yu, P. Thamyongkit, W. J. Youngblood, M. E. Calder, L. Ramos, B. C. Noll, D. F. Bocian, W. R. Scheidt, R. R. Birge, J. S. Lindsey and D. Holten, *J. Phys. Chem. B,* 2005, **109**, 20433.

7. H. Golf, H. Reissig and A. Wiehe, *Org. Lett.,* 2015, **17**, 982.

8. N. Li, Z. Huang, X. Zhang, X. Song and Y. Xiao, *Anal. Chem.,* 2019, **91**, 15308–15316.

9. C. Pan, Z. Kang, F. Lv, X. Zhang, L. Zhang, L. Wang, J. Gao, E. Hao and L. Jiao, *Org. Lett.,* 2026, **28**, 1976–1982.

10. D. Uersfeld, S. Stappert, Chen. Li and K. Müllen, *Adv. Synth. Catal.,* 2017*,* **359**, 4184.

11. S. Kaloyanova, Y. Zagranyarski, S. Ritz, M. Hanulova, K. Koynov, A. Vonderheit, K. Müllen and K. Peneva, *J. Am. Chem. Soc.,* 2016, **138***,* 2881.

12. Z. Wang, C. Cheng, Z. Kang, W. Miao, Q. Liu, H. Wang and E. Hao, *J. Org. Chem.,* 2019, **84**, 2732–2740.

13. Gaussian 09, Revision D.01, M. J. Frisch, G. W. Trucks, H. B. Schlegel, G. E. Scuseria, M. A. Robb, J. R.; Cheeseman, G.; Scalmani, V.; Barone, B.; Mennucci, G. A.; Petersson, H.; Nakatsuji, M.; Caricato, X.; Li, H. P.; Hratchian, A. F.; Izmaylov, J.; Bloino, G.; Zheng, J. L.; Sonnenberg, M.; Hada, M.; Ehara, K.; Toyota, R.; Fukuda, J.; Hasegawa, M.; Ishida, T.; Nakajima, Y.; Honda, O.; Kitao, H.; Nakai, T.; Vreven, J. A.; Montgomery, Jr., Peralta, J. E.; Ogliaro, F.; Bearpark, M.; Heyd, J. J. Brothers, E.; Kudin, K. N.; Staroverov, V. N.; Keith, T.; Kobayashi, R.; Normand, J.; Raghavachari, K.; Rendell, A.; Burant, J. C.; Iyengar, S. S.; Tomasi, J.; Cossi, M.; Rega, N.; Millam, J. M.; Klene, M.; Knox, J. E.; Cross, J. B.; Bakken, V.; Adamo, C.; Jaramillo, J.; Gomperts, R.; Stratmann, R. E.; Yazyev, O.; Austin, A. J.; Cammi, R.; Pomelli, C.; Ochterski, J. W.; R. L.; Martin, Morokuma, K.; Zakrzewski, V. G.; Voth, G. A.; Salvador, P.; Dannenberg, J. J.; Dapprich, S.; Daniels, A. D.; Farkas, O.; Foresman, J. B.; Ortiz, J. V.; Cioslowski, J.; Fox, D. J. Gaussian, Inc., Wallingford CT, 2013.

14. W. Sheng, Y.-Q. Zheng, Q. Wu, Y. Wu, C. Yu, L. Jiao, E. Hao, J.-Y. Wang and J. Pei, *Org. Lett.,* 2017, **19**, 2893-2896.
